# Resting pulmonary function and artery pressure and cardiopulmonary exercise testing in chronic heart failure patients in Taiwan − a prospective observational cross-sectional study comparing healthy subjects and interstitial lung disease patients

**DOI:** 10.1080/07853890.2023.2228696

**Published:** 2023-06-30

**Authors:** Ming-Lung Chuang, Sung-Kien Sia, Kai-Wei Chang

**Affiliations:** a Division of Pulmonary Medicine and Department of Internal Medicine, Chung Shan Medical University Hospital, Taichung, Taiwan ROC; b School of Medicine, Chung Shan Medical University, Taichung, Taiwan ROC; c Department of Internal Medicine, Division of Cardiology, Chung-Shan Medical University Hospital, Taichung, Taiwan ROC

**Keywords:** Lung function, cardiopulmonary exercise testing, breathing pattern, pulmonary hypertension, ejection fraction

## Abstract

**Background:**

Restrictive ventilatory defects and elevated pulmonary artery pressure (PAP) are common in patients with chronic heart failure (CHF) and those with interstitial lung disease (ILD). However, as oxyhemoglobin desaturation seldom occurs in stable CHF patients at peak exercise, we hypothesized that the pathophysiology may be different between them. This study aimed to investigate: (1) PAP and lung function at rest, (2) pulmonary gas exchange (PGX) and breathing patterns at peak exercise, (3) mechanisms of dyspnea at peak exercise in patients with CHF compared to healthy subjects and ILD patients.

**Methods:**

We consecutively enrolled 83 participants (27 with CHF, 23 with ILD, and 33 healthy controls). The CHF and ILD groups had similar functional status. Lung function and cardiopulmonary exercise tests with Borg Dyspnea Score were performed. PAP was estimated using echocardiography. Resting lung function, PAP and peak exercise data in the CHF group were compared to the healthy and ILD groups. Correlation analysis was performed to elucidate the mechanisms of dyspnea in the CHF and ILD groups.

**Results:**

Compared to the healthy group, the CHF group had normal lung function, PAP at rest, and normal dyspnea score and PGX at peak exercise, whereas the ILD group had abnormal values compared to the CHF group. Dyspnea score was positively correlated with pressure gradient, lung expansion capabilities, and expiratory tidal flow in the CHF group (all *p* < 0.05), but inversely correlated with inspiratory time-related variables in the ILD group (all *p* < 0.05).

**Conclusion:**

Normal lung function and PAP at rest, and dyspnea scores and PGX at peak exercise indicated that pulmonary hypertension and fibrosis were insignificant in the patients with CHF. The factors affecting dyspnea at peak exercise were different between the CHF and ILD groups. As the sample size in this study was small, large-scale studies are warranted to confirm our findings.

## KEY MESSAGES

Normal lung function and pulmonary arterial pressure at rest, and dyspnea scores and pulmonary gas exchange at peak exercise indicated that pulmonary hypertension and fibrosis in the patients with chronic heart failure were not significant.

Dyspnea score was correlated with different physiological variables between the groups with chronic heart failure and interstitial lung disease.

## Introduction

Dyspnea and restrictive ventilatory defects are common in patients with chronic heart failure (CHF) with reduced and mildly reduced ejection fraction (HFrEF/HFmrEF) and interstitial lung disease (ILD) [1–8]. As a result, tidal volume (V_T_) is reduced and breathing frequency (B_f_) is increased.

However, the scenario may be not that simple, as V_T_ at high-intensity exercise has been reported to vary in patients with CHF [2,9–11], with reduced end-expiratory lung volume (EELV) at rest and during exercise [9,12]. In contrast, V_T_ is consistently reduced in patients with ILD at peak exercise [4,10]. As lung volumes are related to body height, V_T_ and EELV need to be adjusted. Moreover, to delineate restrictive ventilation defects, total lung capacity (TLC) is the preferred measure, as it does not change during exercise and is more accurate than forced vital capacity (FVC) [13,14]. Therefore, we hypothesized that V_T_/TLC and EELV/TLC could be used to examine lung expandability.

Similarly, the B_f_ in patients with CHF is different from that typically associated with restrictive ventilation limitation at peak exercise (i.e. ≥50 breaths/min) [2,4,10,15,16]. Moreover, breathing-related time variables such as inspiratory time (T_I_), expiratory time (T_E_), I:E ratio (E:I when appropriate), inspiratory duty cycle (IDC i.e. T_I_/total time of a breathing cycle [T_TOT_]) and rapid shallow breathing index (RBSI i.e. B_f_/V_T_ in L) have seldom been reported in CHF patients [9] or compared between patients with these two diseases [9,10], even though they may provide additional breathing information.

Pulmonary hypertension (i.e. groups 2 and 3) [17] and pulmonary fibrosis may occur in these two diseases [4,18,19]; however, exercise-induced oxyhemoglobin desaturation is not or seldom encountered in patients with CHF [11] or even in those with restrictive ventilatory defects [2,12], whereas it is often encountered in those with ILD [4,10,16]. Moreover, some studies have reported that patients with CHF did not have pulmonary fibrosis [20–23], and another study reported that 27% of patients with CHF with HFrEF/HFmrEF did not have pulmonary hypertension [24]. Hence, we hypothesized that lung function, pulmonary artery pressure (PAP) at rest, and pulmonary gas exchange (PGX) at peak exercise may be normal or mildly impaired in some patients with CHF.

Dyspnea is also common in these two patient populations; however, the differences in mechanisms are unclear. Therefore, we designed this study to compare: (1) PAP and lung function at rest; (2) PGX and breathing patterns at peak exercise between patients with CHF, ILD and healthy controls; and (3) dyspnea mechanisms at peak exercise in patients with CHF compared to those with ILD. This study may clarify physiological responses to exercise in these two patient populations, and help to develop more effective management strategies.

## Methods

### Study design

This was a prospective observational cross-sectional and single-blinded study, and it was approved by the Institutional Review Board of Chung Shan Medical University Hospital (CS2-19136 and CS2-21018). Written informed consent was provided by all participants.

### Subjects

We enrolled CHF and ILD patients with similar functional status from Chung Shan Medical University Hospital from 1 August 2017 to 31 December 2020. The inclusion criteria were: (1) age 40–80 years; (2) body mass index 18–30 kg/m^2^; (3) New York Heart Association (NYHA) class I–III and modified Medical Research Council (mMRC) score 0–3. An age range of 40–80 years was chosen because few young patients have CHF and ILD, and exercise performance in subjects older than 80 years may be different from that in younger subjects. In addition, as obesity confounds exercise physiology, obese subjects were excluded. A stable clinical condition for at least 1 month before participating in the study was required. The exclusion criteria were diabetes mellitus, uncontrolled hypertension, arrhythmia, cancer, liver, renal and lung diseases other than ILD, and anemia. As diabetes mellitus and CHF often co-exist, participants with well controlled diabetes mellitus were included in the CHF group. Participants in the ILD group could have autoimmune diseases but not CHF; those in the CHF group could not have autoimmune diseases. Physical activity was not limited or encouraged during the study period. None of the participants had contraindications for cardiopulmonary exercise tests (CPETs).

#### CHF

Participants with CHF who had reduced and mildly reduced EFs were referred by cardiologists. A left ventricular EF measured using two-dimensional echocardiography (2DLVEF) was performed within 2 months before or after commencing the study [25]. 2DLVEF <45–50% was considered to be reduced. CHF patients were excluded if their forced expired volume in one second (FEV_1_)/FVC was <0.7.

#### ILD

ILD was diagnosed by rheumatologists and pulmonologists according to standard criteria including idiopathic pulmonary fibrosis and autoimmune disease-related ILD [26,27]. All participants underwent high-resolution computed tomography (HRCT) within 1 year before study entry, and the diagnosis was confirmed by radiologists. All participants also underwent lung function tests within 3 months before enrollment, and they were only enrolled if their FEV_1_/FVC was >0.7 and FVC or TLC was <80% predicted.

#### Healthy controls

Healthy subjects were recruited from the local community and the hospital staff through personal contacts. They did not have known significant diseases. Data of the CHF group and healthy subjects were reported in part previously [28].

### Measurements

An oxygen-cost diagram (OCD) was used to assess daily functional activities. The subjects were asked to indicate a point on an OCD, a 10-cm long vertical line with everyday activities listed alongside the line, above which breathlessness limited them. The score was distance in cm from zero to the indicated point (a larger score indicating better daily functional activities). The chronic obstructive pulmonary disease assessment test **(**CAT) questionnaire was used to evaluate the quality of life in patients with chronic respiratory disease (a larger score indicating worse daily functional activities). Leisure activity was scored from 1 to 4 according to the hours of activity per week: 1= <1 h; 2 = 1–3 h; 3 = 3–6 h; 4= >6 h. All of the participants completed all functional ability assessments in this study.

Two-dimensional echocardiography was performed by two experienced technicians in all participants with CHF and ILD and in randomly selected controls, and reviewed by cardiologists who were blinded to all clinical data. Parasternal, apical and subcostal studies were conducted. PAP was estimated by tricuspid valve regurgitation maximal jet velocity (TRV) in cm/s, tricuspid valve regurgitation maximal pressure gradient (PG) in mmHg, and right ventricular systolic pressure (RVSP) in mmHg. RVSP was calculated as 4TRV^2^ + RAP, where RAP is the mean right atrial pressure estimated by looking at the size and ventilatory collapsibility of the inferior vena cava (IVC) on the echocardiogram. RAP = 3 mmHg when the IVC diameter is ≤21 mm and collapse with sniff is >50%; 8 mmHg when the IVC diameter and collapse with sniff are intermediate; and 15 mmHg when the IVC diameter is >21 mm and collapse with sniff is <50% [29].

FEV_1_, TLC, and residual volume (RV) were measured with a spirometer and body plethysmograph at body temperature, ambient atmospheric pressure, and fully saturated (BTPS). The best of three technically satisfactory readings was used. Diffusing capacity of the lungs for carbon monoxide (D_L_CO) was measured using the single-breath technique. Simple volume calibration was conducted with a 3-L syringe before each test. For the predicted race-adjusted values of lung function used by our institution, please refer to our previous studies [30,31]. To determine breathing reserve, direct maximum voluntary ventilation (MVV) was calculated from a 12-second rapid and deep breathing maneuver as recommended for all subjects [32].

#### Maximum CPET

The protocol was a 3-minute period of rest and a 3-minute period of unloaded cycling followed by a ramp-pattern exercise test to the limit of their tolerance. Work rate was selected at a slope of 5–20 watts per minute according to pre-determined fitness based on our derived protocol formula [33]. Inspiratory capacity (IC) was measured in all participants [34]. AT was determined using the dual method approach [35] including modified V-slope and ventilatory equivalents methods.

Twelve-lead electrocardiography, heart rate, oxyhemoglobin saturation (S_P_O_2_), oxygen uptake (V̇O_2_, ml/min, standard temperature and pressure, dry saturation, STPD), carbon dioxide output (V̇CO_2_, ml/min, STPD), minute ventilation (V̇_E_, L/min, BTPS), breathing frequency (B_f_, breath/min), and T_I_ and T_E_ in seconds were measured. Borg scores were obtained every 2 min during loaded exercise. Pneumotachograph and O_2_ and CO_2_ analyzers were calibrated with standard methods [36].

Maximum or good effort exercise was defined as either one of the following [35,37,38]: (1) heart rate reserve of 15% or 15 beats/min of predicted maximal heart rate or less; predicted maximum heart rate = 220–age; (2) respiratory exchange ratio of ≥1.05; (3) breathing reserve (BR) of ≤0.3; (4) decrease in S_P_O_2_ between unloaded exercise and peak exercise of ≥3%.

(1) $$\text{BR}=\text{1}-\text{V}{˙}_{\text{E}}/\text{MVV}$$

The techniques used for performing and accepting IC measurements were as previously reported [34,39]. Dynamic IC was measured at the end of a steady-state resting baseline and unloaded cycling, the middle of loaded exercise and end of exercise. The middle of loaded exercise was approximately 5–6 min after the start of loaded exercise, when it was assumed to be near AT. EELV was calculated as TLC minus dynamic IC. End-inspiratory lung volume (EILV) was calculated as tidal volume plus EELV.

N-terminal pro-brain natriuretic peptide (NT-proBNP) was measured using a fluorescence assay in all participants. The laboratory is qualified by the Taiwan Accreditation Foundation (ISO15189; site number 1817).

### Statistical analysis

The raw data are provided in the ‘Supplement data’ file. Data were summarized as mean ± standard error or median (inter-quartile range, IQR) as appropriate. The sample size was estimated to be at least 17 for each group when the population mean difference in TRV was 30 cm/s with a standard deviation of 30 for the CHF and ILD groups, and with a significance level of 0.05 and a power of 0.8. The unpaired *t*-test was used to compare means between two groups (i.e. the CHF group versus the healthy group, and the CHF group versus the ILD group). Correlations were based on Pearson’s correlation coefficients. A *p* value ≤ 0.05 was considered to be significant. Statistical analyses were performed using NCSS statistical software (NCSS 9, NCSS, LLC., Kaysville, Utah, USA).

## Results

**Figure 1.**
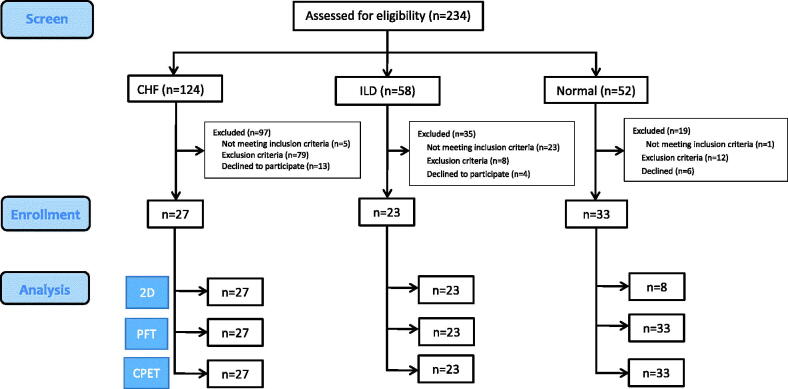
Flow chart. A total of 234 subjects were assessed for eligibility. Twenty-seven patients with chronic heart failure (CHF), 23 with interstitial lung disease (ILD), and 33 healthy controls were enrolled. All of the subjects underwent lung function tests within 3 months before enrollment. All of the patients with CHF had exertional dyspnea, LVEF <45–50%, and a forced expired volume in one second (FEV_1_)/forced vital capacity (FVC) of >0.7. ILD patients were enrolled only if their FEV_1_/FVC was >0.7 and their FVC and TLC were <80% predicted. The healthy controls were free of known significant diseases. A total of 151 subjects were excluded due to the reasons shown. For details about the inclusion and exclusion criteria of the participants, please refer to the text. 2D: two-dimensional echocardiography; PFT: pulmonary function testing; CPET: cardiopulmonary exercise testing.

A total of 234 subjects were screened, of whom 83 were enrolled, including 27 with CHF, 23 with ILD, and 33 normal controls after excluding 151 subjects due to the reasons shown in Figure 1. The CHF group were younger, had a higher body mass index and OCD score and lower CAT score, even though they consumed more cigarettes than those with ILD (Table 1). The CHF group had a higher NT-proBNP level; however, there was no difference between the CHF group and ILD group.

**Table 1.** Demographic data, blood test, and questionnaire scores in the chronic heart failure (CHF) and interstitial lung disease (ILD) groups and normal subjects.

	CHF (27)		ILD (23)		Normal (33)		CHF vs. ILD	CHF vs. normal
Group (*N*)	Mean	SE	Mean	SE	Mean	SE	*p* Value	*p* Value
Age, years	57.7	1.9	**64.5**	2.1	61.8	1.7	0.04	NS
Sex, M:F	27:0		9:14		33:0			
BMI, kg/m^2^	26.6	0.6	**23.6**	0.6	24.8	0.5	0.001	0.047
Cigarette, pack-year	**28.9**	3.8	9.8	4.2	2.9	3.5	0.003	<0.0001
Borg score @ rest	0.4	0.1	0.5	0.1	0.0	0.1	NS	0.03
NYHA score, 1–4	1.5	0.1	1.6	0.1	1.0	0.1	NS	0.0001
mMRC score, 0–4	0.5	0.1	0.7	0.1	0.0	0.1	NS	0.006
Leisure activity, 0–4	1.6	0.1	1.8	0.2	1.6	0.1	NS	NS
CAT summed score, 0–40	3.8	0.8	**8.7**	0.9	0.5	0.7	<0.0001	0.006
OCD score, 0–10 cm	7.5	0.2	**6.9**	0.2	8.3	0.2	0.05	0.01
NT-proBNP, pg/mL	**358.3**	60.4	219.9	66.5	45.3	6.3	NS	<0.0001

*Abbreviations*: CAT: chronic obstructive pulmonary disease assessment test; mMRC: modified Medical Research Council; NYHA: New York Heart Association; OCD: oxygen-cost diagram. Leisure activity was coded 1–4 according to hours of activity per week: 1 = <1 h; 2 = 1–3 h; 3 = 3–6 h; 4 = >6 h. Bolded numbers indicating more advanced or impaired between the disease groups.

The CHF group had reduced LVEF% and normal TRV, PG, and RVSP, whereas the ILD group had normal LVEF% and elevated TRV, PG, and RVSP (Table 2). The CHF group had normal spirometry, lung volumes, and D_L_CO% and its derivatives, whereas PEF%, D_L_CO% and V_A_% in the CHF group were lower than in the controls. The ILD group had greater reductions in FVC%, FEV_1_%, PEF%, TLC%, and D_L_CO% and its derivatives, and greater elevations in RV/TLC and RV/TLC% than the CHF group. Both disease groups had normal FEV_1_/FVC.

**Table 2.** Echocardiographic and lung function data in the chronic heart failure (CHF) and interstitial lung disease (ILD) groups and normal subjects.

	N	CHF		ILD		Normal		CHF vs. ILD	CHF vs. NORMAL
	CHF:ILD:Normal	Mean	SE	Mean	SE	Mean	SE	*p* Value	*p* Value
LVEF, %	27:20:8	**41.6**	1.3	62.3	1.3	61.3	2.2	<0.0001	<0.0001
Max velocity, TR, cm/s	27:20:8	249.3	6.7	**279.7**	10.4	227.9	14.1	0.01	NS
Max PG, TR, mmHg	27:19:8	24.2	0.9	**31.9**	2.6	21.3	2.8	0.003	NS
RVSP, TR, mmHg	27:18:8	34.0	1.9	**40.6**	3.2	28.8	3.9	0.06	NS
FVC, % predicted	27:23:33	93.4	2.7	**67.4**	2.9	101.8	2.5	<0.0001	0.06
FEV_1_, % predicted	27:23:33	94.9	2.6	**70.6**	2.8	102.7	2.4	<0.0001	0.07
FEV_1_/FVC, %	27:23:33	**81.4**	1.1	86.1	1.2	79.8	1.0	0.01	NS
MMEF, % predicted	27:23:33	88.6	5.1	74.3	5.5	92.6	4.6	NS	NS
PEF, % predicted	27:23:33	86.4	3.5	**70.0**	3.7	104.0	3.1	0.005	0.0007
TLC, % predicted	27:23:33	93.4	2.3	**75.7**	2.4	97.2	2.0	<0.0001	NS
RV/TLC, %	27:23:33	39.1	1.4	**50.9**	1.6	38.7	1.3	<0.0001	NS
RV/TLC, % predicted	27:23:33	106.6	3.3	**128.0**	3.6	101.4	3.0	<0.0001	NS
D_L_CO, % predicted	27:23:33	89.4	3.1	**47.4**	3.4	106.2	2.8	<0.0001	0.0002
D_L_CO/V_A_, % predicted	27:23:33	104.2	3.5	**73.8**	3.9	103.7	3.2	<0.0001	NS
V_A_, % predicted	27:23:33	78.1	2.1	**58.4**	2.3	86.8	1.9	<0.0001	0.008

*Abbreviations*: D_L_CO: diffusing capacity for carbon monoxide of lung; FEV_1_: forced expired volume in one second; FVC: forced vital capacity; LVEF: left ventricular ejection fraction; Max: maximum; MMEF: maximum mid-expiratory flow; PEF: peak expiratory flow; RV: residual volume; RVSP: right ventricular systolic pressure; TLC: total lung capacity; TR: tricuspid valve regurgitation. LVEF in the CHF group including heart failure with reduced and mildly reduced ejection fractions: <30%, *n* = 3; ≥30%− <40%, *n* = 4; ≥40%− <50%, *n* = 20, LVEF in normal controls: *n* = 8, all >50%, (61.3 ± 2.1%); RVSP = 4 V^2^ + RAP, where V: peak TR jet velocity; RAP: mean right atrial pressure. Bolded numbers indicating more impaired when compared between the disease groups.

At peak exercise, compared to the controls, the CHF group had normal leg fatigue, dyspnea scores, S_P_O_2_, ventilation efficiency (V̇_E_/V̇O_2_ and V̇_E_/V̇CO_2_), breathing reserve, lung expansion-related variables, breathing-related time variables, and oxygen pulse% (Table 3, all p = NS). Of note, the CHF group had lower V̇O_2_%, work rate%, HR%, pulse pressure, V_T_/T_I_, V_T_/T_E_ and V̇_E_ than the controls (all *p* < 0.05). At peak exercise, compared to the CHF group, the ILD group had a higher dyspnea score, tachypnea (shorter T_I_ and T_E_) and rapid-shallow breathing, lower breathing reserve, ventilation, and lung expansion, and worse pulmonary gas exchange.

**Table 3.** Cardiopulmonary exercise variables at peak exercise unless specified.

	CHF (27)		ILD (23)		Normal (33)		CHF vs ILD	CHF vs Normal
Group (*N*)	Mean	SE	Mean	SE	Mean	SE	*p* Value	*p* Value
Symptoms								
Borg leg fatigue	6.3	0.4	6.9	0.4	6.5	0.4	NS	NS
Borg dyspnea	5.3	0.4	**7.2**	0.4	5.8	0.4	0.006	NS
Exercise capacity related variables								
V̇O_2_, % predicted	72.9	3.6	74.78	3.8	91.6	3.2	NS	0.0004
Work rate, % predicted	82.0	4.0	84.04	4.3	115.5	3.6	NS	<0.0001
Effort related variables								
RER	1.06	0.02	1.05	0.02	1.16	0.02	NS	<0.01
Heart rate, %predicted	83.0	2.2	84.4	2.3	95.2	1.9	NS	0.0001
Pulse pressure, mm Hg	71.7	6.0	83.0	6.3	103.2	5.3	NS	0.0003
Ventilation and its limit related variables								
Breathing reserve, %	46.2	3.1	**32.1**	3.3	39.1	2.7	0.007	NS
V̇_E_, L/min	57.7	3.4	**45.5**	3.6	71.4	3.0	0.04	0.01
V_T_/T_I_ , L/s	2.1	0.1	**1.6**	0.1	2.6	0.1	0.005	0.02
V_T_/T_E_, L/s	1.8	0.1	**1.4**	0.1	2.2	0.1	0.08	0.01
Ventilation efficiency and gas exchange related variables								
SpO_2_, %	96.8	0.9	**87.0**	1.0	96.7	0.8	<0.0001	NS
V̇_E_/V̇O_2_ @AT	27.6	1.1	**32.6**	1.2	28.0	1.0	0.008	NS
V̇_E_/V̇CO_2_ @AT	33.1	1.1	**37.8**	1.2	31.7	1.0	0.01	NS
V̇_E_/V̇O_2_	34.9	1.3	**39.7**	1.4	34.4	1.1	0.03	NS
V̇_E_/V̇CO_2_	37.2	1.6	42.0	1.7	39.6	1.5	NS	NS
Tidal volume expansion related variables								
EELV/TLC	0.51	0.03	0.51	0.03	0.48	0.03	NS	NS
V_T_-EELV/TLC	0.83	0.02	0.84	0.02	0.81	0.02	NS	NS
V_T_/TLC	0.32	0.01	**0.28**	0.01	0.33	0.01	0.05	NS
Breathing time related variables								
B_F_, breath/min	31.6	1.8	**41.7**	1.9	36.4	1.6	0.001	NS
T_I_, s	0.91	0.04	**0.74**	0.04	0.81	0.04	0.008	NS
T_E_, s	1.09	0.06	**0.80**	0.06	0.97	0.05	0.002	NS
RSBI, breath/L	18.1	2.3	**41.9**	2.5	19.3	2.1	<0.0001	NS
IDC	0.46	0.01	0.48	0.01	0.46	0.01	NS	NS
E: I	1.22	0.04	1.10	0.05	1.18	0.04	NS	NS

*Abbreviations*: AT: anaerobic threshold; B_F_: breathing frequency; CHF: chronic heart failure; EELV: end-expiratory lung volume; E:I: expiratory time (T_E_) and inspiratory time (T_I_) ratio; IDC: inspiratory duty cycle: T_I_/breathing cycle time; ILD: interstitial lung disease; O_2_P: oxygen pulse: oxygen uptake (V̇O_2_) in mL/heart rate in beat/min; RER: respiratory exchange ratio; RSBI: rapid shallow breathing index: B_F_/V_T_ in L; SpO_2_: oxyhemoglobin saturation; TLC: total lung capacity; V̇CO_2_: CO_2_ output; V̇_E_: minute ventilation; V_T_: tidal volume in L; V_T_-EELV: end-inspiratory lung volume. Bolded number indicating more impaired between the disease groups.

In the CHF group, dyspnea score at peak exercise was positively correlated with PG, FVC%, IC%, EILV/TLC, and V_T_/T_E_ (Table 4, all *r* = 0.41 − 0.42, all *p* < 0.05), whereas it was inversely correlated with I:E and IDC in the ILD group (*r* = −0.41 and −0.43, respectively, both *p* ≤ 0.05).

**Table 4.** Correlations of dyspnea score @ peak exercise with breathing pattern and gas exchange at peak exercise or specified otherwise and with echocardiography and lung function at rest in patients with chronic heart failure (CHF) and those with interstitial lung disease (ILD).

	CHF		ILD	
	*r*	*p* Value	*r*	*p* Value
Left ventricular ejection fraction, %		NS		NS
Max velocity, TR, cm/s		NS		NS
Max PG, TR, mmHg	0.42	0.03		NS
FVC%	0.41	0.04		NS
IC%	0.41	0.04		NS
D_L_CO%		NS		NS
V̇_E_/V̇O_2_ @ anaerobic threshold		NS		NS
V̇O_2_, % predicted		NS		NS
Heart rate, % predicted		NS		NS
S_P_O_2_, %		NS		NS
V̇_E_/V̇CO_2_		NS		NS
V_T_/T_I_, L/s		NS		NS
V_T_/T_E_, L/s	0.41	0.04		NS
EILV/TLC	0.35	0.08		NS
EELV/TLC		NS		NS
V_T_/TLC		NS		NS
Breathing frequency, breath/min		NS		NS
RSBI, breath/L		NS		NS
T_E_, s		NS		NS
T_I_, s		NS	−0.40	0.06
I:E		NS	−0.41	0.05
IDC		NS	−0.43	0.04

For abbreviations, please refer to Tables 2 and 3.

## Discussion

Our results showed that the CHF and ILD groups had the same reduced functional status according to the study design and presented with the same reduced peak exercise capacity, but that the CHF group had higher OCD and lower CAT scores. The CHF group had normal resting lung function, TRV, PG, and RVSP at rest, and breathing pattern and PGX at peak exercise, whereas the ILD group had reduced lung function and elevated TRV, PG, and RVSP at rest, and abnormal breathing pattern and PGX at peak exercise. These findings do not support that CHF leads to restrictive ventilatory defects or pulmonary hypertension at rest and restrictive ventilatory patterns at peak exercise.

### Symptoms and functional status

The CHF and ILD groups had similar dyspnea scores at rest and functional status with the same peak exercise capacity, and both groups could perform daily leisure activities at a similar level to the normal controls (Table 1). However, the OCD and CAT scores were better in the CHF group than in the ILD group. Of note, a CAT score ≥5 has been reported to be a risk factor for nonfatal cardiac events in patients with CHF [28]. Moreover, OCD and CAT have been reported to be better than other clinical assessment tools for patients with chronic obstructive pulmonary disease, and they are currently the clinical assessment tools of choice [40]. In the CHF group, there was no correlation between NT-proBNP and NYHA score (*r* = 0.01, P = NS). It is worth noting that the NT-proBNP levels were not highly elevated in the CHF group, as they were in a chronic stable condition, as defined by the inclusion criteria (Table 1).

### Echocardiography

The CHF group had reduced LVEF according to the study design, and presented with normal TRV, PG, and RVSP (Table 2). Thus, the patients with CHF in this study did not present with either the precapillary or post-capillary type of group 2 pulmonary hypertension [19,41,42]. LVEF was correlated with D_L_CO% (*r* = 0.39, *p* = 0.04) and AT% (*r* = 0.44, *p* = 0.02), but not with D_L_CO/V_A_% or V̇O_2peak_% (both p = NS), suggesting that LVEF is more related to pulmonary capillary blood volume and moderate exercise intensity than alveolar membrane conductance and peak exercise intensity.

### Lung volumes at rest

Restrictive ventilatory defects may occur in patients with CHF, as FEV_1_ and vital capacity are either normal or proportionately reduced [11] and can be caused by cardiomegaly itself [7,43], pulmonary congestion, interstitial fibrosis [6,44], and ventilatory muscle weakness. Several studies have reported that restrictive ventilatory defects in patients with CHF may increase inspiratory elastic and resistive work of breathing due to increased airway and lung tissue hysteresivity and decreased compliance of the lungs and chest wall [4,9]. Despite these reports, the incidence and types of lung function impairment may vary. For example, restrictive ventilatory defects have been reported in half of patients with severe CHF [1], and they have been reported to progress rapidly in incipient and early cases [3]. In addition, severe cases (i.e. LVEF ≤30%) may have normal spirometry, restrictive alone, and mixed ventilatory defects [2]. In the current study, the CHF group had normal lung function as only three CHF patients had a LVEF ≤30%. Nevertheless, LVEF was not correlated with FVC% (p = NS) in the current study.

### D_L_CO%

D_L_CO% has been reported to be reduced in patients with CHF and to be an independent predictor of V̇O_2peak_% [45]. The reduction in D_L_CO is due to impaired Cl^-^ and Na^+^ pumping in alveolar epithelium, peri-bronchial edema causing hypoventilation, and exercise altering hemodynamics [46]. However, D_L_CO% was normal in the patients with CHF in the current study, and it was correlated with LVEF due to a pulmonary blood volume effect at moderate exercise intensity as mentioned above. In addition, D_L_CO% was not correlated with V̇O_2peak_% (p = NS), probably due to not reflecting peak peripheral oxygen extraction. Moreover, D_L_CO% was not correlated with lung volumes or flows (maximum mid-expiratory flow%, peak expiratory flow%, V̇_Epeak_, V_T_/T_Ipeak_, and V_T_/T_Epeak_) in the current study (all p = NS). In contrast, Agostoni et al. reported that D_L_CO, membrane diffusion, capillary blood volume, and alveolar volume were lowest in patients with severe CHF, and were related to V̇O_2peak_ (D_L_CO, *r* = 0.577) [1]. The discrepancies between the two studies may be due to differences in the case number of severe CHF and the criteria used for the severity of CHF (LVEF% in the present study versus V̇O_2peak_, mL/min/kg in Agostoni’s study).

### Lung volumes at peak exercise

Patients with CHF have been reported to present with a restrictive ventilation pattern during exercise (i.e. poor tidal volume expansion and rapid shallow breathing frequency) [9,11,15,16]. The mechanisms are through lung restriction and elevated resistive and inspiratory elastic work of breathing as mentioned above [4,9], and may be reflected in V_Tpeak_ and EELV_peak_ [9,12]. However, V_Tpeak_ has been reported to vary [2,10], and V_Tpeak_/TLC, EELV_peak_/TLC, and breathing pattern were normal in the patients with CHF in the current study (Table 3). V_Tpeak_/TLC is an inverse marker of dynamic lung hyperinflation in patients with chronic obstructive pulmonary disease [34]. The lung expansion data suggest that dynamic lung hyperinflation does not occur in patients with CHF.

### V̇_E_, breathing-related time variables, and PGX at peak exercise

Cross et al. reported that V_T_/T_I_ was normal but V_T_/T_E_ was low in patients with CHF, because V_T_ and T_I_ were commensurately reduced whereas T_E_ was normal, resulting in a lower V_T_/T_E_ value [9]. However, Johnson et al. [12] have reported that expiratory flow constrains the tidal flow-volume responses to graded exercise in relation to the maximal flow-volume envelope. Although we did not use Johnson’s technique to confirm expiratory flow constraint, the reduced V̇_E_, V_T_/T_I_, and V_T_/T_E_ in the patients with CHF in our study may be due to reduced exercise effort at peak exercise, resulting in reduced exercise capacity, heart rate, and pulse pressure, but not due to ventilation impairment/limitation (because of normal breathing reserve and normal spirometry). It is possible that the expiratory flow may not be constrained in our CHF participants, as they had normal FVC% and better LVEF% compared to the previous study (93.4 ± 2.7% versus 76 ± 4% and 41.6 ± 1.3% versus 24 ± 2%, respectively) [12]. Furthermore, the current study had a larger number of CHF patients (27 versus 11 in the previous study). However, further studies are necessary to clarify these issues. All breathing-related time variables were normal in the patients with CHF in the current study, which is consistent with a previous report in which T_I_/T_TOT_ was normal [47]. Although interstitial fibrosis may cause restrictive ventilation in patients with CHF, its role may be minor, as oxyhemoglobin desaturation rarely occurred during exercise in the current and previous reports [2,10,16]. Although pulmonary congestion may cause oxyhemoglobin desaturation during exercise, it usually occurs in the acute phase [16]. In contrast, pulmonary vasculopathy and ILD often cause oxyhemoglobin desaturation when exercising [4,10,15,48]. In summary, compared to the patients with ILD, those with CHF had better tidal volume expansion, ventilation, PGX, and less tachypnea at peak exercise, suggesting that no restrictive ventilation impairment/limitations developed.

### Dyspnea at peak exercise

In the CHF group, PG, FVC%, IC%, EILV/TLC, and V_T_/T_Epeak_ were positively related to dyspnea score at peak exercise (Table 4, all *r* = 0.41 − 0.42, all *p* < 0.05), whereas in the ILD group, I:E and IDC were inversely related (*r* = −0.40 to −0.43, *p* ≤ 0.05). FVC%, IC%, and EILV/TLC are lung volume expansion variables, and they are similar to the common mechanisms of dyspnea in chronic interstitial and obstructive lung disorders [4]. Interestingly, PG was related to dyspnea in the patients with CHF, even though most of them presented with normal PG. V_T_/T_Epeak_ is effort-related in patients with CHF, and the higher the V_T_/T_Epeak_, the greater the dyspnea. In the ILD group, greater dyspnea was associated with more tachypnea, as T_I_ was shortened. Of note, V̇_E_/V̇O_2_ at anaerobic threshold and V̇_E_/V̇CO_2_ and oxyhemoglobin saturation at peak exercise were not related to dyspnea in the ILD group.

## Study limitations

It may be argued that the patients with CHF did not have restrictive ventilatory defects in the current study, as only three of the 27 participants with HFrEF/HFmrEF had an LVEF <30%. However, patients with severe CHF may have normal lung volumes [2], and patients with HFrEF may present with restrictive ventilatory defects alone, obstructive ventilatory defects alone, and combined obstructive and ventilatory defects as mentioned above [3]. In the current study, we excluded patients with obstructive ventilatory defects, as the study purpose was to investigate ventilatory restriction. Although the demographic data were different between the CHF and ILD groups, most of lung function and cardiopulmonary exercise data were presented as the percentage of predicted normal value or adjusted by standard variables (i.e. RV/TLC and FEV_1_/FVC), and thus most were adjusted accordingly. The restrictive ventilatory defects in the CHF patients are strongly suspected to be due to impaired ventilatory muscle strength [5,8] caused by over-expression of muscle deoxygenation [47,49]; however, they were not measured in the current study. Two-dimensional echocardiography was performed in randomly selected normal controls because the measurements were expected to be normal in the normal controls. As a result, LVEF, maximal velocity of TR, maximal pressure gradient of TR and RVSP were normal in all of the controls in the current study. In patients with ILD and suspected pulmonary hypertension, currently recommended ESC/ERS TR velocity screening thresholds have been reported to be associated with a high positive predictive value (86%) for confirming pulmonary hypertension, but to be of limited value in excluding pulmonary hypertension, with 40% of patients being misclassified as having a low probability when pulmonary hypertension was confirmed during subsequent right-heart catheterization [50]. Most of the patients with ILD in the current study had an abnormal TR velocity rather than a normal value, and the TR velocity value was higher than that in the CHF group and normal controls. Thus, right-heart catheterization could be omitted in the ILD group for simplicity and to avoid an invasive procedure in the current study. Although the pre-capillary or post-capillary type of group 2 pulmonary hypertension has been reported [19,41,42], TRV, PG and RVSP at rest were normal in the CHF group in the current study. Thus, a large scale study including patients with a more severe stage of CHF is warranted. Cigarette smoking may have confounded the study; however, it is highly prevalent in patients with CHF (Table 1) [51]. If smokers were excluded, the enrollment of participants would be very slow and would not reflect real-world conditions. Patients with CHF and heavy smoking history may develop chronic pulmonary fibrosis with emphysema (CPFE) for which HRCT is mandatory to differentiate those whose lung function involves a mixed type of ventilatory defects [52,53]. However, lung function of the CHF patients in the current study was normal and was quite different from that of CPFE patients. We excluded patients with CHF and obstructive ventilatory defects, as investigating obstructive ventilatory defects was not the purpose of this study. Lastly, defining maximal exercise according to heart rate is challenging in patients with CHF and those taking beta blockers. We believe that most of the CHF group reached maximal exercise as RER was >1.05 in the CHF group at peak exercise (1.06 ± 0.02). RER in the CHF group was similar to that in the ILD group; however, it was lower than that in the healthy group (1.06 ± 0.02 versus 1.05 ± 0.02, p = NS; versus 1.16 ± 0.02, *p* < 0.01).

## Conclusions

OCD and CAT can differentiate the functional capacity of CHF and ILD patients with similar self-estimated exercise capacity using NYHA class and mMRC score. Impaired left ventricular function was not complicated with pulmonary hypertension or altered lung function in the CHF group in this study, and thus breathing pattern and pulmonary gas exchange were normal at peak exercise. All of these factors were impaired in the patients with ILD. Exercise capacity was reduced commensurately in both groups; however, ventilatory flows were more impaired in the ILD group. The main factors affecting dyspnea at peak exercise were PG and lung expandability in the CHF group, whereas it was the shortened T_I_ in the ILD group. As the number of participants was small in this study, a larger scale study is warranted to confirm our findings.

## Supplementary Material

Supplemental Material

## Data Availability

All data generated or analyzed during this study are included in this published article and its supplementary information files.
