# Supplementary material for: Resting pulmonary function and artery pressure and cardiopulmonary exercise testing in chronic heart failure patients in Taiwan − a prospective observational cross-sectional study comparing healthy subjects and interstitial lung disease patients
Source: Ann Med. 2023 Jun 30;55(1):2228696. doi: 10.1080/07853890.2023.2228696 (PMC10316732; doi:10.1080/07853890.2023.2228696)
Supplement: Supplemental Material [file IANN_A_2228696_SM0168.pdf]

O2P series no.	group	Entry study (Y/N)	Entry date (Y/M/D)	Obtained date (Y/M/D)	Age	Birthday	Sex
N01	Norm	Y	10/27/2017	6/21/2017	41	8/16/1976	1
N02	Norm	Y	11/2/2017	12/6/2017	68	3/15/1949	1
N03	Norm	Y	11/2/2017	12/6/2017	42	3/28/1975	1
N04	Norm	Y	12/1/2017	12/1/2017	61	4/25/1956	1
N05	Norm	Y	12/8/2017	12/8/2017	66	2/17/1951	1
N06	Norm	Y	12/15/2017	12/15/2017	70	12/8/1947	1
N07	Norm	Y	1/23/2018	1/23/2018	63	6/25/1954	1
N08	Norm	Y	1/5/2018	1/5/2018	66	3/31/1951	1
N09	Norm	Y	1/12/2018	1/12/2018	76	1/13/1941	1
N10	Norm	Y	1/12/2018	1/12/2018	75	6/30/1942	1
N11	Norm	Y	1/12/2018	1/12/2018	78	9/23/1939	1
N13	Norm	Y	4/20/2018	4/20/2018	64	2/25/1954	1
N14	Norm	Y	4/20/2018	4/21/2018	63	7/9/1954	1
N15	Norm	Y	4/24/2018	4/24/2018	63	9/19/1954	1
N16	Norm	Y	5/16/2018	5/16/2018	65	3/12/1953	1
N17	Norm	Y	5/16/2018	5/16/2018	70	1/23/1948	1
N18	Norm	Y	5/15/2018	5/15/2018	59	6/13/1958	1
N19	Norm	Y	6/22/2018	6/22/2018	67	2/15/1951	1
N20	Norm	Y	7/11/2018	9/19/2018	70	1947/12/10/	1
N21	Norm	Y	7/17/2018	7/17/2018	63	8/19/1954	1
N22	Norm	Y	7/20/2018	7/20/2018	60	7/14/1958	1
N23	Norm	Y	7/17/2018	7/17/2018	66	7/24/1951	1
N24	Norm	Y	7/13/2018	7/18/2018	67	6/13/1951	1
N25	Norm	Y	7/20/2018	7/20/2018	56	1961/10/18	1
N26	Norm	Y	7/20/2018	9/5/2018	54	5/4/1964	1
N27	Norm	Y	6/14/2019	6/14/2019	54	3/26/1965	1
N28	Norm	Y	6/19/2019	6/19/2019	54	8/20/1964	1
N29	Norm	Y	6/21/2019	6/21/2019	65	10/27/1953	1
N30	Norm	Y	7/17/2019	7/17/2019	68	3/7/1951	1
N31	Norm	Y	7/12/2019	7/12/2019	52	4/18/1967	1
N32	Norm	Y	7/12/2019	7/12/2019	54	7/19/1964	1
N33	Norm	Y	8/7/2019	8/7/2019	51	6/14/1968	1
N34	Norm	Y	8/21/2019	8/21/2019	47	12/21/1971	1
F01	CHF	Y	4/18/2018	4/23/2018	63	9/15/1954	1
F02	CHF	Y	4/18/2018	4/18/2018	65	7/13/1952	1
F03	CHF	Y	5/30/2018	5/30/2018	74	3/19/1944	1
F04	CHF	Y	6/5/2018	6/5/2018	44	3/15/1974	1
F05	CHF	Y	5/8/2018	5/8/2018	53	8/4/1964	1
F06	CHF	Y	5/30/2018	5/30/2018	54	7/27/1963	1
F07	CHF	Y	5/15/2018	5/15/2018	58	11/17/1959	1

F08	CHF	Y	5/29/2018	5/29/2018	61	7/10/1956	1
F09	CHF	Y	5/16/2018	5/16/2018	38	6/28/1979	1
F10	CHF	Y	6/19/2018	6/19/2018	64	6/10/1954	1
F11	CHF	Y	6/19/2018	6/19/2018	50	3/11/1968	1
F12	CHF	Y	6/27/2018	7/18/2018	53	10/12/1964	1
F13	CHF	Y	9/11/2018	9/11/2018	58	12/3/1959	1
F14	CHF	Y	9/11/2018	9/11/2018	58	11/29/1959	1
F15	CHF	Y	9/21/2018	9/21/2018	56	1962/01/15	1
F16	CHF	Y	11/2/2018	11/2/2018	62	11/5/1955	1
F17	CHF	Y	11/21/2018	11/21/2018	63	2/6/1955	1
F18	CHF	Y	10/16/2019	10/16/2019	67	12/18/1951	1
F19	CHF	Y	1/16/2020	1/16/2020	60	7/17/1959	1
F20	CHF	Y	4/10/2020	4/10/2020	78	2/17/1942	1
F21	CHF	Y	4/17/2020	4/17/2020	52	3/23/1968	1
F22	CHF	Y	4/24/2020	4/24/2020	45	9/3/1974	1
F23	CHF	Y	5/15/2020	5/15/2020	45	2/11/1975	1
F24	CHF	Y	5/29/2020	5/29/2020	55	9/18/1964	1
F25	CHF	Y	6/5/2020	6/17/2020	66	7/17/1953	1
F26	CHF	Y	6/19/2020	7/3/2020	50	5/29/1970	1
F27	CHF	Y	6/19/2020	7/8/2020	65	10/1/1954	1
8400246	ILD	Y	2020/02/13	2020/02/13	47	12/20/1972	M
2252134	ILD	Y	2020/05/22	2020/05/22	64	1/13/1956	F
1020132	ILD	Y	2020/06/03	2020/06/03	62	3/22/1958	F
618398	ILD	Y	10/14/2020	10/14/2020	70	7/28/1950	M
8374547	ILD	Y	2020/06/12	2020/06/12	66	11/3/1954	F
2510594	ILD	Y	2020/08/07	2020/08/07	35	6/6/1985	F
445747	ILD	Y	2020/08/05	2020/08/05	65	3/30/1955	F
750374	ILD	Y	2020/09/30	2020/09/30	54	12/26/1966	F
2322749	ILD	Y	2020/10/07	2020/10/07	75	4/27/1945	M
2500348	ILD	Y	10/14/2020	10/14/2020	67	9/8/1953	M
2033949	ILD	Y	11/19/2020	11/19/2020	53	5/20/1968	F
2420221	ILD	Y	12/25/2020	12/25/2020	66	12/7/1954	F
712557	ILD	Y	2021/01/08	2021/01/08	70	5/6/1951	F
2280341	ILD	Y	2021/01/14	2021/01/14	66	6/7/1955	M
2536806	ILD	Y	5/12/2021	5/12/2021	60	3/2/1961	M
8474282	ILD	Y	8/27/2021	8/27/2021	70	9/12/1951	F
1107970	ILD	Y	9/3/2021	9/3/2021	69	6/23/1952	F
8245241	ILD	Y	9/6/2021	9/6/2021	76	2/14/1946	M
2619300	ILD	Y	8/16/2021	8/16/2021	71	3/20/1951	M
2279696	ILD	Y	8/19/2021	8/19/2021	79	6/10/1942	F
2648972	ILD	Y	9/29/2021	9/29/2021	82	7/22/1939	M
8325162	ILD	Y	9/20/2021	9/20/2021	41	2/17/1981	F
2472491	ILD	Y	5/2/2018	5/2/2018	76	8/25/1942	F

Height,c m	Weight,kg	BMI	moking, p	smk, yr	smk, P-Y	戒菸與否	戒菸幾年
172	72	24.3	0	0	0		
161	76	29.3	0	0	0		
175	79	25.7	0	0	0		
175.5	84	27.2	2	25	50	Y	2
168	56	19.8	0	0	0		
170	71	24.5	0	0	0		
162	61	23.2	0.2	5	1	Y	20
173	81	27	0	0	0		
163	75	28.2	0	0	0		
167	68	24.3	0.5	2	1	Y	
160	75	28.9	0	0	0		
163	67.8	25.5	0	0	0		
168	80	28.3	0	0	0		
169	77.8	27.2	0	0	0		
163	62	23.3	0.5	20	10	N	
159	55	21.7	0	0	0		
167	63	22.5	0	0	0		
165	64	23.5	0	0	0		
161	65	25.04	0	0	0		
163	64	24.1	0	0	0		
178	79	24.9	0	0	0		
158	52	20.8	0	0	0		
174	67	22.1	1包, 55-60	41	24.8	N	
164	75	27.9	0	0	0		
165	69	25.3	0.2	2	0.4	Y	34
163	60	22.6	0	0	0		
170	57	19.7	0	0	0		
166	61	22.1	0	0	0		
168	75	26.6	0	0	0	Y	20
168	71	25.2	0	0	0		
166	79	28.7	0	0	0		
163	56	21.1	0	0	0		
175	82	26.8	0.5	15	7.5	N	0
172	80	27	2	40	80	Y	5
168	78	27.6	3	30	90	Y	10
156	55	22.6	0.5	48	24	Y	6
167	81	29	1	15	15	Y	5
171	60	20.5	3	20	60	Y	12
165	80	29.3	1	30	30	Y	0.25
170	66	22.8	0		0		

166	77	27..9	2	20	40	Y	10
170	87	30.1	3	10	30	Y	2
172	75	25.3	0		0		
174	97	32	1.5	21	31.5	N	
168	83	29.4	1	5	5	Y	20
158	77	30.8	1	15	15	Y	8
164	70	26	0.5	55	27.5	Y	5
169	85	29.8	1.5	30	45	Y	2
172	78	26.4	2	35	70	Y	2
163	73	27.5	0		0		
172	74	25	0	0	0		
167	75	26.9	1	44	44	N	
167	55	19.72	2	37	74	Y	20
163	60.2	22.6	0	0	0	0	0
171	80	27.3	1	30	30	N	0
164	77	28.6	2	28	56	Y	2年1月
168	71	25.15	0	0	0	0	0
168	68	24.09	0.2	10	2	N	0
169	89	31.1	0.1	10	1	N	0
166	69	25.09	0.5	20	10	Y	25
171	74	25.31	1	20	20	Y	2
158	66.5	26.64	0	0	0	NA	NA
150	63	28.00	0	0	0	NA	NA
172	52	17.58	0	0	0	NA	NA
160	61	23.83	0	0	0	NA	NA
146	49.6	23.27	0	0	0	NA	NA
166	65	23.59	1	7.5	7.5	Y	1
164	59	21.94	0	0	0	NA	NA
170	58	20.07	0	0	0	NA	NA
169	61	21.36	1	50	50	N	0
156	48	19.72	0	0	0	NA	NA
158	60	24.03	0	0	0	NA	NA
165	67	24.61	2	30	60	Y	0.17
160	57	22.27	0.67	40	26.7	N	0
163	61	22.96	2.03	29	59	Y	17
153	55	23.50	0	0	0	NA	NA
150	63	28.00	0	0	0	NA	NA
164	60	22.31	0	0	0	NA	NA
161	77	29.71	0.5	2.5	1.25	Y	45
158	57	22.83	0	0	0	NA	NA
157	59	23.94	0	0	0	NA	NA
162	61	23.24	0	0	0	NA	NA
156	57	23.42	0	0	0	NA	NA

Obtained date_CBC (Y/M/D)	wbc	myelo	band	seg	lymph	mono	eos	bas
1/15/2018	5540							
12/7/2017	6980							
12/13/2017	6260							
12/1/2017	5380							
12/8/2017	3630							
12/15/2017	5700							
1/5/2018	4950			63.2	25.9	7.7	2.8	0.4
1/5/2018	6830							
1/12/2018	6400							
1/12/2018	6970							
1/12/2018	6520							
4/20/2018	7710			68.5	23	6.5	1.6	0.4
4/21/2018	7740			58.3	33.9	6.5	0.8	0.5
4/24/2018	6930			57.6	30.6	6.9	4.3	0.6
6/13/2018	9170			51.1	39.9	5.1	3.1	0.8
5/16/2018	5950			51	38.2	8.1	2.2	0.5
5/15/2018	6390			52.9	35.5	7.2	3.9	0.5
6/22/2018	4230			52.4	35	9.5	2.4	0.7
9/19/2018	4810			62.6	25.6	9.1	1.7	1
7/17/2018	5160			57.2	30.4	8.1	3.9	0.4
7/20/2018	4760			39.6	51.3	6.3	1.7	1.1
7/17/2018	6680			61.1	32.8	4.9	0.9	0.3
7/18/2018	4860			50.4	37.9	7.4	3.7	0.6
7/20/2018	8520			78.6	10.7	7.4	2.8	0.5
9/5/2018	5360			62.4	28.7	5	3.2	0.7
6/14/2019	6580			52.6	38.6	7.1	1.1	0.6
6/19/2019	5800			61.6	32.4	4.8	0.7	0.5
6/21/2019	4720			42.6	49.4	5.5	2.1	0.4
7/17/2019	8070			62.6	28.5	7.2	1	0.7
7/12/2019	4890			67.7	21.5	4.7	5.5	0.6
7/12/2019	6240			50.5	38.6	8.8	1.3	0.8
8/7/2019	7050			53.6	38.7	6.1	1	0.6
8/21/2019	6990			63.7	26	6.4	3.3	0.6
4/23/2018	7920			75.3	16.7	6.1	1	0.9
4/18/2018	7620			53.2	36.2	7.8	2.2	0.6
	未抽血							
6/8/2018	7840			63.6	21.2	6.9	7.7	0.6
5/8/2018	6240			61	30.8	6.4	1	0.8
3/26/2018	7480							
5/15/2018	7220							

5/29/2018	6260							
6/8/2018	6920							
6/9/2018	7300			72.9	18.6	5.3	2.7	0.5
6/19/2018	6930			49	41.2	7.6	1.5	0.7
7/20/2018	6310			73.8	16.8	7	2.1	0.3
9/4/2018	10790			68.4	23.8	4.6	2.8	0.4
9/5/2018	7940			59.9	23.6	8.7	0.9	0.9
9/21/2018	10720			57	31	8	4	0
11/2/2018	7960			63.2	27	4.8	4.4	0.6
11/21/2018	7670			56.5	31.3	8.7	2.6	0.9
10/16/2019	5960			74.5	20.1	4.2	0.7	0.5
1/16/2020	8100			65.1	26	7.2	1.1	0.6
2/17/2020	6650			56.5	31.4	9.6	1.7	0.8
4/24/2020	9790			63.4	25.3	5.4	5.5	0.4
5/6/2020	9140			59.3	29.4	7.8	2.7	0.8
5/22/2020	6130			63.9	30	4.4	1	0.7
6/4/2020	6460			59.2	22.4	7.1	10.7	0.6
6/17/2020	10200			59.8	30.5	6.3	2.9	0.5
7/3/2020	3800			55.4	30.3	9.5	3.7	1.1
7/8/2020	6160			64	18.5	6	10.7	0.8
2/12/2020	8560			82.4	10.0	5.7	1.4	0.5
5/22/2020	6700			62.0	28.2	4.5	4.0	1.3
6/8/2020	10010			71.4	21.4	6.5	0.4	0.3
10/12/2020	7060			75.8	13.7	9.9	0.3	0.3
6/12/2020	9800			58.7	28.2	8.5	4.0	0.6
8/7/2020	7520			77.2	17.3	4.8	0.4	0.3
7/13/2020	7270			60.6	33.0	3.4	2.6	0.4
9/30/2020	4280			54.4	37.9	5.1	1.9	0.7
10/7/2020	8430			74.8	15.2	5.5	4.0	0.5
10/14/2020	6810			57.8	21.9	12.0	7.9	0.4
12/1/2020	5960			69.7	21.7	7.3	0.8	0.5
1/25/2021	7200			71.8	20.3	7.1	0.3	0.5
1/23/2021	5980			74.9	16.4	4.7	3.2	0.8
1/14/2021	13100			61.7	28.1	6.9	2.2	1.1
5/12/2021	6430			57.1	30.8	8.2	3.3	0.6
8/27/2021	8450			72.1	20.7	5.5	1.2	0.5
9/3/2021	7800			75.1	17.1	5.6	1.8	0.4
9/10/2021	7180			70.8	18.1	8.5	2.2	0.4
9/13/2021	11710			89.0	5.9	4.4	0.4	0.3
9/16/2021	6630			49.0	37	7.1	6.3	0.6
9/20/2021	8470			59.3	31.4	7.3	1.5	0.5
9/17/2021	8030			67.9	22.4	6.5	2.5	0.7
5/2/2018	3640							

RBC 10^4	Hb	platelet 10^3	Cr	Na	K	GPT	Bil
495	14.5	292	0.79	140	4.6	49	0.5
457	13.8	203	0.9	136	4.2	19	1.1
477	13.2	234	0.96	140	3.8	44	0.5
443	13.2	214	1	139	3.7	15	0.6
454	13.5	157	0.7	141	3.5	20	1.1
504	16.6	162	1.33	138	4	22	0.8
519	15.1	189	0.91	139	4.2	38	1.4
442	14.5	182	0.98	139	4.6	40	1.2
433	14.2	258	1.08	141	4.5	20	1.1
560	13	314	0.73	136	4.7	18	0.6
502	16.6	195	0.98	135	3.6	33	0.8
475	14.6	232	1.05	139	3.5	32	0.5
517	16.3	261	0.99	140	4.4	19	0.9
476	14.5	175	0.84	138	4	19	1.6
456	15	352	0.84	138	4.5	45	0.4
460	14.5	180	0.86	135	4.2	26	0.4
458	14.6	222	0.77	138	4.1	17	0.4
466	14.5	265	0.87	140	3.9	19	1
473	15.3	220	0.89	138	4.1	21	0.5
476	14.2	266	1.29	139	4.5	52	0.6
514	15.3	264	0.91	137	3.6	37	1.1
414	12.8	245	1.25	139	3.6	25	0.6
447	14	157	1.23	137	4.1	17	0.7
522	14.8	194	1	140	3.4	29	1
488	15.9	165	0.78	135	3.6	23	1.8
507	16.6	247	0.95	136	4.1	22	0.6
491	15.8	288	0.69	137	4.1	16	0.7
435	13.5	209	1.16	142	4.4	32	1.5
512	15.9	251	1.21	138	4.4	22	0.7
505	15.2	238	1.01	141	4.6	18	1.3
531	16.5	310	0.92	144	4.1	43	1.4
479	14.8	268	0.88	137	3.8	22	0.4
594	13.3	411	1.03	140	3.9	36	0.7
506	16.4	208	1.03	133	3.8	15	1.1
537	16.4	270	1.25	139	4	46	0.8
498	15.2	266	0.9	138	3.9	47	0.7
512	15.8	248	0.83	134	5	30	0.8
496	15.1	194	1.16	139	3.2	13	0.9
531	16.2	232	1.19	140	3.9	29	0.6

460	14	287	1.49	140	5.3	24	0.7
560	17	218	0.75	137	4.1	36	1.1
477	14.9	212	0.98	139	4	37	0.4
533	16.7	171	1.38	141	3.9	31	0.5
537	16.9	278	0.99	140	3.8	71	0.7
462	14.2	208	1.26	138	3.8	33	0.7
484	15.9	325	1.18		3.5	15	1.4
479	15	431	0.91	136	3.9	26	0.8
484	15.8	198	1.24	135	4.7	23	0.5
499	16.5	243	1	140	4	26	0.5
443	14.2	147	0.97	138	4.1	22	0.7
534	16.7	265	0.9	142	4.4	50	0.4
440	14.9	259	1.42	135	4.4	15	0.2
637	11.4	332	1.38	136	3.9	31	0.7
497	15.2	267	0.96	136	4.1	20	0.8
498	15.3	278	0.99	140	4	16	0.7
461	14.3	175	1.65	139	3.7	25	0.7
519	13.5	279	1.32	138	3.6	23	0.7
510	15.3	244	1.01	138	3.5	15	0.9
382	15.6	148	1.5	136	4.5	32	1.1
517	16.0	245	1.04	141	4.0	12	0.8
514	11.4	223	0.89	134	4.4	21	0.5
466	14.1	233	0.65	138	3.0	13	0.5
389	11.6	233	0.82	139	3.5	11	0.6
450	14.8	299	0.80	137	3.8	14	0.7
463	13.5	328	0.81	136	4.0	14	0.5
433	12.9	297	0.82	137	4.1	16	0.2
370	12.0	180	0.65	140	4.5	14	0.5
475	14.0	189	0.81	140	3.9	10	1.0
399	13.6	214	0.97	135	3.7	9	1.0
486	10.8	233	0.54	136	4.2	14	0.5
446	13.7	358	0.78	142	4.2	18	0.7
375	11.7	252	1.04	133	4.2	21	0.3
506	15	348	0.67	136	4.5	13	0.5
439	13.6	199	0.77	134	4.1	23	0.8
483	13.2	211	0.6	138	3.5	23	0.7
420	12.4	238	0.99	142	3.3	54	0.6
387	12.4	148	0.93	135	3.9	28	0.5
513	15.2	237	1.13	138	3.6	26	0.7
360	10.6	169	1.97	136	5.1	10	0.7
440	14.5	211	1.04	139	4.4	20	1.4
437	14	310	0.67	137	3.9	13	0.6
360	12.3	174	0.88			25	

CRP	NT proBNP	glucose, PC		Obtained date_Q (Y/M/D)	OCD	mMRC 0-4
0.036	51	98		6/21/2017	10	0
0.058	19	115		11/2/2017	6.2	0
0.53	15	88	BNP<15月	11/2/2017	7.4	0
0.23	36	144		12/1/2017	7.4	0
0.02	90	172	crp<0.02月	12/8/2017	7.4	0
0.116	59	130		12/15/2017	8	0
0.769	42	117		1/23/2018	8	0
0.599	193	95		1/5/2018	10	0
0.12	30	107		1/12/2018	6.2	0
0.043	84	126		1/12/2018	8	0
0.101	57	101		1/12/2018	8	0
0.101	30	137		4/20/2018	8	0
0.252	18	110		4/20/2018	8	0
1.036	26	90		4/24/2018	8	0
0.093	27	101		6/13/2018	8	0
0.074	45	106		5/16/2018	8	0
0.045	44	93		5/15/2018	8	0
0.048	22	80		6/22/2018	8	0
0.038	39	98		9/19/2018	9	0
0.109	107	92		7/17/2018	8	0
0.038	41	86		7/20/2018	8	0
0.043	24	131		7/17/2018	8	0
0.044	105	84		7/18/2018	8	0
0.187	18	146		7/20/2018	10	0
0.051	33	131		9/5/2018	8	0
0.082	30	107		6/14/2019	10	0
0.201	55	91		6/19/2019	8	0
0.025	44	87		6/21/2019	8	0
0.043	38	104		7/17/2019	8	0
0.037	23	131		7/12/2019	10	0
0.107	15	108		7/12/2019	10	0
0.02	20	90		8/7/2019	10	0
0.02	15	98		8/21/2019	8	0
0.155	913	107		4/18/2018	6.9	0
0.029	492	100		4/18/2018	8	0
				5/30/2018	7.1	0
0.347	71	120		6/5/2018	8	0
0.029	117	402		5/8/2018	7.1	1
0.125	228	89		5/30/2018	10	0
0.025	32	106		5/15/2018	6.2	0

1.415	499	155		5/29/2018	7.1	0
0.082	126	204		5/16/2018	8	1
0.212	88	132		6/19/2018	7.5	0
-	-	89		6/19/2018	9	1
0.29	537	121		7/20/2018	7.5	1
0.268	113	152		9/4/2018	8	0
0.513		121		9/5/2018	7.1	0
0.114	130	89		9/21/2018	8	1
0.117	981	262		11/2/2018	8	0
0.174	49	113		11/21/2018	8	0
0.021	495	145		10/16/2019	8	0
0.058	630	105		1/16/2020	7.5	2
0.253	986	108		4/10/2020	5.3	1
0.896	308	131		4/24/2020	7.1	1
0.141	69	126		5/6/2020	8.5	0
0.104	172	136		5/22/2020	8	0
0.02	344	150		6/4/2020	8	0
0.197	554	114		6/17/2020	6.2	2
0.121	344	136		7/3/2020	7.1	0
0.045	320	231		7/8/2020	6.2	2
0.150	125			2020/02/13	6.2	1
0.089	183	104		2020/05/22	7.5	1
0.467	16	142		2020/06/03	5.3	1
0.042	112			10/14/2020	6.2	2
0.089		77		2020/06/12	7.1	1
0.029	16	94		2020/08/07	7.1	1
0.404	83	108		2020/08/05	8.0	1
0.031	22	104		2020/09/30	7.9	0
0.129	60	124		2020/10/07	8.0	0
0.323	178	102		10/14/2020	6.2	1
0.042	67	93		11/19/2020	8.0	0
0.53	466	84		12/25/2020	7.1	0
0.111	515	114		2021/01/08	8.0	0
0.639	73			2021/01/14	8.0	0
0.551	53			5/12/2021	6.2	2
0.511	343	119		8/27/2021	6.2	0
0.265	26	107		9/3/2021	6.2	1
0.362	37	114		9/6/2021	8	0
0.369	805	135		8/16/2021	5.3	3
0.309	1215	96		8/19/2021	5.3	1
1.143	165	89		9/29/2021	6.2	0
0.119	58	81		9/20/2021	8	0
0.444					5.7	

Borg @rest	cat1	cat2	cat3	cat4	cat5	cat6	cat7	cat8
0	0	0	0	0	0	0	0	0
0	0	0	0	1	0	0	0	0
0	0	0	0	0	0	0	0	0
0	0	1	2	0	0	0	1	0
0	0	0	1	0	0	0	0	0
0	0	0	0	0	0	0	0	0
0	0	0	0	0	0	0	0	0
0	0	0	0	0	0	0	0	0
0	0	0	0	0	0	0	0	0
0	0	0	0	0	0	0	0	0
0	0	0	0	0	0	0	0	0
0	0	0	0	0	0	0	0	0
0	0	0	0	0	0	0	0	0
0	0	0	1	0	0	0	0	0
1	0	0	0	0	0	0	0	0
0	0	0	0	0	0	0	0	0
0	0	0	0	0	0	0	0	0
0	1	0	0	0	0	0	0	0
0	0	0	0	0	0	0	0	0
0	0	0	0	0	0	0	0	0
0	0	0	0	0	0	0	0	0
0	0	0	0	0	0	0	0	0
0	0	0	0	0	0	0	0	0
0	0	0	0	0	0	0	0	0
0	0	0	0	0	0	0	0	0
0	0	0	0	0	0	0	0	0
0	0	0	0	0	0	0	0	1
0	0	1	0	0	0	0	0	0
0	0	0	0	0	0	0	0	0
0	0	0	0	0	0	0	0	0
0	0	0	1	0	0	0	0	1
0	1	0	1	0	0	0	0	0
0	0	1	0	1	0	0	0	1
0	0	2	2	0	1	0	0	0
0	1	1	0	0	0	1	1	1
0	0	0	0	0	0	0	0	0
0.5	1	0	1	2	0	0	0	1
0	0	0	0	2	0	0	0	0
0	0	0	0	0	0	0	0	0
0	0	0	0	1	0	0	0	0

0.5	0	0	1	0	0	0	0	0
1	0		4	0	0	0	1	1
1	2	2	1	1	1	1	1	2
1	1	2	1	0	0	0	1	1
0	0	0	1	0	3	4	0	4
0	0	0	1	0	0	0	0	0
1	0	0	1	1	0	0	1	0
0.5	0	1	0	0	1	0	1	0
0	0	0	0	0	0	0	1	1
0.5	0	0	0	0	0	0	0	0
0	0	1	0	0	0	0	0	1
0.5	0	3	1	3	0	0	1	1
0	0	1	0	0	0	0	0	1
0	2	0	1	2	0	0	0	0
0	0	0	1	1	0	0	0	0
0.5	0	0	0	0	0	0	0	0
1	1	1	0	2	0	0	1	1
0.5	3	3	0	3	0	0	0	0
0.5	0	0	0	0	0	0	0	0
1	1	0	0	1	0	0	0	0
0	1	1	0	1	0	0	0	3
0.5	1	1	0	0	0	0	0	0
2	1	1	3	0	0	0	2	3
0	1	1	4	3	3	3	2	5
0	1	1	0	0	0	1	0	2
0.5	3	0	0	3	0	0	0	2
3	1	0	1	2	2	4	3	3
2	3	0	0	2	0	0	4	3
0	3	1	0	0	0	0	0	1
0	1	1	0	0	0	0	0	2
0.5	1	1	1	3	0	0	1	1
0	0	0	0	0	0	0	0	0
0	1	1	1	2	0	0	0	3
0	0	1	0	0	0	0	0	1
0	3	5	0	0	0	0	0	2
0	3	2	0	1	0	0	1	0
0	0	0	1	0	0	3	2	2
0.5	4	3	1	3	0	0	0	0
0.5	3	1	3	5	0	2	0	0
0.5	4	5	1	5	5	3	0	4
0	2	1	0	2	0	0	0	1
0	0	0	0	0	0	0	0	1

NYHAFc	cat sum	DM	HTN	HD	lung dis	brochitis	asthma	hronic rena
1	0	0	0	0	0	0	0	0
1	1	0	0	0	0	0	0	0
1	0	0	0	0	0	0	0	0
1	4	0	1	1	0	0	0	0
1	1	0	0	0	0	0	0	0
1	0	0	0	0	0	0	0	0
1	0	0	0	0	0	0	0	0
1	0	0	0	0	0	0	0	0
1	0	0	0	0	0	0	0	0
1	0	0	0	0	0	0	0	0
1	0	0	1	1	0	0	0	0
1	0	0	0	0	0	0	0	0
1	0	0	0	0	0	0	0	0
1	1	0	0	0	0	0	0	0
-	0	0	0	0	0	0	0	0
1	0	0	0	0	0	0	0	0
1	0	0	0	0	0	0	0	0
1	1	0	0	0	0	0	0	0
1	0	0	0	0	0	0	0	0
1	0	0	0	0	0	0	0	0
1	0	0	0	0	0	0	0	0
1	0	0	0	0	0	0	0	0
1	0	0	0	0	0	0	0	0
1	0	0	0	0	0	0	0	0
1	0	0	0	0	0	0	0	0
1	0	0	0	0	0	0	0	0
1	1	0	0	0	0	0	0	0
1	1	0	0	0	0	0	0	0
1	0	0	1	0	0	0	0	0
1	0	0	0	0	0	0	0	0
1	2	0	0	0	0	0	0	0
1	2	0	0	0	0	0	0	0
1	3	0	0	0	0	0	0	0
2	5	0	0	1	0	0	0	0
1	5	0	0	1	0	0	0	0
1	0	1	0	1	0	0	0	0
2	5	0	1	1	0	0	0	0
1	2	1	1	1	0	0	0	0
1	0	0	1	1	0	0	0	0
1	1	0	0	0	0	0	0	0

1	1	1	0	1	0	0	0	0
2	6	1	0	1	0	0	0	0
2	11	0	0	10	0	0	0	0
2	6	0	1	1	0	0	0	0
2	12	0	1	1	0	0	0	0
2	1		1	1	0	0	0	0
2	3	0	1	1	0	0	0	0
2	3	0	0	1	0	0	0	0
1	2	0	1	1	0	0	0	0
1	0	0	0	1	0	0	0	0
1	2	0	1	1	0	0	0	0
2	9	0	1	1	0	0	0	0
1	2	0	0	1	0	0	0	0
2	5	0	0	1	0	0	0	0
1	2	1	0	1	0	0	0	0
1	0	0	1	1	0	0	0	0
1	6	1	1	1	0	0	0	0
3	12	0	1	1	0	0	0	0
1	0	0	0	1	0	0	0	0
2	2	1	1	1	0	0	0	0
2	6	0	0	0	0	0	0	0
2	2	0	0	0	0	0	0	0
2	10	0	1	1	1	1	1	0
1	22	0	0	0	0	0	0	0
2	5	0	0	1	0	0	0	0
2	8	0	0	0	1	0	0	0
2	16	0	0	0	1	0	0	0
1	12	0	0	0	0	0	0	0
1	5	0	0	0	0	0	0	0
2	4	0	0	0	0	0	0	0
1	8	0	0	0	0	0	0	0
1	0	0	0	0	0	0	0	0
1	8	0	1	0	0	0	0	0
1	2	1	0	0	0	0	0	0
3	10	0	0	0	0	0	0	0
2	7	0	1	1	0	0	0	0
1	8	0	1	0	1	0	0	0
1	11	0	0	1	1	0	0	0
3	14	1	1	1	1	1	1	0
2	27	0	0	1	1	0	1	0
1	6	0	1	0	1	0	0	0
1	1	0	0	0	0	0	0	0
		0	1	0	1	0	1	0

uto immun	cancer	others	leisure activity ,1,2,3	leisur ,0,1,2,3,4	Obtained date_PFT (Y/M/D)	FVC
0	0	0	2	2	10/27/2017	4.2
0	0	0	2	2	11/2/2017	2.73
0	0	0	3	1	11/2/2017	4.64
0	1	0	2	1	12/1/2017	4.25
0	0	0	1	4	12/8/2017	3.12
0	0	0	2	3	12/15/2017	3.56
0	0	0	2	2	1/5/2018	3.42
0	0	0	2	2	1/5/2018	3.06
0	0	0	1	3	1/12/2018	3.67
0	0	1	1	3	1/12/2018	3.31
0	0	0	1	3	1/12/2018	2.51
0	0	0	1	4	4/20/2018	2.96
0	0	0	1	4	4/20/2018	2.96
0	0	0	2	4	4/24/2018	4.39
0	0	0	1	4	6/13/2018	3.83
0	0	0	2	4	5/16/2018	3.35
0	0	0	3	0	5/15/2018	3.16
0	0	0	2	2	6/22/2018	3.2
0	0	抗焦慮藥	2	4	9/19/2018	3.57
0	0	0	1	4	7/17/2018	3.63
0	0	0	3	0	7/20/2018	4.51
0	0	0	3	0	7/17/2018	2.98
0	0	0	1	4	7/18/2018	4.11
0	0	0	1	4	7/20/2018	3.87
0	0	0	1	4	9/5/2018	4.77
0	0	0	1	3	6/14/2019	3.59
0	0	0	2	1	6/19/2019	4.66
0	0	0	2	2	6/21/2019	3.88
0	0	吃了一個	1	3	7/17/2019	3.14
0	0	0	1	4	7/12/2019	4.92
0	0	0	1	3	7/12/2019	3.96
0	0	心跳慢	1	3	8/7/2019	3.84
0	0	0	2	1	8/21/2019	5.2
0	0	0	1	4	4/18/2018	3.69
0	1	0	2	1	3/26/2018	2.77
0	0	0	1	4	5/30/2018	2.11
0	0	0	3	0	6/5/2018	3.99
0	0	0	1	4	5/8/2018	4.26
0	0	0	1	4	5/30/2018	3.96
0	0	0	1	4	4/2/2018	3.63

0	0	0	1	4	3/2/2018	2.67
0	0	0	3	0	5/1/2018	4.01
0	0	0	3	0	6/19/2018	3.89
0	0	0	1	3	6/19/2018	4.59
0	0	1	2	1	7/20/2018	5.01
0	0	0	3	1	9/4/2018	3.38
0	0	周邊動脈	1	4	9/5/2018	3.46
0	0	左前降枝	1	4	9/21/2018	3.86
0	0	0	1	2	10/8/2018	3.34
0	0	0	1	4	10/31/2018	3.12
0	0	0	1	2	10/16/2019	3.03
0	0	之前有過	2	1	1/16/2020	2.74
0	0	0	1	3	4/8/2020	2.77
0	0	0	2	1	4/24/2020	3.85
0	0	0	1	3	2/17/2020	4.95
0	0	痛風	2	1	5/22/2020	3.39
0	0	三高	1	4	5/29/2020	4.19
0	0	0	1	4	6/17/2020	2.58
0	0	0	1	2	7/3/2020	3.59
0	大腸癌20	0	3	1	7/8/2020	2.93
1	0	0	3	1	2/13/2020	3.67
1	0	0	2	1	5/22/2020	1.41
0	0	眠藥,焦慮	3	1	3/16/2020	1.57
1	0	0	3	1	10/14/2020	1.79
1	0	0	3	1	6/12/2020	1.40
1	0	0	3	1	8/7/2020	1.28
1	0	0	1	2	8/18/2020	1.66
1	0	0	1	2	9/30/2020	1.98
1	0	0	1	4	10/7/2020	3.60
1	0	0	2	1	3/27/2019	2.63
1	0	0	1	2	9/1/2020	1.89
1	0	0	1	4	2020/12/25	1.07
1	0	下,胃食道逆	3	1	2021/01/08	2.54
1	0	0	2	2	12/14/2020	2.98
0	1	0	2	1	5/12/2021	2.14
1	0	0	1	3	8/27/2021	1.42
1	0	0	2	1	6/3/2021	1.24
0	0	0	1	4	9/10/2021	2.28
1	0	0	1	3	9/13/2021	1.24
1	1	0	2	2	9/16/2021	1.93
0	0	0	1	4	9/3/2021	1.48
1	0	0	1	2	11/3/2021	2.17
1	0				2/19/2019	1.15

FEV 1	FEV 1 / FVC	MMEF 75/25	PEF	IC forced	FVC_Post	FEV 1_Post
3.39	80.77	3.45	7.34	2.45		
2.12	77.38	2.08	7.47	2.01		
3.79	81.71	3.71	9.84	3.2		
3.65	86.06	5.37	10.14	3.77		
2.73	87.76	3.75	10.2	2.43		
2.82	79.15	2.93	10.42	2.94		
2.84	83.04	3.26	7.42	2.46		
2.41	78.77	2.17	6.5	2.61		
2.97	80.93	2.99	6.09	3.33		
2.46	74.2	1.79	7.18	2.69		
2.14	85.29	2.72	6.74	2.56		
2.58	86.99	4.7	6.85	2.64		
2.45	82.9	2.81	7.52	2.18		
3.28	74.66	2.47	9.32	3.48		
2.68	69.94	1.69	3.23	3.21		
2.44	73	1.72	5.83	2.02		
2.53	80.18	2.39	6.58	2.56		
2.56	79.94	2.73	4.64	2.82		
2.89	80.97	3.08	6.31	3.24		
2.69	73.88	1.97	7.51	2.99		
3	66.63	2.21	8.31	2.78		
2.19	73.41	1.54	6.09	2.44		
2.93	71.24	1.84	7.82	3.13		
3.22	83.17	3.71	9.54	3.17		
3.82	80.2	3.65	10.32	3.35		
2.77	77.18	2.31	8.44	2.13		
3.64	78.01	3.03	11.16	3.19		
2.96	76.28	2.44	6.84	2.99		
2.74	87.31	3.82	7.79	1.95		
4.18	84.87	4.18	10.05	3.56		
3.22	81.3	3.63	8.85	3.43		
3.54	92.3	5.61	10.1	2.3		
4.42	84.91	4.95	10.78	3.83		
3.08	83.59	3.71	6.89	2.78		
2.21	79.73	1.91	5.71	2.16	2.88	2.26
1.55	73.22	1.1	4.08	1.83		
3.3	82.69	3.55	7.9	3.44		
3.17	74.44	2.33	8.79	2.74		
3.11	78.47	3.28	6.44	3.25		
3.07	84.53	3.83	5.73	3.27	3.84	3.2

2.18	81.62	2.24	3.57	2.01	2.69	2.07
3.41	84.84	4.17	7.19	3.04	3.9	3.44
3.05	78.5	2.78	7.59	2.6	3.73	2.88
3.85	83.81	4.71	8.27	3.99		
4.12	82.26	4.01	7.93	4.21	5.02	4.11
2.76	81.65	2.91	5.76	2.86		
2.76	79.61	2.68	5.95	2.75		
3.01	77.91	3.08	7.8	3.12	4.01	3.25
2.59	77.57	2.18	7.71	2.78	3.31	2.63
2.56	82.02	3.77	6.09	2.83	2.91	2.46
2.59	85.27	3.46	8.95	2.47		
2.25	82.22	2.94	4.54	1.82	2.46	2.07
2.41	87.19	1.75	7.83	1.99		
3.45	89.65	4.63	8.21	2.7		
4.14	83.62	4.75	10.22	4.06		
2.84	83.75	3.37	5.31	3.1		
3.19	76.14	2.56	8.28	3.36		
1.95	75.39	1.57	4.07	2.06		
2.99	83.25	3.76	7.23	2.8		
2.45	83.53	3.26	7.66	2.16	2.83	2.46
3.16	86.17	3.50	8.65	2.52	3.78	3.17
1.34	95.12	2.32	2.97	1.53	-	-
1.35	86.45	2.19	5.29	1.4	1.48	1.28
1.76	98.43	3.70	4.49	1.07	-	-
1.28	91.72	2.70	4.71	1.08	-	-
1.24	96.98	2.23	2.71	0.92	-	-
1.50	90.08	2.73	5.78	1.15	1.58	1.50
1.77	89.13	2.52	4.64	1.36	-	-
2.80	77.93	2.60	5.82	1.66	-	-
2	76.19	1.6	6.02	1.72	-	-
1.62	85.45	2.06	4.3	1.27	1.84	1.51
0.94	87.85	0.93	1.98	0.84	1.27	0.99
1.88	74.06	1.46	3.58	1.67	2.54	1.92
2.25	75.65	1.78	5.35	2.18	3	2.22
1.96	91.33	3.99	5.37	1.82	2.16	1.93
1.16	81.73	0.88	3.62	1.15	-	-
1.01	81.61	1	3.34	1.21	1.31	1.01
1.87	81.98	2.05	3.18	1.76	-	-
1.15	92.93	1.67	4.32	1.06	-	-
1.64	84.95	2.09	3.83	1.59	-	-
1.37	92.66	2.54	3.53	1.42	1.51	1.3
1.71	78.53	1.56	4.95	1.66	-	-
0.95	83.22	1.08	4.75	0.64	1.16	0.97

FEV 1 / FVC_Post	MMEF 75/25_Post	PEF_Post	IC forced_Post	FVC%
				93
				86
				100
				102
				86
				98
				102
				78
				119
				100
				88
				88
				80
				117
				114
				112
				84
				94
				115
				107
				104
				99
				104
				106
				127
				99
				115
				109
				88
				123
				104
				102
				115
				94
78.41	1.9	6.24		76
				78
				96
				103
				105
83.2	3.31	6.72		92

76.72	1.64	5.21		74
88.19	4.31	8.65		90
77.19	2.33	7.27		96
				105
81.79	4.18	8.04		127
				104
				96
80.98	3.26	6.95		98
79.53	2.33	7.2		84
84.65	3.08	5.75		92
				79
83.89	2.42	5.52		74
				85
				104
				114
				86
				107
				91
				88
86.74	4	6.3		83
83.95	3.62	9.21		86
-	-	-		58
86.73	2.11	5.09		72
-	-	-		48
-	-	-		56
-	-	-		48
94.62	2.92	6.01		60
-	-	-		67
-	-	-		103
-	-	-		71
81.93	1.5	4.52		71
77.63	0.86	1.91		44
75.45	1.46	3.32		97
73.86	1.63	4.94		93
89.3	3.52	2.18		61
-	-	-		68
77.4	0.78	4.14		63
-	-	-		72
-	-	-		39
-	-	-		94
85.94	1.52	6.29		57
-	-	-		67
83.55	1.14	4.06		56

FEV 1%	FEV 1 / FVC%	MMEF 75/25%	PEF%	IC forced%	FVC%Post
91		81	82	74	
86		72	105	85	
99		87	108	93	
111		154	122	118	
97		120	134	89	
101		98	138	106	
107		104	100	99	
79		68	82	87	
128		115	88	143	
98		66	100	105	
100		111	102	119	
97		151	93	105	
84		87	97	78	
111		75	119	123	
102		55	89	129	
105		62	84	91	
85		70	84	92	
96		90	63	110	
120		109	90	140	
100		62	101	118	
88		62	98	83	
93		53	87	110	
96		58	98	103	
109		107	122	118	
126		102	130	121	
94		65	108	80	
112		82	135	106	
106		78	90	112	
99		126	103	72	
129		112	122	121	
105		101	110	122	
116		153	126	84	
120		121	121	113	
100		112	86	93	
77		60	74	78	79
75		43	62	91	
97		88	93	114	
95		62	105	89	
102		92	81	117	
98		109	71	111	97

76		68	46	74	74
92		96	80	93	87
94		71	91		96
109		120	95	122	
129		109	97	144	127
105		89	78	122	
96		79	77	103	
95		86	96	106	102
83		65	96	93	84
95		120	82	112	85
87		109	114	84	
76		88	58	65	66
99		68	111	79	
115		128	104	100	
116		116	117	127	
87		85	64	109	
102		71	102	116	
82		56	66	92	
90		98	86	93	
89		104	101	81	80
90		88	100	79	88
66		85	52	84	-
75		80	100	92	68
61		122	58	37	-
61		99	82	56	-
54		63	46	58	-
64		98	95	53	57
70		80	74	64	-
106		94	79	61	-
69		50	78	61	-
71		66	73	68	69
46		35	35	46	53
86		55	56	80	97
90		59	75	12	94
70		122	71	71	62
68		36	69	73	-
62		40	65	83	66
78		77	45	73	-
47		59	61	45	-
98		95	74	94	-
73		114	56	73	59
61		44	75	75	-
57		47	91	39	57

FEV 1%Post	FEV 1 / FVC%Post	MMEF 75/25%Post	PEF%Post	IC forced%Post
79		60	112	
102		94	83	

72		50	67	
93		99	97	
94		71	91	
128		114	98	
103		91	86	
85		69	89	
92		98	77	
70		72	71	
89		128	84	
91		91	107	
-		-	-	
71		78	96	
-		-	-	
-		-	-	
-		-	-	
64		105	99	
-		-	-	
-		-	-	
-		-	-	
67		48	77	
49		32	34	
88		55	56	
89		54	69	
69		107	60	
-		-	-	
62		32	81	
-		-	-	
-		-	-	
-		-	-	
69		68	100	
-		-	-	
58		50	78	

MVV pred	MVV dir	MVV % pred	TLC	RV	RV/ TLC	FRCpleth	TLC%
133.84	153.73	114.9	5.84	1.64	28.16	3.39	88
98.69	84	85.1	4.89	2.16	44.13	2.88	85
136.6	128.65	94.2	6.56	1.92	29.31	3.35	95
121.1	128.18	105.8	7.18	2.93	40.83	3.41	103
108.67	149.35	137.4	5.76	2.65	45.93	3.33	91
107.79	108.34	100.5	6.45	2.86	44.35	3.52	99
103.96	87.81	84.5	4.88	1.46	29.9	2.42	83
114.63	77.25	67.4	6.08	3.02	49.7	3.48	90
94.54	121.67	128.7	6.87	3.2	46.61	3.53	116
100.13	107.59	107.5	6.41	3.1	48.31	3.72	102
89.33	91.26	102.2	4.86	2.24	46.04	2.3	85
104.34	94.53	90.6	4.68	1.72	36.78	2.04	79
111.12	96.41	86.8	4.84	1.88	38.87	2.66	76
112.31	119.75	106.6	6.81	2.42	35.52	3.33	106
103.52	106	102.4	6.96	3.13	44.94	3.75	117
94.67	84.69	89.5	5.5	2.15	39.11	3.48	98
113.19	105.98	93.6	5.93	2.78	46.79	3.37	95
104.27	106.85	102.5	5.39	2.19	40.58	2.57	88
97.05	110.12	113.5	5.88	2.31	39.24	2.64	102
105.15	92.98	88.4	5.76	2.12	36.88	2.77	97
125.49	109.97	87.6	6.7	2.19	32.7	3.91	94
96.74	86.64	89.6	5.13	2.15	41.98	2.69	93
115.01	108.52	94.4	7.18	3.06	42.69	4.05	105
112.06	131.78	117.6	5.49	1.62	29.54	2.32	91
140.74	114.88	81.6	6.65	1.88	28.32	3.3	109
112.5	115.25	102.4	5.85	2.26	38.64	3.72	98
120.85	155.96	129.1	7.21	2.54	35.26	4.01	111
107.1	105.46	98.5	6.15	2.27	36.93	3.16	99
107.04	102.72	96.0	5.46	2.31	42.34	3.51	86
120.09	125.37	104.4	7.19	2.18	30.3	3.63	113
116.07	142.52	122.8	5.99	2	33.37	2.56	97
114.94	119.53	104.0	6.52	2.68	41.15	4.22	109
132.52	167.75	126.6	7.53	2.29	30.49	3.69	109
115.98	108	93.1	6.13	2.45	39.88	3.36	92
109.48	88.67	81.0	5.15	2.3	44.71	2.99	81
87.82	55.44	63.1	4.94	2.83	57.28	3.12	92
125.43	114.13	91.0	6.14	2.15	35.02	2.7	98
122.86	137.99	112.3	6.22	1.94	31.14	3.48	94
114.88	115.51	100.5	5.24	1.14	21.84	1.99	86
117.58	126.89	107.9	6.06	2.27	37.46	2.79	93

110.36	92.87	84.2	4.8	2.12	44.25	2.78	78
133.9	147.36	110.1	5.74	1.73	30.1	2.7	88
115.07	104.46	90.8	6.32	2.43	38.48	3.72	95
128.88	143.34	111.2	6.81	2.21	32.53	2.82	100
120.47	102.36	84.96721175	7.7	2.65	34.44	3.48	121
103.27	90.98	88.1	5.32	1.95	36.57	2.46	96
110.42	102.1	92.5	5.63	2.17	38.5	2.88	93
118.02	112.86	95.6	6.24	2.38	38.08	3.12	97
116.7	94.17	80.7	6.03	2.69	44.58	3.24	90
105.15	103.84	98.8	5.43	2.31	42.53	2.6	91
112.62	79.51	70.6	5.08	2.05	40.33	2.61	76
112.37	71.35	63.5	4.5	1.76	39.16	2.68	72
97.68	89.2	91.3	5.51	2.27	41.19	3.52	88
114.13	134.78	118.1	6.5	2.65	40.71	3.8	109
129.38	148.05	114.4	6.91	1.96	28.4	2.85	105
121.63	69.7	57.3	5.43	2.03	37.42	2.32	90
117.64	117.82	100.2	6.95	2.76	39.69	3.59	110
108.67	74.94	69.0	5.67	3.09	54.51	3.61	107
122.92	100.17	81.5	5.88	2.28	38.84	3.07	91
107.1	87.9	82.1	5.52	2.59	46.97	3.36	89
127.75	133.08	104.2	5.77	2.11	36.47	3.25	88
84.48	58.47	69.2	3.63	2.21	60.84	2.10	78
			2.74	1.18	42.99	1.35	67
110.18	83.31	75.6	4.18	2.39	57.16	3.11	63
85.49	52.87	61.8	2.89	1.49	51.52	1.82	61
94.23	63.24	67.1	3.16	1.83	57.78	2.24	82
90.54	61.49	67.9	3.21	1.55	48.21	2.06	62
96.23	67.75	70.4	4.17	2.19	52.49	2.81	84
103.71	107.66	103.8	5.84	2.24	38.39	4.18	90
110.68	77.86	70.3	4.1	1.47	35.92	2.38	64
91.02	60.93	66.9	3.83	1.94	50.64	2.56	85
83.11	40.1	48.2	4.02	2.95	73.5	3.18	87
86.96	71.24	81.9	5.53	2.99	54.01	3.86	108
99.94	74.62	74.7	5.53	2.55	46.01	5.25	97
91.44	88.99	97.3	3.77	1.63	43.16	1.95	63
76.84	39.06	50.8	2.8	1.26	44.98	1.65	65
73.56	44.83	60.9	3.25	2	61.67	2.04	79
95.36	90.09	94.5	4.23	1.95	46.02	2.46	70
97.05	43.88	45.2	3.64	2.32	63.69	2.58	62
74.21	53.23	71.7	3.85	1.93	49.99	2.26	83
82.49	65.13	79.0	3.48	1.93	55.37	2.06	64
104.3	76.5	73.3	4.3	2.13	49.49	2.64	88
72.52	44.25	61.0	2.28	1.13	49.71	1.64	51

RV%pred	RV/ TLC%	FRCpleth%	DLCO SB	DLCO/VA	VA	DLCO SB%
85	94	103	25.73	4.66	5.52	100
91	109	88	18.19	3.6	5.05	98
97	97	99	25.76	4.36	5.91	96
122	108	96	20.6	3.34	6.17	89
109	116	97	16.87	3.13	5.39	103
113	107	100	18.53	3.29	5.64	101
64	78	74	19.71	5.14	3.84	114
121	125	98	22.72	4.41	5.15	107
124	107	104	18.24	3.11	5.87	109
119	112	107	20.66	3.81	5.42	127
87	104	69	20.77	4.76	4.36	131
74	94	62	14.74	3.78	3.9	80
80	101	78	14.44	3.66	3.95	68
102	92	97	18.83	3.04	6.19	90
134	114	112	16.26	3.7	5.29	95
90	95	107	17.2	3.68	4.68	121
123	127	101	19.45	4.02	4.84	101
91	101	76	20.87	4.87	4.29	121
95	95	80	21.4	4.03	5.31	132
93	96	84	18.6	3.12	5.97	104
90	88	108	20.83	4.08	5.1	91
93	105	84	14.05	3.09	4.54	98
121	106	113	18.44	3.04	6.05	98
75	83	71	20.65	3.74	5.52	96
89	81	101	25.39	4.01	6.33	120
108	110	116	22.75	4.59	4.95	117
116	101	119	27.12	4.54	5.97	138
95	94	93	18.17	3.96	4.59	105
94	105	102	19.31	4.11	4.69	100
103	89	110	26.48	4.02	6.58	118
94	95	78	27.23	5.18	5.25	119
132	122	132	22.63	3.95	5.73	116
109	94	108	26.69	4.11	6.5	102
102	103	96	18.27	3.72	4.91	84
96	114	87	20.41	3.7	6.19	92
116	134	97	15.83	4.37	3.62	122
112	113	84	19.07	3.79	5.03	74
89	90	103	23.17	3.94	5.88	113
54	62	61	19.01	3.54	5.37	83
100	102	82	20.33	3.52	5.78	100

93	117	83	17.72	4.64	3.82	87
94	105	84	23.03	4.7	4.9	81
100	99	106	18.27	3.72	4.91	84
103	97	82	21.08	3.68	5.73	76
124	99	105	21.47	3.63	5.92	89
92	100	79	19.04	4.1	4.64	92
99	105	88	17.39	3.96	4.39	86
107	106	93	22.46	4.31	5.21	95
113	117	93	19.66	3.9	5.04	91
101	110	79	14.82	4.3	3.45	76
82	101	74	21.48	4.5	4.77	108
77	105	80	21.3	5.31	4.01	101
85	93	100	12.43	2.64	4.7	94
129	119	119	20.66	3.89	5.32	104
98	90	86	24.51	4.01	6.11	94
106	119	74	21.54	5.77	3.74	88
127	112	108	18.27	3.21	5.69	84
147	132	128	13.58	3.56	3.82	67
110	116	93	17.84	4.11	4.34	69
109	119	99	14.81	3.83	3.87	79
103	113	97	16.95	3.76	4.51	69
117	149	81	9.47	3.28	2.89	50
70	108	56	11.27	4.82	2.34	63
93	139	87	7.77	1.96	3.97	52
77	125	69	4.25	1.85	2.30	23
152	187	97	5.16	2.77	1.87	29
76	117	74	6.42	2.86	2.24	32
122	142	104	10.83	3.58	3.03	55
85	89	117	11.62	2.65	4.38	78
61	91	69	5.78	1.73	3.34	33
117	138	101	9.25	3.29	2.81	55
155	179	122	-	-	-	-
143	127	140	10.5	2.58	4.06	53
111	117	162	4.76	1.23	3.87	30
73	116	60	8.75	2.83	2.99	48
67	106	66	7.53	2.96	2.54	48
111	147	84	5.11	2.41	2.12	30
76	107	72	14.78	4.13	3.58	102
95	154	77	3.78	1.61	2.35	21
91	109	86	5.87	2.2	2.67	38
73	121	62	5.63	2.08	2.71	49
135	152	99	13.71	4.37	3.14	64
56	111	64	3.1	1.89	1.64	20

DLCO/VA%	VA%	Obtained date_CPET (Y/M/D)	Time	Load-rest	VO2/kg-rest	V'O2-rest
109	85	5/23/2018	3:00	0	5.2	372
99	90	11/2/2017	2:58	0	2.3	177
102	88	11/2/2017	2:58	0	3.5	262
88	91	12/1/2017	2:57	0	2.8	236
85	87	12/8/2017	2:57	0	4.6	260
92	89	12/29/2017	2:59	0	4.9	345
137	67	1/23/2018	2:59	0	4.5	274
120	78	1/5/2018	2:55	0	4.2	337
91	101	1/12/2018	3:00	0	3.7	280
110	89	1/12/2018	2:58	0	4.7	317
141	79	1/12/2018	2:57	0	4.1	308
101	67	4/20/2018	2:59	0	2.7	178
98	64	4/20/2018	2:56	0	4.6	365
81	99	4/24/2018	2:59	0	3.2	246
83	91	5/16/2018	2:58	0	5.1	315
103	85	5/16/2018	2:58	0	4.9	269
105	79	5/15/2018	2:56	0	6.4	405
133	72	6/22/2018	2:59	0	6.1	391
112	94	9/19/2018	2:59	0	1.9	126
83	103	7/17/2018	2:59	0	5	318
107	73	7/20/2018	3:00	0	2.7	211
85	84	7/17/2018	2:59	0	4.5	232
83	91	7/18/2018	2:54	0	1.1	70
95	94	7/20/2018	2:59	0	3.9	291
101	106	7/20/2018	2:54	0	5.1	352
116	85	6/14/2019	2:59	0	4	241
114	94	6/19/2019	2:58	0	6.6	377
107	76	6/21/2019	2:55	0	6.1	370
113	76	7/17/2019	3:00	0	3.7	274
100	106	7/12/2019	2:56	0	2.7	191
131	87	7/12/2019	2:30	0	4.1	322
98	98	8/7/2019	2:57	0	5.3	294
99	96	8/21/2019	2:45	0	5.9	482
99	75	4/18/2018	2:59	0	4.3	343
120	68	4/18/2018	2:58	0	4.1	320
125	69	5/30/2018	1:00	0	4.2	228
90	82	6/5/2018	2:58	0	5.1	414
99	91	5/8/2018	2:59	0	4.6	277
89	90	5/30/2018	2:45	0	5.5	442
91	91	5/15/2018	2:58	0	5.9	391

122	63	5/29/2018	2:58	0	5.1	370
108	77	5/16/2018	3:00	0	3.4	294
99	75	6/19/2018	2:58	0	4	301
91	86	6/19/2018	2:57	0	2.8	272
91	96	7/20/2018	3:00	0	3.4	284
106	86	9/4/2018	2:59	0	3.3	254
102	75	9/5/2018	3:00	0	5.1	354
110	83	9/21/2018	2:58	0	3.6	305
103	77	11/2/2018	2:59	0	6.8	531
115	60	11/21/2018	2:58	0	5.4	391
123	73	10/16/2019	3:00	0	4.9	360
139	66					
78	77	4/8/2020	2:45	0	5.5	304
97	92	4/24/2020	3:00	0	4.7	284
96	95	5/6/2020	2:15	0	5.2	419
138	64	5/15/2020	2:45	0	5.3	405
81	92	5/29/2020	2:57	0	3.8	273
97	74	6/17/2020	3:00	0	3.5	240
101	69	7/3/2020	2:30	0	4.8	427
103	64	7/8/2020	3:00	0	4.4	305
91	70	2020/02/13	2:56	0	4.9	361
88	64	2020/05/22	2:45	0	4.6	304
127	59	2020/06/03	2:58	0	6	380
55	61	10/14/2020	2:59	0	5.5	287
50	50	2020/06/12	2:45	0	5	307
62	51	2020/08/07	2:57	0	5.8	286
77	45	7/30/2020	03:00	0	4.6	297
90	63	9/30/2020	2:59	0	5.9	347
77	69	11/20/2020	2:59	0	6.3	364
47	53	3/27/2019	02:58	0	3.7	211
82	65	11/19/2020	3:00	0	5.5	265
-	-	3/22/2021	2:30	0	4.1	248
72	82	2021/01/08	3:00	0	3.8	256
33	70	2021/01/14	2:56	0	5.2	294
77	52	5/12/2021	2:57	0	5.8	353
82	61	8/27/2021	3:00	0	5.2	287
66	54	9/3/2021	2:59	0	5	312
119	61	9/10/2021	2:57	0	4.3	255
45	41	9/13/2021	2:59	0	5.5	422
65	59	9/16/2021	2:59	0	5	285
63	51	9/29/2021	2:58	0	4.7	279
101	66	11/3/2021	02:58	0	2.3	142
55	38	5/2/2018	02:57	0	4.5	254

VO2%p-rest	V'CO2-rest	HR-rest	O2/HR-rest	Psys-rest	Pdia-rest
14	253	93	4	111	75
10	222	77	2.3	137	74
10	235	109	2.4	116	81
11	193	84	2.8	131	77
16	218	77	3.4	133	81
19	333	98	3.5	119	84
16	223	73	3.8	141	96
17	303	80	4.2	141	84
18	242	75	3.7	164	95
20	238	78	4.1	147	108
21	292	72	4.3	181	97
10	200	105	1.7	139	81
18	377	81	4.5	187	106
12	232	85	2.9	135	88
18	323	86	3.7	183	104
18	162	77	3.5	103	45
21	312	69	5.9	121	76
23	281	87	4.5	129	73
8	85	63	2	175	94
18	220	74	4.3	163	95
9	161	75	2.8	145	97
15	191	62	3.7	129	74
4	64	69	1	137	73
14	195	83	3.5	126	62
17	296	73	4.8	128	100
12	174	54	4.5	130	87
19	322	84	4.5	153	97
21	285	83	4.5	140	85
15	214	60	4.6	134	103
9	155	83	2.3	144	88
15	264	68	4.7	128	76
15	221	56	5.3	125	88
19	599	86	5.6	151	96
16	301	59	5.8	168	98
16	273	65	4.9	138	85
17	171	77	3		
17	371	91	4.6	122	72
13	235	83	3.3	151	100
20	392	96	4.6	187	93
19	237	85	4.6	130	100

19	295	53	7	168	84
11	259	91	3.2	138	106
15	231	88	3.4	128	86
11	179	88	3.1	125	110
12	297	76	3.7	121	85
13	281	72	3.5	138	78
18	253	81	4.4	109	83
14	236	59	5.2	132	88
25	428	66	8	134	115
21	322	93	4.2	159	98
19	310	84	4.3	176	92
22	237	89	3.4	153	83
14	187	82	3.5	144	114
16	311	65	6.4	129	80
17	381	79	5.1	135	81
13	235	86	3.2	156	65
13	211	75	3.2	147	78
18	270	79	5.4	123	88
17	221	84	3.6	152	78
15	272	100	3.6	117	90
24	257	77	3.9	166	88
31	264	88	4.3	167	107
25	226	62	4.6	147	78
25	279	87	3.5	123	63
18	186	85	3.4	150	67
23	222	107	2.8	159	112
24	345	80	4.3	114	86
24	308	71	5.1	125	70
12	172	77	2.7	116	70
20	253	75	3.5	140	80
21	178	70	3.5	151	73
21	166	77	3.3	150	84
18	231	93	3.2	159	76
27	241	80	4.4	159	90
26	259	96	3	143	87
28	249	91	3.4	148	86
17	257	76	3.4	125	70
25	314	102	4.1	143	83
30	282	60	4.8	沒量到	沒量到
23	207	82	3.4	沒量到	沒量到
8	113	98	1.4	121	92
25	193	82	3.1	158	94

t-in-rest	t-ex-rest	t-tot-rest	borg-leg-rest	borg-SOB-rest	EqO2_rest
3.16	1.98	5.14			24.7
2.03	2.38	4.41			69.6
1.43	2.28	3.72			41
2.33	3.5	5.83			36.7
1.2	1.73	2.93			47.2
1.39	1.9	3.29			41.5
1.43	1.63	3.06			31.1
1.53	3.38	4.91			34.7
1.05	1.61	2.67			37.1
0.95	2.5	3.45			38.6
1.59	2.58	4.17			43.2
1.14	1.59	2.73			53.9
2.99	4.38	7.37			39.8
2.19	3.03	5.22			33.6
1.57	2.3	3.87			39.8
1.24	2.1	3.34			28.6
1.36	2.59	3.94			26.8
1.05	2.18	3.24			26.3
1.48	2.07	3.54			30.5
2.3	2.13	4.43	0	0	24
2.92	3.95	6.87			26.3
2.93	3.14	6.07	0	0	31.9
2.53	3.24	5.77	0	0	64.1
2.85	2.9	5.75			23.3
2.49	2.91	5.4			34.6
2.03	2.07	4.1	0	0	25.6
1.61	1.71	3.32	0.5	0	35.5
2.64	2.55	5.19	0	0	29.2
1.07	1.48	2.55	0	0	32.4
3.29	3.83	7.12	0	0.5	27.8
0.85	1.82	2.67	0	0	30
1.4	2.19	3.59	0	0	26.9
2.3	3.69	5.98	0	0	58.9
0.99	1.67	2.66			43.4
1.1	2.21	3.32			30
1.15	1.81	2.96			31.3
1.2	1.69	2.89			35.2
1.84	3.33	5.17	0	0	35
1.03	1.56	2.6			35.7
1.35	1.82	3.16			24

1.02	3.46	4.48			28.6
1.38	2.38	3.77	0	0.5	37.2
1.19	2.78	3.97			32.2
1.12	6.63	7.75			17.9
4.13	3.92	8.05	0	0.5	34.9
2.45	3.87	6.32	1	0	41.7
0.88	1.4	2.28	0	0	39.5
1	1.62	2.62	0	0	32.8
3.04	1.72	4.76			27
0.96	1.88	2.84			34.9
1.26	1.47	2.73	0	0	37.9
1.09	1.06	2.15	0	0	52.9
5.86	0.56	6.42	0	0	19.9
1.34	2.26	3.6	0	0	25.4
2.58	2.06	4.64	0	0	33.5
3.22	1.88	5.1	0	0	29.1
0.97	2.25	3.22	0	0	42.2
1.25	1.15	2.4	0	0	25.8
5.08	3.6	8.68	0	0	27.2
3.4	2.05	5.45	0.5	0.5	27.8
0.84	2.78	3.61	1	0	34
0.92	1.49	2.41	0	0	23.9
1.55	1.9	3.45	0.5	1	33.9
0.97	1.02	1.99	2	2	38.6
0.99	1.33	2.32	1	1	20.7
0.92	1.05	1.97	0	0	34.6
1.37	1.46	2.83	0	0	40.7
1.18	1.62	2.79	0	0	40.1
1.28	1.56	2.84	0	0.5	40
1.84	2.41	4.25	0.5	0.5	31.3
2.39	2.17	4.57	0	0	21.8
1.44	2.01	3.44	0.5	0.5	28.3
1.75	2.47	4.22	0.5	0	43.3
1.68	1.53	3.21	0	0	32.3
0.84	1.28	2.12	0.5	0	35.5
1.55	1.69	3.24	0	0	32.2
1.55	2.01	3.56	0	0	43.9
1.05	1.08	2.13	0	0	34
1.53	1.89	3.41	0	0	46.8
0.69	0.91	1.6	0	0	45.8
0.93	1.41	2.34	0	0	23.9
1.08	1.61	2.69	0	2	32.1

EqCO2_rest	PETO2_rest	PETCO2_rest	SpO2_rest	V'E_rest	VTex_rest
36.3	108.47	29.41	96	10	0.856
55.5	130.26	22.25	99	13	0.977
45.6	120.41	25.55	97	12	0.734
44.7	113.71	29.12	97	9	0.91
56.2	116.66	27.88	98	14	0.669
42.9	121.15	27.58	98	16	0.855
38.3	108.9	33.03	98	10	0.504
38.5	115.2	30.58	97	13	1.027
42.8	119.02	27.84	95	12	0.531
51.3	119.53	24.44	99	13	0.773
45.6	120.27	28.12	98	14	0.995
48	126.67	23.67	96	11	0.508
38.5	116.39	30.94	97	15	1.854
35.6	115.08	30.63	97	9	0.789
38.9	118.7	32.04	97	14	0.88
47.5	103.99	29.49	97	9	0.499
34.8	103.84	37.16	98	12	0.784
36.6	100.44	36.27	95	12	0.624
45.2	117.09	22.63	97	5	0.297
34.6	106.43	31.29	96	9	0.633
34.5	101.36	35.85	98	6	0.705
38.7	111.15	29.63	98	8	0.82
70	123.26	22.37	98	5	0.504
34.8	100.87	32.94	96	8	0.721
41.1	113.01	32.44	97	13	1.166
35.5	101.95	35.27	98	7	0.493
41.7	110.99	32.1	98	15	0.813
37.9	109.78	29.99	99	12	1.004
41.5	110.35	29.87	96	11	0.447
34.2	105.94	35.15	99	6	0.7
36.6	115.45	27.66	94	11	0.5
35.8	100.04	35.44	97	9	0.544
47.4	126.86	22.25	98	29	2.901
49.5	116.24	28.83	98	16	0.729
35.2	109.55	34.17	97	11	0.601
36.3	123.57	21.17	94	11	0.793
39.2	112.83	32.39	96	16	0.771
41.1	114.41	30.16	98	10	0.903
40.3	122.79	23.71	97	17	0.753
39.5	101.12	35.01	96	11	0.563

35.9	109.48	34.69	98	12	0.861
42.2	119.43	25.79	99	12	0.756
42	109.24	30.56	98	11	0.712
27.1	94.5	37.94	98	5	0.698
33.3	115.17	31.51	98	10	1.396
37.6	117.22	26.22	97	11	1.185
55.3	120.72	19.42	97	16	0.602
42.4	112.7	27.62	97	12	0.508
33.6	111.61	31.81	97	15	1.208
42.3	112.34	32.1	98	15	0.714
44.1	112.22	33.63	96	15	0.691
67.8	122.79	20.59	98	18	0.645
30.2	94.27	37.47	99	6	0.676
34.2	103.01	33.91	98	12	0.707
35.7	117.07	27.99	96	15	1.122
33.7	116.57	26.61	98	9	0.744
48.1	117.27	27.03	98	11	0.613
40.9	113.16	24.1	98	13	0.512
37.6	106.08	31.28	98	9	1.272
36.9	111.09	29.5	紀錄失敗	11	0.98
40.2	114.57	29.75	紀錄失敗	11	0.692
34.5	104.36	32.04	95	11	0.436
43.1	109.47	32	紀錄失敗	11	0.63
42.4	117.31	28.47	91	14	0.462
31.7	101.22	33.09	91	8	0.299
46.2	116.01	23.56	91	12	0.407
41	118.53	29.06	98	16	0.737
47.3	111.07	31.17	98	16	0.748
49	118.86	24.78		10	0.469
32.8	113.28	33.49	98	9	0.658
30.5	108.13	31.31	98	6	0.482
43.8	110.87	26.32	97	8	0.486
55.3	118.53	23.44	97	14	0.966
47.3	108.94	28.18	96	13	0.68
39.4	114.87	30.1	95	12	0.431
40.4	112.15	28.8	94	11	0.612
43.6	117.26	29.28	94	12	0.736
45.7	118.75	29.66	92	16	0.58
47.4	121.7	24.95	97	15	0.83
61.6	123.67	22.74	90	15	0.411
29.9	112.84	30.61		5	0.202
42.3	114.11	27.35	98	10	0.435

BF_rest	BR_rest	Time-un	Load-un	VO2/kg-un	V'O2-un	VO2%p-un
12	92	6:00	0	6.2	446	17
14	84	5:59	0	5.3	402	23
16	92	5:58	0	5.7	428	16
10	93	6:00	0	5.8	489	22
20	88	5:58	0	7	393	23
18	86	5:57	0	5.3	373	21
20	91	5:57	0	7.3	445	25
12	87	5:58	0	8.2	662	33
22	90	5:58	0	7.7	581	37
17	86	5:59	0	7.6	516	32
14	84	5:59	0	7.9	591	40
22	89	5:58	0	9.7	652	36
8	84	5:59	0	9.1	732	36
11	93	5:59	0	6.7	523	26
16	87	5:59	0	9.6	595	34
18	91	5:58	0	7.8	428	29
15	88	5:58	0	9.5	596	31
19	89	5:59	0	9.2	589	34
17	96	5:59	0	8.9	581	36
14	92	5:59	0	11.5	734	41
9	95	5:58	0	5.6	442	20
10	91	6:00	0	7.9	410	27
10	95	5:57	0	9.9	663	36
10	94	5:58	0	9	673	32
11	91	5:59	0	7.8	539	25
15	94	5:57	0	9.7	580	30
18	90	5:58	0	11.1	632	32
12	90	5:59	0	7.7	472	27
24	90	5:59	0	5.9	444	24
8	96	5:58	0	4.4	313	14
22	91	5:57	0	5.1	404	18
17	93	5:58	0	7	393	20
10	83	5:54	0	6.2	510	20
23	87	5:58	0	7.4	589	28
18	86	5:56	0	8.4	652	33
14	82	3:00	0	7	383	28
21	87	5:58	0	9.3	755	30
12	91	5:54	0	8.9	532	26
23	85	5:45	0	10.1	808	37
19	92	5:58	0	9	592	29

13	88	5:45	0	8.5	623	31
16	91	6:00	0	6.2	538	19
15	90	5:57	0	8.6	645	32
8	96	6:00	0	2.7	257	10
7	93	5:59	0	5.8	478	21
9	90	5:59	0	8.3	642	33
26	84	5:58	0	9.5	663	33
23	91	5:57	0	6.3	533	24
13	84	5:58	0	7.9	613	29
21	84	5:59	0	9.2	659	35
22	85	6:00	0	7.2	533	28
28	80	5:59	0	11	605	44
9	95	5:45	0	7.1	427	21
17	92	5:15	0	7.9	643	25
13	85	5:58	0	10.7	826	34
12	92	5:57	0	6.4	455	21
19	84	5:59	0	10.1	688	38
25	89	5:59	0	8.7	776	32
7	91	5:59	0	10.8	747	40
11	91	5:00	0	8.3	612	25
17	80	5:30	0	7.6	506	40
25	79	6:15	0	10.7	675	54
17	87	5:58	0	7.9	411	37
30	71	5:59	0	10.5	639	52
26	84	5:59	0	11.7	580	37
30	78	06:00	0	8.5	553	44
21	79	5:15	0	9.6	565	39
21	86	5:59	0	8.5	496	33
21	87	05:45	0	6.6	379	22
14	84	5:45	0	8.4	404	30
13	85	06:00	0	10	602	51
17	90	5:58	0	6.7	450	37
14	84	5:30	0	9.3	528	32
19	84	5:59	0	10.2	624	47
28	75	5:45	0	8.9	490	45
19	80	6:00	0	10	617	55
17	83	5:58	0	7.9	471	32
28	69	5:45	0	7.6	584	34
18	75	5:59	0	8.8	503	53
37	72	6:15	0	9.1	535	43
26	93	05:59	0	4.8	292	17
22	78	06:15	0	7.8	446	44

V'CO2-un	HR-un	O2/HR-un	Psys-un	Pdia-un	t-in-un	t-ex-un
350	89	5	111	75	2.82	1.61
308	81	5	171	89	1.34	2.51
403	103	4.2	116	86	1.03	1.5
392	94	5.2	131	77	1.64	3.07
361	77	5.1	133	81	1.19	1.48
405	104	3.6	119	84	1.41	1.39
355	77	5.8	151	93	1.36	1.55
591	89	7.4	171	77	1.24	1.84
513	87	6.7	164	95	1.01	1.43
395	82	6.3	154	123	1.43	2.34
473	128	4.6	193	101	1.26	1.58
588	110	5.9	139	81	0.94	1.21
558	92	8	187	106	1.09	1.47
452	88	5.9	135	88	1.19	1.93
455	97	6.1	183	104	1.51	3
320	75	5.7	120	57	1.2	1.91
485	72	8.3	151	85	1.79	1.93
528	94	6.3	129	73	1.11	1.5
379	73	8	181	93	1.04	1.72
527	83	8.8	163	95	1.02	1.32
319	77	5.7	145	97	1.41	2.18
281	72	5.7	129	74	1.8	1.92
529	84	7.9	137	73	1.52	2.37
586	94	7.2	139	71	0.89	1.64
423	73	7.4	143	94	1.46	1.9
467	59	9.8	130	87	1.06	1.53
594	96	6.6	153	97	2.02	2.4
390	92	5.1	140	85	1.34	1.92
379	73	6.1	134	103	1.16	1.63
240	89	3.5	152	85	1.84	1.62
281	78	5.2	128	76	1.06	1.36
325	70	5.6	125	88	2.82	1.72
435	85	6	151	96	3.47	4.6
533	69	8.5	168	98	1.17	1.51
553	75	8.7	138	85	1.11	1.7
348	80	4.8	157	137	0.98	3.89
701	99	7.6	122	72	1.03	1.31
544	88	6.1	140	94	1.81	3.12
841	109	7.4	204	155	2.41	2.88
407	83	7.1	135	107	1.15	1.64

436	66	9.4	168	84	0.89	1.57
408	96	5.6	136	123	2.01	3.1
504	89	7.2	128	86	1.12	1.67
159	92	2.8	125	110	1.24	1.52
368	80	6	121	85	2.16	2.81
571	81	7.9	134	75	1.45	2.39
538	89	7.4	139	89	0.84	1.2
427	75	7.1	139	93	1.07	1.64
477	67	9.2	152	94	1.15	1.82
561	104	6.3	168	91	1.09	1.62
465	88	6.1	176	92	1.03	1.26
461	100	6.1	153	83	1.53	1.16
341	91	4.7	144	114	1.97	2.45
514	70	9.2	106	72	5.56	5.01
730	89	9.3	154	87	0.93	1.81
403	92	4.9	156	65	1.84	2.42
592	92	7.5	147	78	0.92	1.38
505	87	8.9	148	90	1.03	1.29
564	89	8.4	152	78	1.35	2.01
515	105	5.8	130	85	1.34	2.04
432	87	5.8	166	88	1.08	1.78
506	96	7	167	107	0.91	1.25
323	79	5.2	147	78	1.65	1.75
558	114	5.6	123	63	0.62	0.78
436	99	5.9	150	67	0.74	0.77
439	118	4.7	146	87	0.68	0.7
519	85	6.6	114	86	1.69	1.51
480	83	6	125	70	1.11	1.4
302	85	4.5	116	70	1.31	1.58
321	75	5.4	129	83	1.6	2.84
416	91	6.6	151	73	0.98	1.17
381	91	4.9	150	84	1.54	1.95
403	102	5.2	159	76	1.31	2.69
497	92	6.8	159	90	1.08	0.9
475	102	4.8	143	87	0.58	0.9
528	106	5.8	158	100	1.3	1.38
372	78	6	125	70	0.94	1.06
537	145	4	143	83	1.11	1
553	71	7.1	209	116	1.03	1.42
473	94	5.7	217	99	0.77	0.91
263	106	2.8	121	92	0.84	1.17
383	97	4.6	156	83	0.93	1.27

t-tot-un	borg-leg-un	borg-SOB-un	EqO2-un	EqCO2-un	PETO2-un
4.42	0	0	24.5	31.2	118.29
3.85	0	0	30.3	39.6	108.91
2.52	0	0	40.8	43.3	117.48
4.72	0	0	29.6	36.8	108.63
2.68	0.5	1	43.2	47.1	115.91
2.81	0	0	44.1	40.6	124.79
2.91	0	0	28.8	36.2	106.15
3.08	a	a	32	35.8	113.76
2.44	0	0	32.5	36.9	115.8
3.77	0	0	31.5	41.1	114.2
2.84	0	0	34.9	43.6	114.47
2.16	0	0	31.9	35.4	110.82
2.56	1	0	25.9	34	104.49
3.12	0	0	28.9	33.5	108.68
4.51	0	0	23.5	30.7	103.81
3.11	0	0	30.5	40.8	108.89
3.72	0	0	25.6	31.5	114.03
2.62	0.5	0	28.7	32	107.29
2.76	0	0	25.1	38.5	105.65
2.34	0	0	25.7	35.7	104.92
3.59	0	0	23.9	33.1	100.94
3.72	0	0	28.7	41.9	105.9
3.89	0	0	27.7	34.7	104.78
2.53	0	0	29.9	34.3	108.44
3.35	0	0	32.3	41.2	109.12
2.59	0	0	27	33.5	104.52
4.42	1	0	33.1	35.1	112.33
3.25	0	0	29	35.1	107.4
2.79	0	0	31.4	36.8	109.23
3.45	0	0.5	28.2	36.8	112.81
2.42	0	0	23.3	33.6	104.68
4.54			25.2	30.5	100.78
8.07	0.5	0.5	25.7	30.1	104.08
2.67	0	0.5	38.5	42.5	114.61
2.81	0	0	27.4	32.2	108.06
4.87	0	0	34.2	37.7	121.39
2.34	0	0.5	32.7	35.2	112.4
4.93			42.7	41.8	121.48
5.29	0	0	31.4	30.2	113.74
2.79	0	0	24.8	36.1	104.75

2.46	0	0	26.7	38.2	106.84
5.11			26.8	35.3	108.71
2.79	0.5	0	30.3	38.9	110.02
2.76	0	0	20	32.3	95.14
4.97			22.2	28.8	103.15
3.84			30.9	34.8	106.81
2.04	0	0	37	45.5	118.79
2.71	0	0	30.8	38.5	107.99
2.97	0	0	26.4	33.9	108.73
2.71	0	0	30.8	36.2	109.81
2.28	1	1	36.2	41.5	117.24
2.69	0.5	0	37.3	48.9	126.93
4.42	0	0	22.9	28.6	104.11
10.57	0	0	25	31.2	108.79
2.74	0	0	32.2	36.4	109.57
4.26	0	0	30.7	34.7	109.12
2.3	0	0	39.5	45.9	116.36
2.32	0	0	23.9	36.8	103.5
3.37	0	0	26	34.4	104.45
3.38	0.5	0.5	32.2	38.2	108.91
2.86	1	0	30.2	35.3	109.83
2.16	0	0	23.3	31	102.97
3.39	1	2	30	38.2	105.05
1.4	2	2	34.6	39.6	113.52
1.51	2	1	23.9	31.9	110.48
1.38	0	0	36.5	46	117.31
3.2	0	0	31	33.7	113.47
2.51	0.5	0.5	40.1	41.4	111.93
2.89	0	0.5	37.8	47.4	115.11
4.44	0.5	0.5	23.4	29.4	103.23
2.15	0	0	20	29	98.56
3.48	0.5	0.5	32.7	38.6	114.73
4	0.5	0	36.7	48.1	117.06
1.98	0	0	39.5	49.7	116.78
1.49	0.5	0	38.7	39.9	116.77
2.68	0	0	30.6	35.8	108.28
2.01	0	0	33.8	42.8	110.28
2.11	0	0	40.3	43.9	115.25
2.45	0	0	49.8	45.3	121.26
1.68	0	0	48.5	54.9	123.71
2.01	0	0	31.4	34.8	115.84
2.2	0	2	34.2	39.7	114.78

PETCO2-un	SpO2-un	V'E-un	VTex-un	BF-un	BR-un	Time_AT
24.16	97	12	0.876	14	91	15:30
32.12	96	13	0.853	16	84	10:15
29.26	98	19	0.805	24	87	12:00
32.05	98	15	1.206	13	89	12:30
30.67	98	19	0.829	22	84	12:15
26.16	98	18	0.84	21	84	10:00
34.64	99	14	0.693	21	88	11:45
32.1	97	23	1.157	19	76	11:15
30.69	96	21	0.839	25	82	13:45
28.69	98	17	1.091	16	82	10:30
29.32	98	22	1.044	21	75	11:45
35.36	96	23	0.817	28	77	11:00
35.06	97	21	0.878	23	78	10:15
34.49	96	16	0.857	19	87	10:30
37.78	96	15	1.12	13	86	11:45
30.93	98	14	0.747	19	86	12:00
30.36	98	16	1.016	16	84	10:45
37.39	96	18	0.806	23	82	9:00
29.45	96	16	0.742	22	86	11:00
33.79	98	21	0.805	26	82	11:15
35.01	96	12	0.701	17	91	12:15
30.51	98	13	0.8	16	85	12:30
36.22	98	19	1.261	15	83	9:45
33.88	97	22	0.919	24	82	12:00
33.21	98	19	1.043	18	87	12:15
36.18	98	17	0.746	23	86	11:00
32.87	99	22	1.609	14	85	14:00
34.33	98	15	0.811	18	87	10:15
31.79	97	15	0.719	21	86	10:00
28.68	98	10	0.578	17	94	12:30
32.33	97	11	0.45	25	91	11:00
37.53	98	11	0.82	13	92	11:15
36.06	99	14	1.833	7	92	11:45
30.48	98	24	1.08	22	80	11:00
35.07	96	19	0.906	21	75	11:00
23.95	94	14	1.134	12	77	8:30
33.88	97	26	1.033	26	79	11:45
27.57	98	24	1.937	12	81	12:30
32.88	96	26	2.311	11	77	9:45
36.26	97	16	0.752	22	88	13:15

33.57	95	18	0.753	24	80	10:30
30.4	98	15	1.297	12	88	8:45
30.81	97	21	0.981	21	81	10:45
36.26	98	7	0.307	22	95	10:30
35.08	97	11	0.948	12	93	10:30
34.86	97	21	1.341	16	82	9:30
22.21	97	27	0.902	29	74	10:30
31.64	97	18	0.812	22	86	10:15
34.81	98	18	0.87	20	82	10:30
33.85	99	22	0.987	22	76	10:15
28.95	96	21	0.805	26	78	10:15
17.27	82	24	1.082	22	74	10:30
36.31	98	11	0.789	14	92	13:00
30.88	98	16	2.898	6	89	11:30
32.93	96	28	1.283	22	70	12:45
33.43	97	15	1.061	14	87	10:15
26.84	99	29	1.112	26	58	7:30
30.49	98	20	0.786	26	82	8:45
33.08	96	21	1.159	18	80	11:45
34.15	紀錄失敗	21	1.178	18	82	12:45
32.71	紀錄失敗	17	0.798	21	71	8:30
36.11	94	18	0.636	28	66	14:45
34.93	紀錄失敗	14	0.769	18	84	12:45
31.81	80	25	0.587	43	47	9:30
30.25	89	17	0.419	40	66	12:30
23.32	88	23	0.535	43	59	09:30
32.17	98	19	1.003	19	74	8:45
33.34	99	22	0.901	24	81	11:30
27.21		16	0.76	21	80	10:45
37.24	99	10	0.77	14	82	12:45
38.15	98	14	0.502	28	66	10:00
29.14	97	16	0.926	17	80	9:45
23.53	86	20	1.361	15	76	9:00
25.63	95	27	0.884	30	66	11:15
31.56	95	22	0.54	40	56	8:30
33.94	92	20	0.914	22	64	11:45
30.37	90	18	0.603	30	75	9:45
29.61	89	26	0.898	28	51	10:15
27.12	95	27	1.094	24	53	13:15
25.21	88	28	0.797	36	49	11:00
30.59		11	0.377	30	84	11:30
29.36	93	17	0.629	27	61	09:15

Load_AT	VO2/kg_AT	V'O2_AT	VO2%p_AT	V'CO2_AT	HR_AT
179	30.2	2173	83	2006	139
59	8.5	650	37	580	94
113	13.4	1002	37	1018	148
91	12.5	1050	47	992	107
61	12.5	698	42	715	105
55	11	780	44	672	110
57	13.5	825	47	737	94
99	13.7	1107	55	1089	109
76	11.2	841	54	814	99
63	12.9	877	55	704	105
80	14.2	1066	72	859	125
70	16.6	1114	61	1024	126
59	13.6	1088	54	856	107
63	12.2	952	47	822	106
56	12.7	785	45	712	117
59	15.7	865	58	713	105
66	15.2	960	50	853	99
41	12.3	785	46	704	95
70	13.6	881	54	605	105
74	18.6	1188	66	891	105
88	13.7	1086	48	934	108
64	19.6	1018	67	826	105
52	11.6	778	42	713	94
112	17.9	1345	64	1343	123
88	18.2	1259	59	964	112
70	16.5	992	51	849	79
79	18.5	1053	53	1059	124
60	13	796	46	749	113
56	10	702	38	681	89
152	26	1843	82	1688	125
93	11.9	942	43	788	103
73	16.4	917	47	853	110
81	13	1064	41	976	105
70	13.6	1089	52	919	90
70	13.2	1030	53	989	96
50	13.5	740	54	615	93
80	16.1	1305	52	1199	120
63	16.2	971	47	821	107
70	12.6	1010	46	828	121
71	20.2	1330	65	1274	105

63	14	1023	51	820	82
51	8.9	776	28	410	105
66	12.8	957	47	775	98
84	12.9	1248	48	974	114
63	10.3	851	37	764	96
48	10.1	774	40	704	92
44	10.8	754	37	663	91
80	13.4	1143	51	956	100
63	10.8	841	40	695	71
60	15.2	1094	58	830	112
44	13.2	977	51	777	104
44	11.4	627	46	560	111
69	15	899	45	807	133
103	19.4	1573	61	1233	111
95	18.5	1422	59	1174	108
60	8.4	598	28	512	101
14	9.6	655	36	545	95
38	10.2	912	37	705	93
56	12	829	45	676	102
66	14.2	1053	43	864	123
24	8.4	557	44	465	94
41	14.3	900	72	731	122
31	11.6	601	53	541	95
34	10.7	652	53	597	122
30	12.2	606	38	482	104
34	9.9	647	51	524	124
26	14.1	830	58	601	92
54	13.5	784	52	838	98
46	10.2	583	34	513	96
31	12	575	43	536	93
39	13.2	791	66	604	96
36	7.8	524	44	475	101
29	11.5	655	40	543	105
51	14.2	868	66	750	113
24	11.5	630	58	532	106
56	13.3	825	74	762	130
53	18.5	1110	76	806	86
42	11.5	882	52	760	131
34	13	740	78	685	93
23	10.5	617	50	554	103
54	11	669	40	609	129
15	8.5	486	48	411	100

O2/HR_AT	Psys_AT	Pdia_AT	t-in_AT	t-ex_AT	t-tot_AT
15.6	173	132	0.76	1.88	2.65
6.9	158	82	1.48	1.93	3.42
6.8	149	80	1.12	1.39	2.51
9.8	165	80	1.58	2.38	3.96
6.6	144	71	1.27	1.41	2.68
7.1	119	84	1.01	1.31	2.33
8.8	144	85	1.27	1.33	2.6
10.2	212	113	1.2	1.73	2.94
8.5	209	84	0.9	1.14	2.04
8.4	175	85	1.17	1.88	3.05
8.5	212	167	1.12	1.27	2.39
8.8	181	92	0.87	1.41	2.28
10.2	197	111	1.1	1.51	2.61
9	147	81	1.48	1.93	3.41
6.7	213	86	1.35	1.66	3.01
8.2	137	62	1.26	1.42	2.68
9.7	134	81	0.84	1.33	2.17
8.3	144	72	1.07	1.44	2.51
8.4	174	97	0.81	1.79	2.59
11.3	169	64	1.1	1.25	2.35
10.1	140	99	1.49	1.52	3.02
9.7	183	77	1.3	2.1	3.41
8.3	151	72	1.28	2.07	3.34
10.9	174	89	1.41	2.66	4.07
11.2	151	92	1.63	3.45	5.08
12.6	156	78	0.96	1.45	2.41
8.5	187	99	1.08	1.16	2.24
7	175	87	1.2	1.54	2.74
8.4	159	89	0.91	1.2	2.11
14.7	170	77	0.99	1.32	2.31
9.1	139	76	1.05	1.21	2.26
8.3	151	86	1.31	1.53	2.84
10.1	168	94	2.43	3.65	6.07
12.1	180	102	1.02	1.29	2.31
10.7	150	82	1.02	1.44	2.46
8	154	126	1.22	1.32	2.54
10.9	158	80	1.03	1.25	2.28
9.1	144	94	2.71	5.99	8.7
8.3	193	123	1.13	1.13	2.27
12.7	150	130	1.79	1.64	3.44

12.5	190	86	0.91	1.45	2.36
7.4	136	123	1.33	2.1	3.42
9.8	123	88	1.07	1.31	2.38
10.9	125	89	1.29	1.82	3.1
8.9	125	86	1.83	2.09	3.92
8.4	154	95	1.33	1.83	3.16
8.3	139	84	1.47	1.89	3.36
11.4	144	125	0.9	1.17	2.07
11.8	148	86	1.29	1.86	3.15
9.8	204	125	1.14	1.52	2.66
9.4	213	87	0.89	1.6	2.49
5.6	182	82	0.86	1.07	1.92
6.8	191	96	1.66	1.4	3.05
14.2	113	76	3.35	2.44	5.79
13.2	165	98	0.71	1.5	2.21
5.9	153	72	1.42	1.66	3.07
6.9	169	83	1.13	1.57	2.7
9.8	148	90	0.99	1.14	2.13
8.1	155	53	0.95	2.79	3.74
8.6	111	97	1.28	1.74	3.02
5.9	197	87	0.85	1.72	2.57
7.4	186	88	0.8	1.13	1.93
6.3	148	82	1.33	1.65	2.99
5.3	172	96	0.59	0.71	1.3
5.8	133	78	0.73	0.85	1.59
5.2	158	84	0.74	0.67	1.41
9	115	84	0.89	1.14	2.03
8	162	77	1.01	0.99	1.99
6.1	128	76	1.14	1.23	2.37
6.2	155	91	1.54	1.69	3.23
8.2	152	115	1.32	1.39	2.71
5.2	178	111	1.27	1.68	2.95
6.2	153	74	1.22	1.55	2.77
7.7	165	84	0.99	1.11	2.1
5.9	171	101	0.52	1.06	1.57
6.3	182	88	0.99	1.02	2
12.9	141	66	0.7	0.96	1.66
6.7	205	77	0.74	0.8	1.53
8	202	103	0.67	0.91	1.58
6	209	94	0.69	0.83	1.52
5.2	152	86	0.84	1.41	2.25
4.9	164	87	0.83	1.12	1.95

borg-leg_AT	borg-SOB_AT	EqO2_AT	EqCO2_AT	PETO2_AT	PETCO2_AT
2	2	22.5	24.4	100.28	42.62
0	0.5	30.7	34.4	110.75	33.98
5	4	32.7	32.2	113.92	34.81
2	0.5	32.7	34.6	112.64	31.85
3	2	36.3	35.5	113.39	35.89
0	0	33.2	38.6	117.85	28.17
3	0.5	27.7	31	105.11	38.15
4	4	32.3	32.8	113.75	34.55
2	0	35.1	36.3	119.63	29.58
0	0	31.6	39.4	114.77	29.48
7	7	30.6	37.9	111.13	32.12
2	0	26.8	29.1	107.46	38.64
1	1	23.5	29.9	99.73	39.22
1	1	25.7	29.7	105.83	36.92
2	1	27.5	30.3	110.3	36.64
1	1	27.2	33	109.17	33.01
2	0.5	26.1	29.4	106.01	40.64
2	0.5	27.1	30.2	105.47	38.44
0	0	22.7	33	97.82	35.51
0.5	0	22.6	30.2	101.58	38.44
0	0	25.1	29.2	102.83	38.48
1	0.5	27.3	33.6	108.64	32.51
4	3	30.8	33.6	108.52	36.63
5	5	26.3	26.3	100.44	42.86
3	2	23	30.1	93.57	41.6
0.5	0.5	24.8	29	102.57	38.91
4	3	34	33.8	111.56	35.1
1	1	30	31.8	110.08	35.51
3	3	30	34.8	109.36	33.1
3	2	24.9	27.2	105	38.55
1	0.5	25.5	30.5	105.77	34.98
2	0	24.9	26.8	101	41.07
2	2	24.4	26.6	104.67	38.24
0.5	0.5	31.7	37.6	109.41	32.47
0	0	26.3	27.4	106.41	40.06
0	0	27.6	33.2	115.09	28.55
5	4	29.2	31.8	110.84	35.52
3	0	26.9	31.9	101.66	37.51
0.5	0.5	24.4	29.8	108.34	35
1	0.5	29.1	30.4	114.7	36.99

1	0.5	25.5	31.8	106.73	36.47
0	0	16.7	31.6	88.21	34.45
2	2	26.9	33.3	105.41	35.33
1	0	20.9	26.9	98.21	39.97
1	4	27	30.1	109.03	34.12
2	0.5	28.7	31.6	104.9	36.68
0	0	37.6	42.8	114.08	28.28
2	1	28	33.5	105.56	33.56
0.5	0.5	25.8	31.2	108.4	36.36
0	2	25	33	104.12	35.57
2	1	26.9	33.8	103.15	37.85
3	1	47.7	53.4	119.4	25.43
1	0.5	23.4	26	105.94	38.35
0.5	0.5	20.6	26.2	98.27	37.55
0	0	26.7	32.4	103.27	37.11
1	0.5	27.2	31.8	106.07	35.31
0	0	33.2	39.9	110.13	30.89
0	0	28.7	37.1	109.02	30.62
2	2	25.9	31.8	103.28	35.57
6	4	26.5	32.3	104.25	36.52
2	0	29.3	35.1	108.37	33.83
3	4	23.9	29.4	104.86	39.66
5	5	32.6	36.2	108.49	35.69
3	3	34.8	38	112.72	33.47
4	3	24.6	31	105.48	35.4
3	4	34.9	43.1	115.28	25.49
1	0	22.1	30.5	104.09	34.26
4	4.5	38.5	36	113.21	34.86
-	-	36.6	41.6	115.71	29.53
2	2	29	31.1	110.44	34.91
0.5	1	21	27.5	99.31	40.25
2	0.5	39.8	43.9	119.91	25.87
2	1	43.6	52.6	118.46	24.14
3	2	34.7	40.1	112.52	31.02
4	4	32.1	38	110.07	35.17
6.56	6.56	31.5	34.1	109.67	34.88
0	3	23.5	32.3	101.56	35.64
4	5.93	39.4	45.8	114.71	29.44
7	8	39.7	42.9	116.69	29.49
2	2	50.7	56.5	124.42	24.77
2	2	25.9	28.5	111.05	34.1
3	4	35.7	42.3	115.63	28.33

SpO2_AT	V'E_AT	VTex_AT	BF_AT	BR_AT	Time-pk	Load-pk
95	51	2.232	23	62	19:00	246
97	21	1.207	18	75	14:47	124
98	34	1.442	24	77	16:00	189
98	35	2.337	15	74	16:10	144
98	27	1.202	22	77	18:15	121
98	28	1.075	26	75	17:15	159
98	24	1.06	23	79	18:00	119
97	37	1.819	20	60	14:34	162
96	32	1.074	29	73	20:21	143
98	29	1.48	20	70	15:45	138
98	34	1.368	25	61	17:15	159
96	32	1.204	26	69	15:35	136
97	27	1.183	23	71	14:59	127
96	26	1.46	18	80	16:30	149
96	23	1.152	20	78	15:54	98
98	25	1.122	22	75	16:50	107
97	27	0.976	28	73	15:48	139
95	23	0.958	24	78	13:00	98
96	22	0.935	23	82	14:55	126
96	29	1.122	26	75	17:45	141
96	29	1.439	20	78	17:15	155
97	29	1.648	18	67	18:30	124
98	25	1.406	18	78	14:13	116
97	36	2.47	15	73	15:00	170
97	30	2.525	12	80	18:00	170
98	26	1.058	25	78	19:00	184
99	38	1.405	27	74	19:27	134
98	25	1.159	22	78	15:54	141
97	26	0.905	28	76	15:00	127
97	48	1.843	26	70	17:00	258
95	26	0.975	27	79	17:00	205
98	24	1.15	21	82	16:15	145
99	27	2.696	10	85	18:30	177
98	36	1.399	26	70	13:26	105
96	29	1.182	24	63	14:45	123
94	22	0.936	24	64	9:27	60
97	40	1.517	26	68	16:15	145
99	27	3.865	7	78	15:43	96
95	27	1.003	26	77	13:49	148
97	40	2.29	17	69	19:15	131

95	28	1.098	25	70	13:45	110
97	14	0.808	18	89	13:18	138
97	28	1.094	25	75	14:45	124
96	27	1.423	19	80	14:06	153
96	24	1.573	15	84	14:15	116
96	24	1.243	19	79	14:48	124
96	30	1.66	18	70	16:44	106
97	34	1.175	29	73	15:08	173
98	23	1.209	19	76	14:15	117
98	29	1.287	23	68	17:09	158
95	28	1.159	24	72	13:30	106
98	32	1.028	31	65	13:45	76
98	22	1.139	20	82	17:06	110
96	33	3.191	10	79	16:30	199
96	40	1.47	27	58	17:02	156
97	18	0.905	20	84	15:09	130
99	23	1.05	22	67	11:45	57
98	28	0.999	28	75	15:15	131
97	23	1.411	16	78	13:43	76
紀錄失敗	29	1.475	20	75	17:52	118
紀錄失敗	18	0.769	23	68	12:34	65
91	24	0.763	31	55	21:00	70
紀錄失敗	21	1.045	20	76	15:29	45
78	26	0.562	46	45	12:30	64
89	18	0.465	38	65	16:15	48
86	26	0.6	43	55	12:30	64
98	20	0.691	30	72	15:20	92
98	32	1.072	30	71	14:00	78
	23	0.913	25	70	11:40	56
99	18	0.968	19	69	19:58	66
98	18	0.819	22	56	14:00	79
97	22	1.097	20	72	11:30	54
70	30	1.391	22	65	13:15	71
93	32	1.124	29	60	14:15	82
94	23	0.601	38	53	11:50	57
90	28	0.938	30	50	12:15	62
89	29	0.789	36	61	13:15	102
85	38	0.96	39	28	12:35	64
92	32	0.844	38	44	15:15	43
87	34	0.863	39	39	15:54	46
	19	0.72	27	72	16:45	106
93	20	0.635	31	56	14:45	41

VO2/kg-pk	V'O2-pk	VO2%p-pk	V'CO2-pk	HR-pk	O2/HR-pk
39.8	2868	110	3190	167	17.2
16.1	1220	70	1623	138	8.8
24.7	1855	69	2409	191	9.7
18	1511	68	1910	132	11.4
21.6	1210	72	1646	167	7.2
22.3	1582	89	1912	151	10.5
23.4	1429	82	1806	125	11.4
19.4	1573	78	1883	145	10.8
20.3	1523	97	1800	138	11
22.7	1545	97	1738	175	8.8
20.8	1564	106	1788	142	11
26.7	1789	98	1944	167	10.7
17.9	1433	71	1416	127	11.3
20.4	1592	79	1883	160	9.9
22.2	1374	80	1443	144	9.5
26.7	1470	99	1377	129	11.4
27.6	1741	90	2083	158	11
18.8	1205	70	1398	133	9.1
21.5	1396	86	1542	134	10.4
33.1	2120	118	2404	135	15.7
27.4	2162	96	2045	139	15.6
29.5	1536	101	1639	165	9.3
22.8	1530	83	1653	141	10.9
43	2050	154	3572	148	13.8
32.7	2260	106	2521	167	13.5
38.5	2307	118	2847	145	15.9
31.2	1777	89	1971	156	11.4
24.1	1472	84	1825	175	8.4
18.1	1356	73	1566	128	10.6
39.6	2810	125	3345	169	16.6
30.2	2389	108	2500	150	15.9
26.3	1474	75	1645	163	9
26.1	2140	82	2342	167	12.8
16.6	1331	64	1370	124	10.7
21.4	1669	86	1763	127	13.1
16.3	897	66	800	98	9.2
23	1860	74	1973	146	12.7
17.9	1071	51	1124	116	9.2
21.2	1692	77	2167	175	9.7
28.8	1899	93	1877	156	12.2

21.7	1584	80	1608	105	15.1
15.4	1343	48	1351	134	10
20.7	1552	77	1693	114	13.6
21.5	2081	80	1927	148	14.1
13.6	1130	49	1305	117	9.7
19.4	1496	77	1607	125	12
19.5	1368	68	1344	131	10.4
21.8	1851	82	2201	150	12.3
16.1	1253	60	1339	111	11.3
31.4	2259	120	2319	161	14
17.9	1325	69	1345	145	9.1
18.1	996	73	1064	136	7.3
22.1	1329	66	1574	165	8.1
34.2	2768	108	2893	158	17.5
28.4	2186	91	2217	132	16.6
14.7	1044	48	1201	151	6.9
13.6	927	51	1013	121	7.7
20.9	1861	76	1976	145	12.8
16.7	1150	62	1246	115	10
22	1628	66	1768	141	11.5
14.2	946	75	1016	138	6.9
20.6	1300	105	1250	165	7.9
14.9	773	69	847	104	7.4
13.3	811	66	976	152	5.5
16.3	810	51	756	121	6.7
14	908	72	920	153	5.9
24.4	1439	100	1772	153	9.4
21.1	1223	81	1407	122	10
12.3	699	41	646	102	6.9
20.6	988	74	1098	142	7
23.3	1398	118	1325	138	10.1
12.8	855	71	931	114	7.5
17.4	993	61	1096	134	7.4
19.2	1169	88	1248	134	8.7
15.3	841	77	866	123	6.8
14.8	915	82	826	134	6.8
20.3	1216	83	1448	106	11.5
13.4	1032	61	1078	134	7.7
13.6	773	81	757	99	7.8
14.1	831	67	781	131	6.3
17.2	1049	63	1240	175	6
12.1	692	68	640	124	5.6

Psys-pk	Pdia-pk	t-in-pk	t-ex-pk	t-tot-pk	borg-leg-pk
185	151	0.7	0.8	1.5	5
189	104	0.8	0.84	1.64	7
162	107	0.66	0.85	1.51	9
231	100	0.98	0.99	1.97	5
217	97	0.88	0.92	1.8	6
257	134	0.7	0.94	1.64	5
163	99	0.77	0.78	1.55	9
245	101	0.98	1.07	2.05	4
256	102	0.76	0.96	1.73	7
204	161	0.64	0.91	1.55	8
212	167	0.83	0.9	1.73	9
209	100	0.74	0.99	1.72	7
214	96	0.87	1.32	2.2	4
204	101	0.77	1.01	1.79	5
229	93	1.14	2.18	3.33	5
179	57	1	1.01	2.01	4
207	103	1.05	0.99	2.04	10
182	97	0.64	0.89	1.54	6
220	104	0.95	1.05	2	4
183	86	0.5	0.57	1.08	10
172	111	1.04	1.75	2.79	6
176	86	0.69	0.66	1.36	10
204	67	1.04	1.2	2.24	7
204	82	0.55	0.62	1.17	6
200	110	0.58	0.62	1.2	10
217	105	0.43	0.51	0.94	5
215	102	0.83	0.85	1.67	10
202	80	0.82	0.76	1.58	4
211	116	0.69	0.76	1.45	5
261	96	0.99	0.97	1.96	8
214	131	0.5	0.54	1.04	6
178	81	1.18	1.41	2.6	3
233	102	1.07	1.26	2.33	7
208	114	0.97	1.42	2.4	2
165	90	0.77	1.06	1.83	5
170	136	0.9	1.27	2.17	7
207	92	0.78	0.87	1.65	9
162	100	0.75	1.08	1.83	10
223	132	0.95	0.78	1.74	7
194	132	1.06	1.12	2.18	4

201	119	0.67	0.77	1.44	8
131	108	1	1.48	2.49	10
163	105	0.94	1.03	1.97	9
180	95	1.39	1.57	2.95	6
145	95	1.1	1.42	2.52	9
182	165	1.53	1.99	3.52	10
170	97	1.1	1.1	2.21	3
191	137	0.56	0.65	1.21	10
165	95	1.13	1.17	2.3	5
182	122	0.73	0.84	1.57	6
229	101	0.8	1.17	1.98	4
199	163	0.79	0.9	1.69	5
186	110	1.07	1.16	2.23	4
180	140	1.08	1.04	2.12	6
188	118	0.5	1.06	1.56	3
192	77	0.87	0.9	1.77	6
180	89	0.66	0.84	1.5	5
201	100	0.74	0.79	1.53	6
162	60	0.92	0.9	1.81	6
133	92	0.6	0.61	1.2	8
220	98	0.93	1.31	2.23	7
209	88	0.56	0.88	1.44	7
160	79	0.91	0.88	1.79	6
182	102	0.4	0.48	0.88	6
146	87	0.61	0.57	1.18	9
175	88	0.63	0.49	1.12	6
169	91	0.6	0.78	1.38	8
173	81	0.81	0.89	1.7	8
141	78	0.84	1	1.85	7
185	94	0.67	0.76	1.44	7
195	149	1.08	0.96	2.04	4
156	110	0.82	0.73	1.55	6
203	82	0.91	0.93	1.85	6
175	152	0.62	0.66	1.28	10
188	118	0.64	0.84	1.48	8
182	88	0.93	0.99	1.91	7
195	67	0.66	0.78	1.43	7
205	77	0.99	0.9	1.88	5
189	95	0.61	0.81	1.42	9
200	144	0.74	0.72	1.46	9
179	87	0.62	0.61	1.23	6
175	79	0.77	0.9	1.67	3

borg-SOB-pk	EqO2-pk	EqCO2-pk	PETO2-pk	PETCO2-pk	SpO2-pk
5	33.9	30.5	115.36	33.96	94
7	49	36.8	122.81	31.15	97
9	48	36.9	123.26	30.28	96
4	57.3	45.3	126.8	25.08	98
6	48.9	36	124.22	30.34	98
5	49.4	40.8	125.05	27.92	97
9	42.3	33.5	119.45	31.89	98
4	37.5	31.4	117.62	35.17	96
6	45.9	38.9	126.85	26.75	96
6	51	45.3	125.95	26.2	98
9	41.4	36.2	123.47	29.31	-
5	34	31.3	116.5	34.85	95
3	27.3	27.6	108.9	38.78	97
4	38.9	32.9	120.03	31.32	97
3	34.3	32.7	114.21	35	96
3	30.8	32.9	115.37	30.36	97
8	32.1	26.9	115.26	39.09	97
3	37.7	32.5	121.34	29.71	95
3	37.9	34.3	118.49	30.21	96
8	39.8	35.1	121.55	31.34	96
6	25.9	27.4	106.31	37.66	94
10	42	39.4	119.77	29.12	96
6	37	34.3	116.06	33.38	98
5	27	24.4	108	40.58	96
9	43.6	39	121.25	28.16	98
5	50.9	41.3	124.11	25.37	98
9	35.8	32.3	114.75	34.57	96
4	47.8	38.6	124.37	27.46	98
5	41.5	36	118.74	29.69	98
8	35.2	29.5	117	33.49	97
6	42.4	40.5	122.03	27.47	95
2	29.9	26.8	109.15	38.25	98
7	30.2	27.6	113.7	36.24	98
3	36.8	35.8	115.38	31.39	98
4	28.9	27.3	110.75	38.55	94
2	31.4	35.2	111.9	33.21	95
8	35	32.9	116.27	34.86	97
5	44.4	42.3	123.62	27.82	99
7	43.4	33.9	123.22	31.09	96
3	25.8	26.1	111.91	42.37	95

7	35.6	35.1	118.03	32.37	94
6	38.1	37.9	116.55	30.35	98
9	40.3	37	118.34	30.15	98
5	22	23.8	104.09	41.73	98
9	37.8	32.7	118	31.41	98
7	31.3	29.1	109.26	36.51	97
3	36.2	36.9	113.26	31.97	95
10	48.9	41.1	121.02	27.33	98
5	35.6	33.3	118.77	31.82	96
6	34	33.1	115.93	33.29	95
4	33.8	33.3	114.52	34.52	96
5	53.5	50.1	122.92	25.37	98
4	36.3	30.7	117.59	33.94	98
5	30.7	29.3	111.92	33.87	98
3	34.1	33.7	114.05	32.82	96
6	38.2	33.2	117.46	31.59	98
2	51.8	47.4	125.11	23.1	99
4	39.7	37.4	120.88	27.4	97
5	42.8	39.5	120.3	27.73	97
8	50.8	46.7	123.51	26.42	紀錄失敗
5	37.8	35.2	115.78	32.88	紀錄失敗
9	27	28.1	111.75	40.61	89
6	39.4	35.9	119.21	30.78	77
7	58.4	48.5	125.05	27.21	65
6	31.3	33.5	115.16	31.84	84
7	43.1	42.5	119.53	26.84	82
7	43.3	35.2	122.15	31.67	97
8.5	38.6	33.5	117.37	31.8	98
9	38.9	42.1	118.3	28.43	
9	37.2	33.5	118.03	32.63	83
3	24.8	26.1	105.69	40.78	98
3	56.8	52.1	128.81	20.76	98
6	62.6	56.7	127.3	20.67	82
10	51	47.7	124.67	24.22	91
9	37.1	36.1	115.87	34.84	91
7	30.1	33.3	107.62	36.32	89
9	40.5	34	112.41	38.8	92
9	52.8	46.4	121.18	29.71	81
9	44	44.9	118.81	28.79	90
7	48.9	52	121.44	28.08	78
5	35	29.6	116.53	35.65	
7	36.3	39.2	116.6	29.57	88

V'E-pk	VTex-pk	BF-pk	BR-pk	WRpk%	WRpk_pred	VO2MXPRD
100	2.503	40	24	124	198	2607.272727
62	1.705	37	27	111	112	1742.857143
92	2.317	40	39	93	203	2688.405797
89	2.91	30	36	94	153	2222.058824
62	1.843	33	47	98	116	1680.555556
81	2.205	37	27	134	119	1777.52809
63	1.632	39	46	98	121	1742.682927
61	2.084	29	35	118	136	2016.666667
72	2.083	35	38	148	95	1570.103093
81	2.105	39	25	136	101	1592.783505
67	1.932	35	24	183	87	1475.471698
63	1.816	35	37	110	124	1825.510204
41	1.5	27	56	91	136	2018.309859
64	1.915	34	50	106	137	2015.189873
48	2.686	18	54	83	118	1717.5
47	1.584	30	53	109	98	1484.848485
58	1.967	29	42	101	137	1934.444444
48	1.236	39	54	84	117	1721.428571
55	1.832	30	53	119	106	1623.255814
88	1.581	56	22	124	131	1796.610169
58	2.68	21	55	160	95	2252.083333
68	1.527	44	22	120	104	1520.792079
58	2.184	27	49	128	91	1843.373494
91	1.771	51	31	116	146	1331.168831
102	2.037	50	30	111	153	2132.075472
122	1.907	64	-2	125	142	1955.084746
66	1.843	36	55	91	147	1996.629213
73	1.923	38	37	117	120	1752.380952
59	1.432	41	45	104	123	1857.534247
101	3.292	31	37	158	163	2248
105	1.832	57	14	131	155	2212.037037
46	1.974	23	66	95	145	1965.333333
66	2.577	26	61	92	192	2609.756098
51	2.03	25	59	74	142	2079.6875
50	1.538	33	35	94	130	1940.697674
30	1.09	28	51	69	87	1359.090909
68	1.858	36	46	78	183	2513.513514
50	1.521	33	59	61	154	2100
76	2.195	35	34	95	153	2197.402597
51	1.851	28	61	89	146	2041.935484

59	1.423	42	36	80	137	1980
53	2.188	24	59	67	206	2797.916667
65	2.121	31	42	89	139	2015.584416
47	2.325	20	66	84	182	2601.25
44	1.863	24	71	67	164	2306.122449
48	2.812	17	58	95	131	1942.857143
51	1.893	27	49	71	141	2011.764706
94	1.89	50	24	111	156	2257.317073
46	1.78	26	52	81	145	2088.333333
79	2.086	38	13	124	127	1882.5
47	1.547	30	52	81	130	1920.289855
56	1.574	35	39	87	87	1364.383562
50	1.869	27	61	75	147	2013.636364
87	3.065	28	44	105	189	2562.962963
77	2.01	38	18	89	175	2402.197802
42	1.249	34	62	84	155	2175
51	1.272	40	27	46	125	1817.647059
77	1.961	39	33	76	172	2448.684211
51	1.556	33	50	60	126	1854.83871
86	1.73	50	27	65	183	2466.666667
38	1.402	27	34	92	71	1261.333333
38	0.912	42	27	100	70	1238.095238
33	0.98	33	62	69	65	1120.289855
52	0.761	69	-10	91	70	1228.787879
29	0.567	51	42	43	111	1588.235294
43	0.801	53	25	89	72	1261.111111
65	1.505	43	11	100	92	1439
50	1.408	35	56	80	98	1509.876543
29	0.906	33	62	46	123	1704.878049
40	0.949	42	32	74	89	1335.135135
37	1.248	29	11	118	67	1184.745763
51	1.323	39	37	84	64	1204.225352
64	1.983	33	24	64	111	1627.868852
63	1.342	47	21	104	79	1328.409091
34	0.84	41	31	95	60	1092.207792
30	0.948	31	47	105	59	1115.853659
52	1.247	42	28	110	93	1465.060241
64	1.245	51	-	60	107	1691.803279
37	0.873	42	36	96	45	954.3209877
43	1.058	41	22	64	72	1240.298507
40	0.824	49	42	93	114	1665.079365
28	0.769	36	38	91	45	1017.647059

HRPRDMX	HRpk_%	O2Pmx_pred	O2Pmx_prct	WRLT_MX	slope	slope_WK EFF_cut rest
179	93.3	14.56576943	1.180850767	0.904040404	10.465	10.369
152	90.8	11.46616541	0.76747541	0.526785714	6.3941	6.8512
178	107.3	15.10340335	0.642239353	0.556650246	6.9166	7.3544
159	83.0	13.97521273	0.81572998	0.594771242	7.6967	7.9705
154	108.4	10.91269841	0.659781818	0.525862069	7.6706	8.221
150	100.7	11.85018727	0.886061947	0.462184874	6.7567	7.0978
157	79.6	11.09989125	1.027037089	0.47107438	8.7598	8.7994
154	94.2	13.0952381	0.824727273	0.727941176	6.245	7.2143
144	95.8	10.9034937	1.008850952	0.8	7.6361	8.5158
145	120.7	10.98471383	0.801113269	0.623762376	8.6007	8.9128
142	100.0	10.39064576	1.058644501	0.91954023	7.3838	7.7673
156	107.1	11.70198849	0.914374511	0.564516129	8.9635	8.9635
157	80.9	12.85547681	0.879002791	0.433823529	8.3538	8.3538
157	101.9	12.83560429	0.771292085	0.459854015	7.2402	7.2402
155	92.9	11.08064516	0.857350801	0.474576271	7.6179	7.6179
150	86.0	9.898989899	1.151632653	0.602040816	9.9998	10.506
161	98.1	12.01518288	0.915508329	0.481751825	8.9571	9.4688
153	86.9	11.25116713	0.808804979	0.35042735	7.7803	8.5532
150	89.3	10.82170543	0.961031519	0.660377358	8.5142	8.986
157	86.0	11.44337688	1.371972642	0.564885496	9.401	9.7671
160	86.9	14.07552083	1.108307123	0.926315789	9.7207	9.8241
154	107.1	9.875273242	0.941746094	0.615384615	10.041	10.754
153	92.2	12.04819277	0.9047	0.571428571	8.4399	9.5003
164	90.2	8.116883117	1.70016	0.767123288	8.5989	7.7961
166	100.6	12.84382814	1.051088496	0.575163399	10.433	10.829
166	87.3	11.77761895	1.350018205	0.492957746	10.259	10.53
166	94.0	12.02788683	0.947797411	0.537414966	9.4079	10.656
155	112.9	11.30568356	0.74298913	0.5	7.716	8.3403
152	84.2	12.22062004	0.867386431	0.455284553	7.4802	8.0873
168	100.6	13.38095238	1.240569395	0.932515337	8.9831	9.321
166	90.4	13.32552432	1.193198828	0.6	8.4053	8.7947
169	96.4	11.62919132	0.773914518	0.503448276	7.7157	7.9664
173	96.5	15.08529536	0.848508411	0.421875	8.4871	8.9678
157	79.0	13.2464172	0.80776559	0.492957746	8.4157	8.3609
155	81.9	12.52063016	1.046273217	0.538461538	8.9427	9.8745
146	67.1	9.308841843	0.988307692	0.574712644	8.7148	8.7148
176	83.0	14.28132678	0.889273118	0.43715847	8.8034	8.9838
167	69.5	12.5748503	0.731619048	0.409090909	9.0077	9.0077
166	105.4	13.23736504	0.732774232	0.45751634	7.7911	7.7911
162	96.3	12.60454002	0.967905213	0.48630137	10.458	10.988

159	66.0	12.45283019	1.212575758	0.459854015	8.7501	8.7501
182	73.6	15.3731685	0.650483991	0.247572816	5.7652	5.7652
156	73.1	12.92041292	1.052597938	0.474820144	8.8076	9.328
170	87.1	15.30147059	0.921480058	0.461538462	9.1213	9.1213
167	70.1	13.80911646	0.702434513	0.384146341	9.4997	9.6692
162	77.2	11.99294533	1.000588235	0.366412214	8.3746	8.3746
162	80.9	12.41830065	0.837473684	0.312056738	9.7657	10.329
164	91.5	13.76412849	0.893627229	0.512820513	8.525	8.4702
158	70.3	13.21729958	0.854940144	0.434482759	7.0623	7.6394
157	102.5	11.99044586	1.167596282	0.472440945	9.938	10.48
153	94.8	12.55091409	0.725046792	0.338461538	7.3175	8.0244
160		0			a	a
142	95.8	9.608334941	0.759757028	0.505747126	5.7177	6.5832
168	98.2	11.98593074	0.675792325	0.469387755	8.5943	8.6625
175	90.3	14.64550265	1.194906069	0.544973545	10.21	10.797
175	75.4	13.72684458	1.209309241	0.542857143	9.8136	10.172
165	91.5	13.18181818	0.523448276	0.387096774	4.7702	4.7702
154	78.6	11.80290298	0.652381877	0.112	4.3901	4.3901
170	85.3	14.40402477	0.888640516	0.220930233	7.6062	8.4953
155	74.2	11.96670135	0.835652174	0.444444444	8.0104	8.6854
173	81.50289	14.27310727	0.805711033	0.360655738	8.7615	9.2917
156	88.46154	8.106255356	0.851194503	0.338028169	5.6156	5.8521
158	104.4304	7.848157585	1.00660568	0.585714286	9.9396	10.345
150	69.33333	7.481717662	0.989077687	0.476923077	4.6604	4.6604
154	98.06452	7.961154761	0.690854551	0.485714286	3.1768	3.783
185	65.40541	8.594092009	0.779605337	0.27027027	0.1224	7.533
155	98.70968	8.157107134	0.723295637	0.472222222	5.4867	5.4867
166	91.61677	8.658243081	1.085670605	0.282608696	9.2537	9.2537
145	73.05389	10.44887356	0.957040961	0.551020408	8.3057	9.092
153	66.66667	11.15357915	0.618635499	0.37398374	a	6.427
167	92.81046	7.972716516	0.877994343	0.348314607	8.8527	9.484
154	90.19608	7.697947546	1.312038039	0.582089552	8.5338	8.8634
150	74.5098	8.013532425	0.935916847	0.5625	6.1164	6.6349
154	87.5817	10.54674781	0.701638091	0.261261261	6.2153	7.155
160	87.5817	8.314801217	1.046326878	0.64556962	7.4607	7.159
150	80.39216	7.281651278	0.933854114	0.4		6.3712
151	87.5817	7.401711652	0.918706418	0.949152542		6.2656
144	69.28105	10.14738677	1.133296706	0.569892473		8.7712
149	87.5817	11.31900278	0.680271942	0.392523364		7.0148
141	64.70588	6.783787962	1.149800089	0.755555556		5.9919
138	85.62092	9.003758059	0.699707828	0.319444444		6.1819
179	114.3791	9.28847134	0.645962051	0.473684211		6.2806
144	81.04575	7.054109246	0.79386352	0.333333333		5.0458

slope_1	slope_2	EELV_rst	EELV_un	EELV_AT	EELV_pk	EELV_rst/TLC
10.155	8.7937	2.92	3.4	2.77	2.86	0.5
5.2683	7.2272	a	a	a	a	a
5.9877	7.6803	a	a	a	a	a
6.7818	9.3628	a	a	a	a	a
6.1604	9.0522	a	a	a	a	a
6.0395	7.0873	a	a	a	a	a
7.7734	9.4052	a	1.82	2.17	2.33	a
4.9607	7.6795	a	2.92	2.38	2.48	a
5.7351	9.6938	a	3.54	3.64	3.81	a
6.6764	9.7084	a	2.79	3.01	3.11	a
7.2801	7.2843	a	2.44	2.68	2.55	a
8.182	8.4877	1.85	1.05	1.44	2.23	0.395299145
8.0276	8.4412	2.14	2.22	1.84	2.54	0.44214876
7.726	6.9622	3.81	4.91	3.11	3.86	0.559471366
3.4044	11.107	4.59	3.56	4.21	3.66	0.659482759
9.2599	9.4878	2.4	2.26	1.66	1.55	0.436363636
6.6732	9.5808	3.36	3.62	3.49	3.46	0.566610455
5.2925	9.0356	3.32	2.27	3.66	2.84	0.615955473
6.6379	8.715	2.48	2.96	3.47	3.28	0.421768707
8.7366	9.0358	2.53	2.36	2.41	2.93	0.439236111
9.8575	9.5269	2.1	1.75	1.48	2.1	0.313432836
7.3247	10.801	1.43	2.01	2.05	2.56	0.278752437
5.3124	10.343	4.78	3.82	3.88	4.03	0.665738162
7.4882	11.698	2.09	1.49	1.99	1.87	0.380692168
9.7359	10.097	2.55	1.8	2.55	2.61	0.383458647
7.83	11.446	a	2.97	2.82	3.25	a
7.0903	12.647	a	3.71	1.71	3.21	a
4.6938	8.726	1.5	2.78	1.74	2.45	0.243902439
6.9761	9.1313	3.39	3.56	3.23	a	0.620879121
7.9368	9.2444	3.54	2.3	2.97	2.8	0.492350487
5.5001	10.498	3.49	3.35	3.42	3.67	0.58263773
6.1319	8.9908	4.62	3.96	4.13	3.96	0.708588957
6.1476	9.465	3.12	2.16	1.63	3.33	0.414342629
8.1576	6.646	3.68	3.06	3.85	3.25	0.600326264
7.0006	11.471	3.13	3.55	3.2	2.38	0.60776699
7.8782	14.029	2.93	2.78	2.84	2.94	0.593117409
7.8507	8.7948	a	2.74	3.19	3.19	a
9.0011	11.098	2.77	2.52	1.17	2.29	0.445337621
5.4327	8.5608	1.15	1.13	1.57	2.32	0.219465649
9.5841	12.439	2.26	2.66	2.53	3.26	0.372937294

6.4835	7.9763	1.98	3	2.9	3.08	0.4125
6.9863	3.9316	2.58	1.73	3.68	2.59	0.449477352
6.6394	9.6852	3.22	3.17	2.22	3.46	0.509493671
7.1321	10.189	3.25	3.21	3.34	3.31	0.477239354
8.2165	8.2797	3.87	a	3.6	4.2	a
4.8702	8.8391	1.54	1.76	1.9	2.22	0.289473684
5.4153	11.513	2.98	2.96	2.75	2.98	0.529307282
9.1928	7.9541	a	2.84	3.06	3.21	a
5.2839	6.95	3.7	3.33	3.83	3.57	0.613598673
7.218	11.147	3.23	3.23	3.08	2.81	0.594843462
5.7279	6.0177	2.84	2.46	2.44	2.6	0.559055118
a	a	a	a	a	a	a
3.4659	8.9158	4.06	2.71	3.51	3.19	0.736842105
8.1368	8.5814	4.07	4.12	3.9	4.43	0.626153846
8.4282	10.305	3.43	2.01	1.91	2.36	0.496382055
8.6335	10.751	2.83	2.33	0.66	0.83	0.521178637
3.3255	4.8726	4.79	4.75	4.75	4.91	0.689208633
5.8651	5.3875	3	3.69	3.28	3.5	0.529100529
3.2668	8.8207	2.73	2.6	2.85	3.19	0.464285714
7.2139	14.098	3.62	2.96	3.19	2.72	0.655797101
6.471	10.198	3.17	2.36	2.19	2.15	0.549393414
0.2266	8.0814	2.03	2.07	2.26	2.03	0.55922865
8.0506	10.255	1.84	0.81	0.99	0.54	0.671532847
3.3081	7.8211	3.61	4.23	4.41	4.32	0.863636364
1.473	4.9674	1.71	1.96	2.03	1.88	0.591695502
3.4357	7.001	a	1.96	1.98	2.15	a
2.6573	7.6719	2.06	1.84	2.1	2.13	0.641744548
1.0653	10.394	1.72	1.63	1.87	1.87	0.412470024
5.3505	10.1	3.24	2.94	2.84	3.44	0.554794521
a	a	a	a	a	a	a
4.465	10.497	3.06	2.62	2.84	2.62	0.798955614
3.2365	13.123	2.61	2.61	3.35	-	0.649253731
1.1697	16.791	?	?	2.31	2.3	?
0.4021	8.4136	?	?	?	2.47	?
5.4931	8.1619	2.85	2.39	2.36	2.18	0.47979798
		1.49	1.39	missed	1.56	0.532142857
		1.5	1.15	0.98	0.98	0.461538462
		2.39	2.53	2.25	1.83	0.56501182
		2	1.96	1.66	1.66	0.549450549
		2.48	2.12	2.48	2.32	0.644155844
		2.16	1.98	2.11	2.08	0.620689655
		2.68	2.92	2.66	2.89	0.623255814
		1.49	1.31	1.55	1.32	0.653508772

EELV_un/TLC	EELV_AT/TLC	EELV_pk/TLC	VTpk/TLC	ICpk(tlc-DU)
0.582191781	0.474315068	0.489726027	0.42859589	2.98
a	a	a	0.348670757	a
a	a	a	0.35320122	a
a	a	a	0.405292479	a
a	a	a	0.319965278	a
a	a	a	0.341860465	a
0.37295082	0.444672131	0.477459016	0.33442623	2.55
0.480263158	0.391447368	0.407894737	0.342763158	3.6
0.515283843	0.529839884	0.554585153	0.303202329	3.06
0.43525741	0.469578783	0.485179407	0.328393136	3.3
0.502057613	0.551440329	0.524691358	0.397530864	2.31
0.224358974	0.307692308	0.476495726	0.388034188	2.45
0.458677686	0.380165289	0.524793388	0.309917355	2.3
0.720998532	0.456681351	0.56681351	0.281204112	2.95
0.511494253	0.604885057	0.525862069	0.38591954	3.3
0.410909091	0.301818182	0.281818182	0.288	3.95
0.610455312	0.588532884	0.583473862	0.331703204	2.47
0.421150278	0.67903525	0.52690167	0.229313544	2.55
0.503401361	0.590136054	0.557823129	0.311564626	2.6
0.409722222	0.418402778	0.508680556	0.274479167	2.83
0.26119403	0.220895522	0.313432836	0.4	4.6
0.391812865	0.399610136	0.499025341	0.297660819	2.57
0.532033426	0.540389972	0.561281337	0.304178273	3.15
0.27140255	0.362477231	0.340619308	0.322586521	3.62
0.270676692	0.383458647	0.392481203	0.306315789	4.04
0.507692308	0.482051282	0.555555556	0.325982906	2.6
0.514563107	0.237170596	0.445214979	0.255617198	4
0.45203252	0.282926829	0.398373984	0.312682927	3.7
0.652014652	0.591575092	a	0.262271062	a
0.319888734	0.413073713	0.389429764	0.457858136	4.39
0.559265442	0.570951586	0.612687813	0.305843072	2.32
0.607361963	0.633435583	0.607361963	0.302760736	2.56
0.28685259	0.216467463	0.442231076	0.342231076	4.2
0.499184339	0.628058728	0.530179445	0.331158238	2.88
0.689320388	0.621359223	0.462135922	0.298640777	2.77
0.562753036	0.574898785	0.5951417	0.220647773	2
0.446254072	0.519543974	0.519543974	0.302605863	2.95
0.405144695	0.188102894	0.368167203	0.244533762	3.93
0.215648855	0.299618321	0.442748092	0.41889313	2.92
0.438943894	0.417491749	0.537953795	0.305445545	2.8

0.625	0.604166667	0.641666667	0.296458333	1.72
0.301393728	0.641114983	0.451219512	0.381184669	3.15
0.501582278	0.351265823	0.547468354	0.335601266	2.86
0.471365639	0.490455213	0.486049927	0.341409692	3.5
a	a	a	a	a
0.330827068	0.357142857	0.417293233	0.528571429	3.1
0.525754885	0.488454707	0.529307282	0.336234458	2.65
0.455128205	0.490384615	0.514423077	0.302884615	3.03
0.552238806	0.635157546	0.592039801	0.295190713	2.46
0.594843462	0.567219153	0.517495396	0.384162063	2.62
0.484251969	0.480314961	0.511811024	0.304527559	2.48
a	a	a	a	a
0.491833031	0.637023593	0.578947368	0.285662432	2.32
0.633846154	0.6	0.681538462	0.287538462	2.07
0.290882779	0.276410999	0.341534009	0.443560058	4.55
0.429097606	0.121546961	0.152854512	0.370165746	4.6
0.683453237	0.683453237	0.70647482	0.17971223	2.04
0.650793651	0.578483245	0.617283951	0.224338624	2.17
0.442176871	0.484693878	0.542517007	0.333503401	2.69
0.536231884	0.577898551	0.492753623	0.281884058	2.8
0.409012132	0.379549393	0.372616984	0.29982669	3.62
0.570247934	0.622589532	0.55922865	0.386225895	1.6
0.295620438	0.361313869	0.197080292	0.332846715	2.2
1.011961722	1.055023923	1.033492823	0.234449761	-0.14
0.678200692	0.702422145	0.650519031	0.263321799	1.01
0.620253165	0.626582278	0.680379747	0.17943038	1.01
0.573208723	0.654205607	0.663551402	0.24953271	1.08
0.39088729	0.448441247	0.448441247	0.360911271	2.3
0.503424658	0.48630137	0.589041096	0.24109589	2.4
a	a	a	0.22097561	4.1
0.684073107	0.74151436	0.684073107	0.247780679	1.21
0.649253731	0.833333333	?	0.310447761	?
?	0.417721519	0.415913201	0.239240506	3.23
?	?	0.446654611	0.358589512	3.06
0.402356902	0.397306397	0.367003367	0.225925926	3.76
0.496428571	?	0.557142857	0.3	1.24
0.353846154	0.301538462	0.301538462	0.291692308	2.27
0.598108747	0.531914894	0.432624113	0.294799054	2.4
0.538461538	0.456043956	0.456043956	0.342032967	1.98
0.550649351	0.644155844	0.602597403	0.226753247	1.53
0.568965517	0.606321839	0.597701149	0.304022989	1.4
0.679069767	0.618604651	0.672093023	0.191627907	1.41
0.574561404	0.679824561	0.578947368	0.337280702	0.96

O'D thr (EB-DC)	O'D ratio DC/EB	MVV	pulse_pressure_rest
0.477	0.839932886	131.6	36
a	a	84.9	63
a	a	150.8	35
a	a	139.1	54
a	a	117.0	52
a	a	111.0	35
0.918	0.64	116.7	45
1.516	0.578888889	93.8	57
0.977	0.680718954	116.1	69
1.195	0.637878788	108.0	39
0.378	0.836363636	88.2	84
0.634	0.74122449	100.0	58
0.8	0.652173913	93.2	81
1.035	0.649152542	128.0	47
0.614	0.813939394	104.3	79
2.366	0.401012658	100.0	58
0.503	0.796356275	100.0	45
1.314	0.484705882	104.3	56
0.768	0.704615385	117.0	81
1.249	0.558657244	112.8	68
1.92	0.582608696	128.9	48
1.043	0.594163424	87.2	55
0.966	0.693333333	113.7	64
1.849	0.489226519	131.9	64
2.003	0.504207921	145.7	28
0.693	0.733461538	119.6	43
2.157	0.46075	146.7	56
1.777	0.51972973	115.9	55
a	a	107.3	31
1.098	0.749886105	160.3	56
0.488	0.789655172	122.1	52
0.586	0.77109375	135.3	37
1.623	0.613571429	169.2	55
0.85	0.704861111	124.4	70
1.232	0.555234657	76.9	53
0.91	0.545	61.2	
1.092	0.629830508	125.9	50
2.409	0.387022901	122.0	51
0.725	0.751712329	115.2	94
0.949	0.661071429	130.8	30

0.297	0.827325581	92.2	84
0.962	0.694603175	129.3	32
0.739	0.741608392	112.1	42
1.175	0.664285714	138.2	15
a	a	151.7	36
0.288	0.907096774	114.3	60
0.757	0.714339623	100.0	26
1.14	0.623762376	123.7	44
0.68	0.723577236	95.8	19
0.534	0.796183206	90.8	61
0.933	0.623790323	97.9	84
a	a	a	a
0.746	0.678448276	91.8	70
0.201	0.902898551	128.2	30
1.485	0.673626374	155.4	49
2.59	0.436956522	93.9	54
0.791	0.612254902	110.5	91
0.898	0.586175115	69.9	69
0.729	0.728996283	114.9	35
1.244	0.555714286	102.0	74
1.89	0.477900552	117.8082192	27
0.198	0.87625	57.57575758	78
1.288	0.414545455	52.05479452	60
-1.12	-7	86.84210526	69
0.249	0.753465347	47.27272727	60
0.443	0.561386139	50	83
0.279	0.741666667	57.33333333	47
0.795	0.654347826	73.03370787	28
0.992	0.586666667	113.6363636	55
3.194	0.22097561	76.31578947	46
0.261	0.784297521	58.82352941	60
?	?	41.57303371	78
1.907	0.409597523	80.95238095	66
1.077	0.648039216	84.21052632	83
2.418	0.356914894	79.74683544	69
0.4	0.677419355	49.27536232	56
1.322	0.417621145	56.60377358	62
1.153	0.519583333	72.22222222	55
0.735	0.628787879	?	60
0.657	0.570588235	57.8125	#VALUE!
0.342	0.755714286	55.12820513	#VALUE!
0.586	0.584397163	68.96551724	29
0.191	0.801041667	45.16129032	64

pulse_pressure_un	pulse_pressure_AT	pulse_pressure_PK	VTrst/TLC	Vtunl/TLC
36	41	34	0.146575342	0.15
82	76	85	0.199795501	0.174437628
30	69	55	0.111890244	0.122713415
54	85	131	0.126740947	0.167966574
52	73	120	0.116145833	0.143923611
35	35	123	0.13255814	0.130232558
58	59	64	0.103278689	0.142008197
94	99	144	0.168914474	0.190296053
69	125	154	0.077292576	0.122125182
31	90	43	0.120592824	0.170202808
92	45	45	0.20473251	0.214814815
58	89	109	0.108547009	0.17457265
81	86	118	0.383057851	0.181404959
47	66	103	0.115859031	0.125844347
79	127	136	0.126436782	0.16091954
63	75	122	0.090727273	0.135818182
66	53	104	0.132209106	0.171332209
56	72	85	0.115769944	0.149536178
88	77	116	0.050510204	0.126190476
68	105	97	0.109895833	0.139756944
48	41	61	0.105223881	0.104626866
55	106	90	0.159844055	0.155945419
64	79	137	0.070194986	0.175626741
68	85	122	0.13132969	0.167395264
49	59	90	0.175338346	0.156842105
43	78	112	0.084273504	0.127521368
56	88	113	0.112760055	0.223162275
55	88	122	0.163252033	0.131869919
31	70	95	0.081868132	0.131684982
67	93	165	0.097357441	0.08038943
52	63	83	0.083472454	0.075125209
37	65	97	0.083435583	0.125766871
55	74	131	0.385258964	0.243426295
70	78	94	0.118923328	0.176182708
53	68	75	0.116699029	0.17592233
20	28	34	0.160526316	0.229554656
50	78	115	0.125570033	0.168241042
46	50	62	0.145176849	0.311414791
49	70	91	0.14370229	0.441030534
28	20	62	0.09290429	0.124092409

84	104	82	0.179375	0.156875
13	13	23	0.131707317	0.225958188
42	35	58	0.112658228	0.155221519
15	36	85	0.102496329	0.045080764
36	39	50	a	a
59	59	17	0.222744361	0.252067669
50	55	73	0.106927176	0.160213144
46	19	54	0.081410256	0.130128205
58	62	70	0.200331675	0.144278607
77	79	60	0.131491713	0.181767956
84	126	128	0.136023622	0.158464567
a	a	a	a	a
70	100	36	0.117059891	0.196370236
30	95	76	0.104	0.121384615
34	37	40	0.102315485	0.419392185
67	67	70	0.206629834	0.236279926
91	81	115	0.10705036	0.152661871
69	86	91	0.108112875	0.196119929
58	58	101	0.08707483	0.133673469
74	102	102	0.230434783	0.209963768
45	14	41	0.169844021	0.204159445
78	110	122	0.190633609	0.219834711
60	98	121	0.159124088	0.232116788
69	66	81	0.150717703	0.183971292
60	76	80	0.159861592	0.203114187
83	55	59	0.094620253	0.132594937
59	74	87	0.126791277	0.166666667
28	31	78	0.176738609	0.240527578
55	85	92	0.128082192	0.154280822
46	52	63	0.114390244	0.185365854
46	64	91	0.171801567	0.201044386
78	37	46	0.119900498	0.124875622
66	67	46	0.087884268	0.167450271
83	79	121	0.174683544	0.246112116
69	81	23	0.114478114	0.148821549
56	70	70	0.153928571	0.192857143
58	94	94	0.188307692	0.281230769
55	75	128	0.173995272	0.142553191
60	128	128	0.159340659	0.246703297
93	99	94	0.215584416	0.284155844
118	115	56	0.118103448	0.229022989
29	66	92	0.046976744	0.087674419
73	77	96	0.190789474	0.275877193

VTAT/TLC	dLeg_dVO2	dSOB_dVO2	dHR_dVO2	dLeg_dVO2_	dSOB_dVO2_
0.382191781	0.0021	0.002	0.0356	0.0549	0.052
0.246830266	0.0066	0.006	0.0603	0.1147	0.1048
0.219817073	0.0063	0.0059	0.0604	0.1692	0.1598
0.325487465	0.0046	0.0035	0.0329	0.1021	0.0786
0.208680556	0.0066	0.0071	0.1049	0.1096	0.1192
0.166666667	0.0039	0.0041	0.0429	0.0698	0.0723
0.217213115	0.0088	0.01	0.0488	0.1534	0.1754
0.299177632	0.0044	0.0044	0.0582	0.0858	0.0858
0.156331878	0.0065	0.0054	0.0546	0.1029	0.0858
0.230889236	0.0066	0.0049	0.0764	0.1039	0.0775
0.281481481	0.0095	0.0093	0.0587	0.1404	0.1371
0.257264957	0.0047	0.0037	0.0475	0.0861	0.067
0.244421488	0.0036	0.0035	0.0401	0.0716	0.0711
0.214390602	0.0044	0.0037	0.0609	0.0896	0.0744
0.165517241	0.0058	0.0036	0.0623	0.1001	0.0616
0.204	0.004	0.0033	0.0531	0.0584	0.0478
0.164586847	0.0101	0.008	0.0704	0.1964	0.1558
0.177736549	0.0075	0.0052	0.0727	0.1299	0.0896
0.159013605	0.0035	0.0024	0.0559	0.0572	0.0385
0.194791667	0.0049	0.0041	0.0345	0.0885	0.0736
0.214776119	0.0039	0.0038	0.0392	0.0873	0.0849
0.321247563	0.0059	0.006	0.0685	0.0893	0.0904
0.195821727	0.0064	0.0054	0.0553	0.1172	0.1001
0.449908925	0.0023	0.0019	0.0345	0.0487	0.0403
0.379699248	0.0061	0.0054	0.0478	0.1288	0.1151
0.180854701	0.0024	0.0024	0.0443	0.0478	0.0478
0.194868239	0.0078	0.007	0.0595	0.1562	0.1393
0.188455285	0.0039	0.0039	0.0817	0.0693	0.0693
0.165750916	0.0054	0.0054	0.0357	0.1014	0.1014
0.256328234	0.0035	0.0032	0.0367	0.0793	0.0722
0.162771285	0.0034	0.0032	0.0426	0.0757	0.0699
0.176380368	0.0026	0.0021	0.0812	0.0506	0.0418
0.358034529	0.0045	0.0044	0.0551	0.1183	0.1143
0.22822186	0.0027	0.0021	0.0649	0.0568	0.0452
0.229514563	0.0049	0.0042	0.0515	0.0966	0.0819
0.189473684	0.023	0.0066	0.0263	0.3107	0.0888
0.247068404	0.0072	0.0066	0.0409	0.1817	0.1667
0.621382637	0.0085	0.0028	0.035	0.1772	0.0583
0.191412214	0.0071	0.0066	0.0646	0.1559	0.1454
0.377887789	0.0028	0.0025	0.0512	0.0581	0.052

0.22875	0.0071	0.0062	0.0438	0.14	0.1228
0.140766551	0.0112	0.0063	0.0477	0.3178	0.1791
0.173101266	0.0065	0.0069	0.0635	0.1315	0.1382
0.208957416	0.004	0.0033	0.0362	0.1047	0.0845
a	0.01	0.0104	0.0304	0.2292	0.2375
0.233646617	0.0077	0.007	0.049	0.1488	0.1361
0.294849023	0.0028	0.0028	0.0421	0.0561	0.057
0.188301282	0.0063	0.006	0.0544	0.141	0.1346
0.200497512	0.0072	0.0072	0.0531	0.1496	0.1496
0.237016575	0.0039	0.0034	0.0339	0.0739	0.0645
0.228149606	0.0049	0.0046	0.0702	0.0936	0.0872
a	a	a	a	a	a
0.186569873	0.0073	0.0069	0.0406	0.1	0.0933
0.175230769	0.0036	0.0031	0.0783	0.0731	0.0622
0.461794501	0.0024	0.0021	0.0406	0.0606	0.0529
0.270718232	0.0018	0.0018	0.0313	0.0435	0.0435
0.130215827	0.0088	0.0087	0.0874	0.1916	0.189
0.185185185	0.0115	0.0049	0.0702	0.208	0.0885
0.169897959	0.0043	0.0031	0.0492	0.1046	0.0763
0.255615942	0.0053	0.0046	0.0298	0.0962	0.0847
0.255632582					
0.21184573					
0.278467153					
0.25					
0.194463668					
0.147151899					
0.186915888					
0.165707434					
0.183561644					
0.222682927					
0.252741514					
0.203731343					
0.198372514					
0.251537071					
0.189225589					
0.214642857					
0.288615385					
0.186524823					
0.263736264					
0.219220779					
0.247988506					
0.16744186					
0.278508772					

dHR_dVO2_	dLeg_dVO2_kg	dSOB_dVO2_kg	dHR_dVO2_kg	dLeg_dVO2,L_
0.9276	0.1523	0.1442	2.5628	2.1
1.0536	0.4948	0.4517	4.5754	6.6
1.6204	0.4716	0.4452	4.5259	6.3
0.7291	0.3857	0.2959	2.7695	4.6
1.7652	0.3679	0.3995	5.8718	6.6
0.764	0.2799	0.2902	3.0495	3.9
0.8513	0.5346	0.6105	2.9721	8.8
1.1731	0.3529	0.3529	4.7181	4.4
0.8592	0.4863	0.4059	4.0917	6.5
1.2124	0.4453	0.3324	5.1959	6.6
0.8696	0.7105	0.6937	4.4056	9.5
0.8627	0.3168	0.2467	3.18	4.7
0.8073	0.2866	0.2849	3.2069	3.6
1.2263	0.3473	0.2883	4.76	4.4
1.0708	0.3579	0.2207	3.8546	5.8
0.785	0.2187	0.1789	2.9237	4
1.3557	0.6401	0.5078	4.4374	10.1
1.2533	0.479	0.3318	4.658	7.5
0.9098	0.2299	0.1555	3.6361	3.5
0.6221	0.3126	0.2601	2.2062	4.9
0.8834	0.3056	0.2974	3.0972	3.9
1.0407	0.3061	0.3096	3.5644	5.9
1.0247	0.4271	0.3646	3.7098	6.4
0.7229	0.1751	0.1449	2.589	2.3
1.0134	0.4198	0.3752	3.2968	6.1
0.8709	0.1453	0.1453	2.6564	2.4
1.1904	0.4436	0.3956	3.3877	7.8
1.424	0.2408	0.2408	4.9851	3.9
0.6626	0.4034	0.4034	2.6746	5.4
0.8226	0.2495	0.2271	2.6039	3.5
0.9403	0.2711	0.2505	3.3654	3.4
1.5953	0.1453	0.1199	4.5504	2.6
1.4344	0.3714	0.359	4.5163	4.5
1.3609	0.2151	0.1697	5.1989	2.7
1.0033	0.382	0.324	4.0231	4.9
0.3577	1.261	0.3603	1.4469	23
1.0247	0.5826	0.5357	3.3183	7.2
0.7345	0.5067	0.1681	2.1026	8.5
1.426	0.5654	0.5267	5.1767	7.1
1.0392	0.1872	0.1673	3.3759	2.8

0.8696	0.5165	0.4529	3.199	7.1
1.3292	0.9796	0.5523	4.1633	11.2
1.2761	0.4926	0.5173	4.7655	6.5
0.9363	0.3915	0.3158	3.511	4
0.7081	0.8321	0.8631	2.5201	10
0.9531	0.5954	0.5439	3.7746	7.7
0.8471	0.1972	0.2003	2.9448	2.8
1.2252	0.5326	0.509	4.6259	6.3
1.1118	0.5617	0.5617	4.1546	7.2
0.6389	0.2817	0.246	2.4392	3.9
1.3502	0.3619	0.3371	5.1724	4.9
a	a	a	a	a
0.5554	0.4028	0.3759	2.2277	7.3
1.5695	0.2195	0.1868	4.7015	3.6
1.041	0.1915	0.1672	3.2877	2.4
0.7523	0.1395	0.1395	2.4111	1.8
1.8971	0.6209	0.614	6.2159	8.8
1.2744	0.7769	0.3294	4.7647	11.5
1.2039	0.384	0.2789	4.3833	4.3
0.5484	0.3653	0.3213	2.0549	5.3

dSOB_dVO2,L_	dHR_dVO2,L_	dLeg_dVO2_*100_	dSOB_dVO2_*100_
2	35.6	5.49	5.2
6	60.3	11.47	10.48
5.9	60.4	16.92	15.98
3.5	32.9	10.21	7.86
7.1	104.9	10.96	11.92
4.1	42.9	6.98	7.23
10	48.8	15.34	17.54
4.4	58.2	8.58	8.58
5.4	54.6	10.29	8.58
4.9	76.4	10.39	7.75
9.3	58.7	14.04	13.71
3.7	47.5	8.61	6.7
3.5	40.1	7.16	7.11
3.7	60.9	8.96	7.44
3.6	62.3	10.01	6.16
3.3	53.1	5.84	4.78
8	70.4	19.64	15.58
5.2	72.7	12.99	8.96
2.4	55.9	5.72	3.85
4.1	34.5	8.85	7.36
3.8	39.2	8.73	8.49
6	68.5	8.93	9.04
5.4	55.3	11.72	10.01
1.9	34.5	4.87	4.03
5.4	47.8	12.88	11.51
2.4	44.3	4.78	4.78
7	59.5	15.62	13.93
3.9	81.7	6.93	6.93
5.4	35.7	10.14	10.14
3.2	36.7	7.93	7.22
3.2	42.6	7.57	6.99
2.1	81.2	5.06	4.18
4.4	55.1	11.83	11.43
2.1	64.9	5.68	4.52
4.2	51.5	9.66	8.19
6.6	26.3	31.07	8.88
6.6	40.9	18.17	16.67
2.8	35	17.72	5.83
6.6	64.6	15.59	14.54
2.5	51.2	5.81	5.2

6.2	43.8	14	12.28
6.3	47.7	31.78	17.91
6.9	63.5	13.15	13.82
3.3	36.2	10.47	8.45
10.4	30.4	22.92	23.75
7	49	14.88	13.61
2.8	42.1	5.61	5.7
6	54.4	14.1	13.46
7.2	53.1	14.96	14.96
3.4	33.9	7.39	6.45
4.6	70.2	9.36	8.72
a	a	a	a
6.9	40.6	10	9.33
3.1	78.3	7.31	6.22
2.1	40.6	6.06	5.29
1.8	31.3	4.35	4.35
8.7	87.4	19.16	18.9
4.9	70.2	20.8	8.85
3.1	49.2	10.46	7.63
4.6	29.8	9.62	8.47

dHR_dVO2_*100_	心超與CEPT日期計算	Obtained date_2D Echo (Y/M/D)	mIVSd, cm	mLVIDd, cm
92.76				
105.36				
162.04				
72.91				
176.52				
76.4				
85.13				
117.31				
85.92				
121.24				
86.96				
86.27				
80.73				
122.63				
107.08				
78.5				
135.57				
125.33				
90.98				
62.21				
88.34				
104.07				
102.47				
72.29				
101.34				
87.09	0	6/14/2019	0.89	4.87
119.04	0	6/19/2019	0.94	4.71
142.4	0	6/21/2019	1.02	4.79
66.26	0	7/17/2019	1.6	4.92
82.26	0	7/12/2019	1.07	4.88
94.03	0	7/12/2019	0.96	5.17
159.53	0	8/7/2019	0.97	4.76
143.44	0	8/21/2019	1.1	4.03
136.09	6	4/24/2018	1.14	6.35
100.33	23	3/26/2018	0.91	5.38
35.77	133	1/17/2018	1.04	5.01
102.47	49	7/24/2018	0.88	5.11
73.45	52	6/29/2018	0.88	4.71
142.6	2	6/1/2018	0.95	4.89
103.92	43	4/2/2018	0.93	5.2

86.96	88	3/2/2018	1.06	5.44
132.92	15	5/1/2018	1.04	4.86
127.61	43	5/7/2018	1.03	5.26
93.63	53	4/27/2018	1.22	6.74
70.81	23	6/27/2018	0.97	6.68
95.31	50	7/16/2018	0.86	5.3
84.71	44	7/23/2018	0.96	5.96
122.52	40	8/12/2018	1.22	4.4
111.18	310	12/27/2017	0.97	6.03
63.89	21	10/31/2018	1.2	5.06
135.02	0	10/16/2019	0.86	5.76
a	a	1/16/2020	1.15	6.64
55.54	41	2/27/2020	1.01	5.09
156.95	365	4/25/2019	0.88	4.47
104.1	79	2/17/2020	1.02	5.04
75.23	7	5/22/2020	1.21	5.86
189.71	6	6/4/2020	1.26	6.78
127.44	0	6/17/2020	1.68	5.78
120.39	0	7/3/2020	1.39	7.33
54.84	0	7/8/2020	1.22	5.68
		2/12/2020	1.18	4.84
		5/22/2020	1.21	3.76
		12/29/2021		
		10/14/2020	0.97	4.69
		6/12/2020	1.03	5.19
		8/7/2020	0.87	4.56
		8/18/2020	0.99	4.77
		9/30/2020	0.89	4.01
		10/7/2020	0.97	4.83
		10/14/2020	1.06	5.12
		12/1/2020	1.1	4.39
		1/10/2022		
		1/23/2021	1.08	5.26
		1/14/2021	1.28	4.69
		5/12/2021	1.52	4.7
		10/1/2021	1.25	5.33
		7/30/2021	1.26	4.88
		9/6/2021	1.02	5.53
		3/15/2021	1.26	5.02
		11/11/2021	1	5.59
		9/29/2021	1.19	4.83
		2/19/2019	1.19	3.92

LVPWd	MV E/A	IVSs, cm	LVIDs, cm	LVPWs	LVOTD, cm	AoRD, cm	Max vel_TR, cm/s
0.97	1	1.29	3.44	1.32	2.09	3.1	223
0.98	1	1.35	3.15	1.56	2.34	3.4	260
1.02	0.7	1.27	3.28	1.56	2.27	3.6	238
1.39	0.6	1.76	3.36	1.84	2.37	3.5	221
0.95	1.1	1.45	3.05	1.56	2.51	3.2	257
1	0.8	1.48	3	1.61	2.19	3.5	244
1.07	1.4	1.36	3.36	1.5	2.37	2.8	237
0.93	1.3	1.4	2.83	1.13	1.9	3.2	143
0.89	0.6	1.67	4.43	1.34	2	3.5	225
1.01	0.6	1.39	4.18	1.45	2.3	3.1	236
1.07	0.6	1.29	3.9	1.22	2.1	3.9	197
1.03	1.1	1.33	3.98	1.37	2.3	3	234
0.95	0.7	1.22	3.9	1.32	2.2	3.1	239
0.89	0.8	1.08	3.56	1.43	2.2	3.2	276
0.93	0.6	1.29	3.8	1.4	2.2	3.3	218

1.03	0.7	1.31	4.19	1.16	2.4	3.1	242
0.96	0.7	1.13	3.82	1.48	2.7	3.2	236
0.99	0.7	1.45	4.04	1.41	2.2	3.1	260
1.35	0.8	1.39	5.91	1.61	2.41	3.2	229
1.02	0.6	1.31	5.45	1.4	2.2	2.8	287
0.88	1.1	1.15	4.08	1.54	2.2	3	250
1.04	0.8	1.33	5	1.37	2.5	2.9	227
1.22	1.48	1.41	3.54	1.45	2.16	3.49	242
1.02		1.46	5.2	1.6	2.3	3.4	380
1.22	0.5	1.47	3.91	1.64	2.31	3	268
1.04	0.7	1.29	4.33	1.57	2.22	3.5	300
1.19	1.4	1.43	5.56	1.69	2.37	3.2	229
1.05	0.7	1.2	4.02	1.68	2.43	3.4	234
1.01	1.4	1.21	3.51	1.25	1.71	3.14	212
1.02	1.1	1.31	3.85	1.46	2.34	3.1	257
1.23	1.4	1.42	4.59	1.76	2	3.1	252
1.09		1.43	4.82	1.35	2.5	3.3	228
1.6	1.3	1.8	4.87	1.8	2	3	233
1.39	0.7	1.63	6.4	1.97	2.27	3.6	269
1.07	0.8	1.41	4.46	1.56	2.04	2.8	271
1.18	1.60	1.75	2.94	1.64	2.13	3.20	297.00
0.86	0.60	1.40	2.58	1.04	1.80	2.70	195.00
1.04	0.7	1.15	3.15	1.57	2.04	3.1	304
1.07	0.8	1.33	3.66	1.53	2.07	2.7	266
0.8	0.8	1.1	3.53	0.97	1.7	2.4	260
0.92	0.6	1.3	3.24	1.49	2.02	3.1	293
0.93	1	1.29	2.61	1.5	2	2.7	244
0.86	0.9	1.11	2.9	1.04	2.1	3.4	244
0.8	0.8	1.23	3.73	1.06	1.7	3.2	261
1	1.5	1.36	3.03	1.46	2.07	3.1	246
0.92	1.1	1.54	3.46	1.15	1.8	2.9	248
1.24	0.7	1.47	3.62	1.22	1.9	2.8	308
1.43	0.6	2.52	3.04	1.83	2.27	3.3	290
0.79	0.6	1.32	3.04	1.11	1.9	2.9	237
1.12	0.7	1.38	3.01	1.3	1.5	2.6	271
1.02	0.6	1.39	3.77	1.8	1.99	3.3	237
0.85	0.8	1.46	3.7	1.22	1.9	2.8	307
0.88	0.5	1.23	3.92	1.12	2	3.3	329
1.19	0.4	1.31	3.4	1.64	2.12	3.2	390
0.92	0.6	1.46	2.46	1.31	1.7	2.8	366

ACS, cm	Max PG_TR, mmHg	mLAD, cm	RA_p. mmHg	EF_T, %	RVSP(TR), mmHg	PA_Vmax, cm/s	LVOT_mx, cm_s
1.5	20	3.6	5	56	25	77.6	106
1.4	27.04	3.8	10	61.7	37.04		86.2
1.7	23	3.1	10	59.3	33	84.9	99
1.7	20	4.3	5	59.6	25	76.2	95.6
1.3	26	4	10	67.5	36	116	118
1.5	24	4	5	72.7	29	105	105
1.4	22	3.5	10	56.1	32	70.8	90.7
2.2	8	3.1	5	57.5	13	97	82
1.8	20	3.97	5	44.5	25	132	72
1.2	22	4.3	10	44.5	32	84.6	72
2.2	16	2.9	5	44.6	16	93.3	111
1.5	27	3.6	5	44	27	70	68.1
1.5	23	3.7	10	46.4	33	62.7	54.5
1.5	30	3.9	10	49	40	67.1	53.5
1.4	19	3.9	10	49	29	86.6	78.3

1.1	23	4.2	5	45.8	28	90.9	74.9
1.3	22	3.5	5	43.5	27	71.8	73.7
1.6	27.04	4	10	46.1	37.04	86.6	106
2.1	21	5.1	10	25.6	31	76.2	80.1
1.3	33	4.6	10	37.5	43	138	88.5
1.2	25	4	10	45	35	65.2	71
1.5	21	3.9	10	33	31	109	41.8
1.81	23.4	3.63	15	42	38.4	70.8	103
1.9	22.47	5.9	15	28.6	73	53	39.2
1.4	29	3.8	5	45.7	34	88.8	77.6
1.7	36	4.4	10	48	46	98.1	95.6
1.5	21	4.6	10	35.1	31	56.8	83.4
1.7	22	3.8	10	42.4	32	72.3	74.3
1.27	18	2.76	5	43.9	23	92.4	78.6
1.7	26.42	4.3	5	46.8	31.42	102	103
1.8	25	4.5	15	43.4	40	88.3	111
2.3	21	4.02	10	44.3	31	98.1	86.8
1.7	22	4.6	10	32.7	32	60.6	66.5
1.9	29	4.6	10	28.6	39	63.1	70.2
1.3	29	4.7	5	43.1	34	83.9	69.4
1.90	15.21	3.60		69.70	47		
2.00		3.00		60.10	38.3		
	37				32.04		
1.4	28.3	3.6	10	61.4	39	88.3	89.7
1.4	27	4	10	56.1	29	69.4	131
1.7	34	3.4	5	56.2	29	101	87.6
1.8	24	3.9	5	60.2	32	109	88.3
1.2	24	3.5	5	64.8	34	87.3	82.7
1.9	27	2.5	5	70.5			97.1
2	24	2.8	5	57.8	30	65	78.3
1.4		3.5	10	58.8	42.95	61.1	103
	25				39		
1.8	37.95	3.7	5	62.8	27	113	109
1.9	34	2.7	5	51.2	34	71	75.4
1.6	22	4.4	5	64.5	32	88.8	197
1.8	29	4.02	5	73.6	48	121	119
2	22	3	5	68.5	53.3	109	81.7
1.5	38	4.4	10	59.2	71	80	117
1.7	43.3	5	10	65.4		132	97
1.8	61	4.3	10	60.3	74	121	142
1.2		4.8	10	56.5		68.9	122
	54						
1.8		4.09	20	67.9		93.3	70.9

PA_Max PG, mmHg	AV_max, cm_s	AVmaxPG, mmHg	AVA, cm^2	LV_mass, g	Obtained date_NM (Y/M/D)
					11/6/2017
0	115	5	3.16	158	6/14/2019
0	101	4	3.67	157	6/19/2019
0	115	5	3.49	174	6/21/2019
0	126	6	3.35	313	7/17/2019
0	148	9	3.95	178	7/12/2019
0	163	11	2.43	187	7/12/2019
0	105	4	3.81	172	8/7/2019
0	119	6	1.96	131	8/21/2019
7	132	7	1.71	278	省略，直接做導管
3	124	6	2.41	195	4/19/2018
3.48	370	55	1.04	196	拒絕
2	96.6	4	2.93	177	6/15/2018
2	91.2	3	2.27	146	5/30/2018
2	131	7	1.55	157	6/20/2018
3	122	6	2.44	142	5/30/2018

3.31	129	7	2.62	221	6/1/2018
2	126	6	3.35	173	5/28/2018
3	113	5	3.56	201	7/6/2018
2	100	4	3.65	427	7/9/2018
8	128	7	2.63	293	8/7/2018
2	124	6	2.18	167	9/11/2018
5	109	5	1.88	221	9/11/2018
2	127	7	2.97		10/3/2018
1.12	88	3	1.85	248	12/26/2018
3	139	8	2.34	241	12/4/2018
0	139	8	2.66	215	10/16/2019
0	117	5	3.14	358	脊椎會痛無法做
0	95.2	4	3.62	195	4/8/2020
0	141	7.99	1.28		4/24/2020
0	143	8	3.1	226	5/6/2020
0	140	8	2.49	309	5/15/2020
0	120	6	3.55	374	5/29/2020
0	97.7	4	2.14	459	6/17/2020
0	107	5	2.66	533	核醫未做
0	129	7	1.76	269	7/8/2020
0	138	8	2.13	165	
0	141	8	3.13	207	
0	145	8	1.37	122	
0	124	6	2.28	158	
0	129	7	2.01	112	
0	113	5.11	2.98	152	
0	129	7	1.38	142	
0	130	7	2.67	157	
0	139	8	1.99	198	
0	104	4		203	
0	125		6.38	308	
0	184	14	1.84	207	
0	159	10	0.91	220	
0	136	7	2.68	221	
0	186	14	1.48	252	
6	192	15	2.32	217	
0	165	11	2.61	219	
0	123	6	1.31	133	

RVEF,%	LVEF,%	RSBI_rs	RSBI_un	RSBI_AT	RSBI_pk	RERRS
71	71.5	14.0	13.7	10.3	16.0	0.680107527
		14.3	15.2	14.9	21.7	1.254237288
		21.8	23.6	16.6	17.3	0.896946565
		11.0	12.4	6.4	10.3	0.81779661
		29.9	22.9	18.3	17.9	0.838461538
		21.1	21.4	24.2	16.8	0.965217391
		39.7	20.2	21.7	23.9	0.813868613
		11.7	19.9	11.0	13.9	0.899109792
		41.4	25.0	27.0	16.8	0.864285714
		22.0	15.6	13.5	18.5	0.750788644
		14.1	21.1	18.3	18.1	0.948051948
		43.3	28.2	21.6	19.3	1.123595506
		4.3	23.9	19.4	18.0	1.032876712
		13.9	18.7	12.3	17.8	0.943089431
		18.2	13.4	17.4	6.7	1.025396825
		36.1	18.7	19.6	18.9	0.602230483
		19.1	15.7	28.7	14.7	0.77037037
		30.4	22.3	25.1	31.6	0.718670077
		57.2	21.6	24.6	16.4	0.674603175
		22.1	26.1	23.2	35.4	0.691823899
		12.8	17.1	13.9	7.8	0.763033175
		12.2	16.3	10.9	28.8	0.823275862
		19.8	15.1	12.8	12.4	0.914285714
		13.9	23.9	6.1	28.8	0.670103093
		9.4	18.2	4.8	24.5	0.840909091
78.1	70.2	30.4	22.8	23.6	33.6	0.721991701
53	72.4	22.1	13.7	19.2	19.5	0.854111406
37.3	75.3	12.0	18.5	19.0	19.8	0.77027027
51.7	76.8	53.7	20.9	30.9	28.6	0.781021898
影像未收到		11.4	17.3	14.1	9.4	0.811518325
52	77	44.0	24.4	27.7	31.1	0.819875776
52.2	67.4	31.3	13.4	18.3	11.7	0.75170068
63.5	68.9	3.4	7.6	3.7	10.1	1.242738589
		31.6	22.2	18.6	12.3	0.87755102
42.6	37.3	30.0	21.0	20.3	21.5	0.853125
		17.7	12.3	25.6	25.7	0.75
58.1	52.5	27.2	25.2	17.1	19.4	0.896135266
45.5	28.8	13.3	12.4	1.8	21.7	0.848375451
39.8	44.2	30.5	11.3	25.9	15.9	0.886877828
52.7	47.5	33.7	21.3	7.4	15.1	0.606138107

62.7	50.9	15.1	23.9	22.8	29.5	0.797297297
48.7	32.6	21.2	11.6	22.3	11.0	0.880952381
61.7	60.9	21.1	21.4	22.9	14.6	0.76744186
40.5	45.3	11.5	22.8	13.4	8.6	0.658088235
60.2	34.2	5.0	11.6	9.5	12.9	1.045774648
54.3	31.3	7.6	15.7	15.3	6.0	1.106299213
54.3	31.3	43.2	29.9	10.8	14.3	0.714689266
54.9	54.3	45.3	22.2	24.7	26.5	0.773770492
53	32	10.8	20.7	15.7	14.6	0.806026365
55.1	69.8	29.4	22.3	17.9	18.2	0.823529412
63	30	31.8	26.1	20.7	19.4	0.861111111
		#DIV/0!	#DIV/0!	#DIV/0!	#DIV/0!	
62.1	21.9	43.4	22.2	30.2	22.2	0.779605263
45.2	40.4	13.3	13.9	17.6	14.4	0.658450704
47	53	24.0	5.5	3.1	9.1	0.742243437
61.5	42.2	11.6	21.8	18.4	18.9	0.940740741
38.2	52.2	16.1	14.1	22.1	27.2	0.860805861
40.1	35.8	31.0	26.1	21.0	31.4	0.879166667
核醫未做		48.8	25.4	28.0	19.9	0.632318501
45.8	36.5	5.5	18.1	11.3	21.2	0.724590164
		11.2	15.3	13.6	28.9	0.753462604
		24.6	26.3	29.9	19.3	0.845394737
		57.3	44.0	40.6	46.1	0.694736842
		27.0	23.4	19.1	33.7	0.787456446
		64.9	73.3	81.9	90.7	0.908794788
		87.0	95.5	81.7	89.9	0.65034965
		73.7	80.4	71.7	66.2	0.747474747
		28.5	18.9	43.4	28.6	0.994236311
		28.1	26.6	28.0	24.9	0.846153846
		44.8	27.6	27.4	36.4	0.815165877
		21.3	18.2	19.6	44.3	0.954716981
		27.0	55.8	26.9	23.2	0.717741935
		35.0	18.4	18.2	29.5	0.6484375
		14.5	11.0	15.8	16.6	0.785714286
		27.9	33.9	25.8	35.0	0.682719547
		65.0	74.1	63.2	48.8	0.902439024
		31.0	24.1	32.0	32.7	0.798076923
		23.1	49.8	45.6	33.7	1.007843137
		48.3	31.2	40.6	41.0	0.744075829
		21.7	21.9	45.0	48.1	0.989473684
		90.0	45.2	45.2	38.8	0.741935484
		128.7	79.6	37.5	59.5	0.795774648
		50.6	42.9	48.8	46.8	0.75984252

RERLT	RERMAX	VT/Ti_rs	VT/Ti_un	VT/Ti_AT	VT/Ti_pk
0.923147722	1.112273361	0.270886076	0.310638298	2.936842105	3.575714286
0.892307692	1.330327869	0.481280788	0.636567164	0.815540541	2.13125
1.015968064	1.298652291	0.513286713	0.781553398	1.2875	3.510606061
0.944761905	1.264063534	0.39055794	0.735365854	1.479113924	2.969387755
1.024355301	1.360330579	0.5575	0.696638655	0.946456693	2.094318182
0.861538462	1.208596713	0.615107914	0.595744681	1.064356436	3.15
0.893333333	1.263820854	0.352447552	0.509558824	0.834645669	2.119480519
0.983739837	1.197075652	0.67124183	0.933064516	1.515833333	2.126530612
0.967895363	1.181877873	0.505714286	0.830693069	1.193333333	2.740789474
0.802736602	1.124919094	0.813684211	0.762937063	1.264957265	3.2890625
0.805816135	1.143222506	0.625786164	0.828571429	1.221428571	2.327710843
0.919210054	1.086640581	0.445614035	0.869148936	1.383908046	2.454054054
0.786764706	0.988136776	0.62006689	0.805504587	1.075454545	1.724137931
0.863445378	1.182788945	0.360273973	0.720168067	0.986486486	2.487012987
0.907006369	1.050218341	0.560509554	0.741721854	0.853333333	2.356140351
0.824277457	0.936734694	0.402419355	0.6225	0.89047619	1.584
0.888541667	1.196438828	0.576470588	0.567597765	1.161904762	1.873333333
0.896815287	1.160165975	0.594285714	0.726126126	0.895327103	1.93125
0.686719637	1.104584527	0.200675676	0.713461538	1.154320988	1.928421053
0.75	1.133962264	0.275217391	0.789215686	1.02	3.162
0.860036832	0.945883441	0.241438356	0.497163121	0.965771812	2.576923077
0.811394892	1.067057292	0.279863481	0.444444444	1.267692308	2.213043478
0.916452442	1.080392157	0.199209486	0.829605263	1.0984375	2.1
0.998513011	1.742439024	0.252982456	1.03258427	1.75177305	3.22
0.765687053	1.115486726	0.468273092	0.714383562	1.549079755	3.512068966
0.855846774	1.234070221	0.242857143	0.703773585	1.102083333	4.434883721
1.005698006	1.109172763	0.504968944	0.796534653	1.300925926	2.220481928
0.940954774	1.239809783	0.38030303	0.605223881	0.965833333	2.345121951
0.97008547	1.154867257	0.417757009	0.619827586	0.994505495	2.075362319
0.915897992	1.190391459	0.212765957	0.314130435	1.861616162	3.325252525
0.836518047	1.046462955	0.588235294	0.424528302	0.928571429	3.664
0.930207197	1.116010855	0.388571429	0.290780142	0.877862595	1.672881356
0.917293233	1.094392523	1.261304348	0.528242075	1.109465021	2.408411215
0.84389348	1.029301277	0.736363636	0.923076923	1.371568627	2.092783505
0.960194175	1.05632115	0.546363636	0.816216216	1.158823529	1.997402597
0.831081081	0.891861761	0.689565217	1.157142857	0.767213115	1.211111111
0.918773946	1.060752688	0.6425	1.002912621	1.472815534	2.382051282
0.845520082	1.049486461	0.49076087	1.070165746	1.426199262	2.028
0.81980198	1.280732861	0.731067961	0.958921162	0.887610619	2.310526316
0.957894737	0.988414955	0.417037037	0.653913043	1.279329609	1.746226415

0.801564027	1.015151515	0.844117647	0.846067416	1.206593407	2.123880597
0.528350515	1.005956813	0.547826087	0.645273632	0.607518797	2.188
0.809822362	1.090850515	0.598319328	0.875892857	1.022429907	2.256382979
0.780448718	0.925997117	0.623214286	0.247580645	1.103100775	1.672661871
0.897767333	1.154867257	0.338014528	0.438888889	0.859562842	1.693636364
0.909560724	1.074197861	0.483673469	0.924827586	0.934586466	1.837908497
0.879310345	0.98245614	0.684090909	1.073809524	1.129251701	1.720909091
0.836395451	1.18908698	0.508	0.758878505	1.305555556	3.375
0.826397146	1.068635275	0.397368421	0.756521739	0.937209302	1.575221239
0.758683729	1.026560425	0.74375	0.905504587	1.128947368	2.857534247
0.795291709	1.01509434	0.548412698	0.781553398	1.302247191	1.93375
	#DIV/0!	#DIV/0!	#DIV/0!	#DIV/0!	#DIV/0!
0.893141946	1.068273092	0.591743119	0.707189542	1.195348837	1.992405063
0.897664071	1.184349135	0.115358362	0.400507614	0.686144578	1.746728972
0.783852511	1.04515896	0.52761194	0.521223022	0.952537313	2.837962963
0.82559775	1.014181153	0.434883721	1.379569892	2.070422535	4.02
0.856187291	1.150383142	0.231055901	0.576630435	0.637323944	1.435632184
0.832061069	1.092772384	0.631958763	1.208695652	0.92920354	1.927272727
0.773026316	1.061794734	0.4096	0.763106796	1.009090909	2.65
0.81544029	1.083478261	0.250393701	0.858518519	1.485263158	1.691304348
0.820512821	1.085995086	0.288235294	0.879104478	1.15234375	2.883333333
0.834829443	1.073995772	0.823809524	0.738888889	0.904705882	1.507526882
0.812222222	0.961538462	0.473913043	0.698901099	0.95375	1.628571429
0.900166389	1.095730918	0.406451613	0.466060606	0.785714286	1.076923077
0.915644172	1.203452528	0.47628866	0.946774194	0.952542373	1.9025
0.795379538	0.933333333	0.302020202	0.566216216	0.636986301	0.929508197
0.809891808	1.013215859	0.442391304	0.786764706	0.810810811	1.271428571
0.724096386	1.231410702	0.537956204	0.593491124	0.776404494	2.508333333
1.068877551	1.150449714	0.633898305	0.811711712	1.061386139	1.738271605
0.879931389	0.924177396	0.36640625	0.580152672	0.800877193	1.078571429
0.932173913	1.111336032	0.357608696	0.48125	0.628571429	1.41641791
0.763590392	0.947782546	0.20167364	0.512244898	0.620454545	1.155555556
0.90648855	1.088888889	0.3375	0.601298701	0.863779528	1.613414634
0.829007634	1.103726083	0.552	1.038931298	1.140163934	2.179120879
0.8640553	1.067579127	0.404761905	0.818518519	1.135353535	2.164516129
0.844444444	1.029726516	0.513095238	0.931034483	1.155769231	1.3125
0.923636364	0.90273224	0.39483871	0.703076923	0.947474747	1.019354839
0.726126126	1.190789474	0.47483871	0.641489362	1.127142857	1.889393939
0.861678005	1.044573643	0.552380952	0.809009009	1.297297297	1.257575758
0.925675676	0.979301423	0.54248366	1.062135922	1.259701493	1.431147541
0.897893031	0.939831528	0.595652174	1.035064935	1.250724638	1.42972973
0.910313901	1.18207817	0.217204301	0.448809524	0.857142857	1.329032258
0.845679012	0.924855491	0.402777778	0.676344086	0.765060241	0.998701299

RVpred	ERVpred	SVCpred,TLC_RV	SVCpred,SVC_SVC%pred	SVC

SVCpred%	MIPpred	MEPpred	MIPcmH2O	MEPcmH2O	POSTMIP	POSTMEP
	120.45	225.77				
	105.6	197.96				
	119.9	224.74				
	109.45	205.17				
	106.7	200.02				
	104.5	195.9				
	108.35	203.11				
	106.7	200.02				
	101.2	189.72				
	101.75	190.75				
	100.1	187.66				
	107.8	202.08				
	108.35	203.11				
	108.35	203.11				
	107.25	201.05				
	104.5	195.9				
	110.55	207.23				
	106.15	198.99				
	104.5	195.9				
	108.35	203.11				
	110	206.2				
	106.7	200.02				
	106.15	198.99				
	112.2	210.32				
	113.3	212.38				
	113.3	212.38				
	113.3	212.38				
	107.25	201.05				
	105.6	197.96				
	114.4	214.44				
	113.3	212.38				
	114.95	215.47				
	117.15	219.59				
	108.35	203.11				
	107.25	201.05				
	102.3	191.78				
	118.8	222.68				
	113.85	213.41				
	113.3	212.38				
	111.1	208.26				

	109.45	205.17				
	122.1	228.86				
	107.8	202.08				
	115.5	216.5				
	113.85	213.41				
	111.1	208.26				
	111.1	208.26				
	112.2	210.32				
	108.9	204.14				
	108.35	203.11				
	106.15	198.99				
	110	206.2				
	100.1	187.66				
	114.4	214.44				
	118.25	221.65				
	118.25	221.65				
	112.75	211.35				
	106.7	200.02				
	115.5	216.5				
	107.25	201.05				

MIP%	MEP%	postMIP%	postMEP%	IDC_rest	IDC_unl
				0.614785992	0.63800905
				0.46031746	0.348051948
				0.384408602	0.408730159
				0.399656947	0.347457627
				0.409556314	0.444029851
				0.422492401	0.501779359
				0.467320261	0.467353952
				0.311608961	0.402597403
				0.393258427	0.413934426
				0.275362319	0.379310345
				0.381294964	0.443661972
				0.417582418	0.435185185
				0.405698779	0.42578125
				0.41954023	0.381410256
				0.405684755	0.33481153
				0.371257485	0.38585209
				0.345177665	0.481182796
				0.324074074	0.423664122
				0.418079096	0.376811594
				0.519187359	0.435897436
				0.42503639	0.39275766
				0.482701812	0.483870968
				0.43847487	0.390745501
				0.495652174	0.351778656
				0.461111111	0.435820896
				0.495121951	0.409266409
				0.484939759	0.457013575
				0.50867052	0.412307692
				0.419607843	0.415770609
				0.462078652	0.533333333
				0.31835206	0.438016529
				0.389972145	0.621145374
				0.384615385	0.429987608
				0.372180451	0.438202247
				0.331325301	0.395017794
				0.388513514	0.201232033
				0.415224913	0.44017094
				0.35589942	0.367139959
				0.396153846	0.45557656
				0.42721519	0.41218638

				0.227678571	0.361788618
				0.366047745	0.39334638
				0.299748111	0.401433692
				0.144516129	0.449275362
				0.513043478	0.434607646
				0.387658228	0.377604167
				0.385964912	0.411764706
				0.381679389	0.394833948
				0.638655462	0.387205387
				0.338028169	0.402214022
				0.461538462	0.451754386
				#DIV/0!	#DIV/0!
				0.506976744	0.568773234
				0.912772586	0.445701357
				0.372222222	0.526017029
				0.556034483	0.339416058
				0.631372549	0.431924883
				0.301242236	0.4
				0.520833333	0.443965517
				0.585253456	0.400593472
				0.623853211	0.396449704
				0.232686981	0.377622378
				0.381742739	0.421296296
				0.449275362	0.486725664
				0.487437186	0.442857143
				0.426724138	0.490066225
				0.467005076	0.492753623
				0.48409894	0.528125
				0.422939068	0.442231076
				0.450704225	0.453287197
				0.432941176	0.36036036
				0.52297593	0.455813953
				0.418604651	0.442528736
				0.414691943	0.3275
				0.523364486	0.545454545
				0.396226415	0.389261745
				0.478395062	0.485074627
				0.435393258	0.467661692
				0.492957746	0.526066351
				0.448680352	0.420408163
				0.43125	0.458333333
				0.397435897	0.417910448
				0.401486989	0.422727273

IDC_AT	IDC_pk	vrong in ILD case	E_rest (E:I actua	vrong in ILD case	unl (E:I ac
0.286792453	0.466666667	0.626582278	0.626582278	0.570921986	0.570922
0.432748538	0.487804878	1.172413793	1.172413793	1.873134328	1.873134
0.446215139	0.437086093	1.594405594	1.594405594	1.45631068	1.456311
0.398989899	0.497461929	1.502145923	1.502145923	1.87195122	1.871951
0.473880597	0.488888889	1.441666667	1.441666667	1.243697479	1.243697
0.433476395	0.426829268	1.366906475	1.366906475	0.985815603	0.985816
0.488461538	0.496774194	1.13986014	1.13986014	1.139705882	1.139706
0.408163265	0.47804878	2.209150327	2.209150327	1.483870968	1.483871
0.441176471	0.439306358	1.533333333	1.533333333	1.415841584	1.415842
0.383606557	0.412903226	2.631578947	2.631578947	1.636363636	1.636364
0.468619247	0.479768786	1.622641509	1.622641509	1.253968254	1.253968
0.381578947	0.430232558	1.394736842	1.394736842	1.287234043	1.287234
0.421455939	0.395454545	1.464882943	1.464882943	1.348623853	1.348624
0.434017595	0.430167598	1.383561644	1.383561644	1.621848739	1.621849
0.448504983	0.342342342	1.464968153	1.464968153	1.986754967	1.986755
0.470149254	0.497512438	1.693548387	1.693548387	1.591666667	1.591667
0.387096774	0.514705882	1.904411765	1.904411765	1.078212291	1.078212
0.426294821	0.415584416	2.076190476	2.076190476	1.351351351	1.351351
0.312741313	0.475	1.398648649	1.398648649	1.653846154	1.653846
0.468085106	0.462962963	0.926086957	0.926086957	1.294117647	1.294118
0.493377483	0.372759857	1.352739726	1.352739726	1.546099291	1.546099
0.381231672	0.507352941	1.071672355	1.071672355	1.066666667	1.066667
0.383233533	0.464285714	1.280632411	1.280632411	1.559210526	1.559211
0.346437346	0.47008547	1.01754386	1.01754386	1.842696629	1.842697
0.320866142	0.483333333	1.168674699	1.168674699	1.301369863	1.30137
0.398340249	0.457446809	1.019704433	1.019704433	1.443396226	1.443396
0.482142857	0.497005988	1.062111801	1.062111801	1.188118812	1.188119
0.437956204	0.518987342	0.965909091	0.965909091	1.432835821	1.432836
0.431279621	0.475862069	1.38317757	1.38317757	1.405172414	1.405172
0.428571429	0.505102041	1.164133739	1.164133739	0.880434783	0.880435
0.46460177	0.480769231	2.141176471	2.141176471	1.283018868	1.283019
0.461267606	0.453846154	1.564285714	1.564285714	0.609929078	0.609929
0.400329489	0.459227468	1.604347826	1.604347826	1.325648415	1.325648
0.441558442	0.404166667	1.686868687	1.686868687	1.290598291	1.290598
0.414634146	0.420765027	2.009090909	2.009090909	1.531531532	1.531532
0.480314961	0.414746544	1.573913043	1.573913043	3.969387755	3.969388
0.451754386	0.472727273	1.408333333	1.408333333	1.27184466	1.271845
0.311494253	0.409836066	1.809782609	1.809782609	1.723756906	1.723757
0.497797357	0.545977011	1.514563107	1.514563107	1.195020747	1.195021
0.520348837	0.486238532	1.348148148	1.348148148	1.426086957	1.426087

0.38559322	0.465277778	3.392156863	3.392156863	1.764044944	1.764045
0.388888889	0.401606426	1.724637681	1.724637681	1.542288557	1.542289
0.449579832	0.47715736	2.336134454	2.336134454	1.491071429	1.491071
0.416129032	0.471186441	5.919642857	5.919642857	1.225806452	1.225806
0.466836735	0.436507937	0.949152542	0.949152542	1.300925926	1.300926
0.420886076	0.434659091	1.579591837	1.579591837	1.648275862	1.648276
0.4375	0.497737557	1.590909091	1.590909091	1.428571429	1.428571
0.434782609	0.462809917	1.62	1.62	1.53271028	1.53271
0.40952381	0.491304348	0.565789474	0.565789474	1.582608696	1.582609
0.428571429	0.464968153	1.958333333	1.958333333	1.486238532	1.486239
0.357429719	0.404040404	1.166666667	1.166666667	1.223300971	1.223301
#DIV/0!	#DIV/0!	#DIV/0!	#DIV/0!	#DIV/0!	#DIV/0!
0.447916667	0.467455621	0.972477064	0.972477064	0.758169935	0.75817
0.544262295	0.479820628	0.09556314	0.09556314	1.243654822	1.243655
0.578583765	0.509433962	1.686567164	1.686567164	0.901079137	0.901079
0.321266968	0.320512821	0.798449612	0.798449612	1.946236559	1.946237
0.462540717	0.491525424	0.583850932	0.583850932	1.315217391	1.315217
0.418518519	0.44	2.319587629	2.319587629	1.5	1.5
0.464788732	0.483660131	0.92	0.92	1.252427184	1.252427
0.254010695	0.508287293	0.708661417	0.708661417	1.488888889	1.488889
0.42384106	0.5	1.658536585	0.602941176	0.656862745	1.522388
0.3307393	0.417040359	0.302158273	3.30952381	0.606741573	1.648148
0.414507772	0.388888889	0.617449664	1.619565217	0.728	1.373626
0.444816054	0.508379888	0.815789474	1.225806452	0.942857143	1.060606
0.453846154	0.454545455	0.950980392	1.051546392	0.794871795	1.258065
0.459119497	0.516949153	0.744360902	1.343434343	0.961038961	1.040541
0.524822695	0.5625	0.876190476	1.141304348	0.971428571	1.029412
0.438423645	0.434782609	0.938356164	1.065693431	1.119205298	0.893491
0.507537688	0.476470588	0.728395062	1.372881356	0.792857143	1.261261
0.481012658	0.454054054	0.820512821	1.21875	0.829113924	1.206107
0.476780186	0.465277778	0.763485477	1.309782609	0.563380282	1.775
0.487084871	0.529411765	1.101382488	0.907949791	0.837606838	1.193878
0.430508475	0.529032258	0.71641791	1.395833333	0.78974359	1.266234
0.440433213	0.491891892	0.708502024	1.411428571	0.486988848	2.053435
0.471428571	0.484375	1.098039216	0.910714286	1.2	0.833333
0.331210191	0.432432432	0.65625	1.523809524	0.644444444	1.551724
0.495	0.486910995	0.917159763	1.090322581	0.942028986	1.061538
0.421686747	0.461538462	0.771144279	1.296774194	0.886792453	1.12766
0.483660131	0.526595745	0.972222222	1.028571429	1.11	0.900901
0.424050633	0.429577465	0.80952381	1.235294118	0.725352113	1.378641
0.453947368	0.506849315	0.758241758	1.31884058	0.846153846	1.181818
0.373333333	0.504065041	0.659574468	1.516129032	0.717948718	1.392857
0.425641026	0.461077844	0.670807453	1.490740741	0.732283465	1.365591

vrong in ILD case	E_AT (E:I actua	vrong in ILD case	:E_pk (E:I actual	pkBorg/VO2%
2.473684211	2.473684211	1.142857143	1.142857143	4.545454545
1.304054054	1.304054054	1.05	1.05	10
1.241071429	1.241071429	1.287878788	1.287878788	13.04347826
1.506329114	1.506329114	1.010204082	1.010204082	5.882352941
1.11023622	1.11023622	1.045454545	1.045454545	8.333333333
1.297029703	1.297029703	1.342857143	1.342857143	5.617977528
1.047244094	1.047244094	1.012987013	1.012987013	10.97560976
1.441666667	1.441666667	1.091836735	1.091836735	5.128205128
1.266666667	1.266666667	1.263157895	1.263157895	6.18556701
1.606837607	1.606837607	1.421875	1.421875	6.18556701
1.133928571	1.133928571	1.084337349	1.084337349	8.490566038
1.620689655	1.620689655	1.337837838	1.337837838	5.102040816
1.372727273	1.372727273	1.517241379	1.517241379	4.225352113
1.304054054	1.304054054	1.311688312	1.311688312	5.063291139
1.22962963	1.22962963	1.912280702	1.912280702	3.75
1.126984127	1.126984127	1.01	1.01	3.03030303
1.583333333	1.583333333	0.942857143	0.942857143	8.888888889
1.345794393	1.345794393	1.390625	1.390625	4.285714286
2.209876543	2.209876543	1.105263158	1.105263158	3.488372093
1.136363636	1.136363636	1.14	1.14	6.779661017
1.020134228	1.020134228	1.682692308	1.682692308	6.25
1.615384615	1.615384615	0.956521739	0.956521739	9.900990099
1.6171875	1.6171875	1.153846154	1.153846154	7.228915663
1.886524823	1.886524823	1.127272727	1.127272727	3.246753247
2.116564417	2.116564417	1.068965517	1.068965517	8.490566038
1.510416667	1.510416667	1.186046512	1.186046512	4.237288136
1.074074074	1.074074074	1.024096386	1.024096386	10.11235955
1.283333333	1.283333333	0.926829268	0.926829268	4.761904762
1.318681319	1.318681319	1.101449275	1.101449275	6.849315068
1.333333333	1.333333333	0.97979798	0.97979798	6.4
1.152380952	1.152380952	1.08	1.08	5.555555556
1.167938931	1.167938931	1.194915254	1.194915254	2.666666667
1.502057613	1.502057613	1.177570093	1.177570093	8.536585366
1.264705882	1.264705882	1.463917526	1.463917526	4.6875
1.411764706	1.411764706	1.376623377	1.376623377	4.651162791
1.081967213	1.081967213	1.411111111	1.411111111	3.03030303
1.213592233	1.213592233	1.115384615	1.115384615	10.81081081
2.210332103	2.210332103	1.44	1.44	9.803921569
1	1	0.821052632	0.821052632	9.090909091
0.916201117	0.916201117	1.056603774	1.056603774	3.225806452

1.593406593	1.593406593	1.149253731	1.149253731	8.75
1.578947368	1.578947368	1.48	1.48	12.5
1.224299065	1.224299065	1.095744681	1.095744681	11.68831169
1.410852713	1.410852713	1.129496403	1.129496403	6.25
1.142076503	1.142076503	1.290909091	1.290909091	18.36734694
1.37593985	1.37593985	1.300653595	1.300653595	9.090909091
1.285714286	1.285714286	1	1	4.411764706
1.3	1.3	1.160714286	1.160714286	12.19512195
1.441860465	1.441860465	1.03539823	1.03539823	8.333333333
1.333333333	1.333333333	1.150684932	1.150684932	5
1.797752809	1.797752809	1.4625	1.4625	5.797101449
#DIV/0!	#DIV/0!	#DIV/0!	#DIV/0!	
1.244186047	1.244186047	1.139240506	1.139240506	6.849315068
0.843373494	0.843373494	1.08411215	1.08411215	6.060606061
0.728358209	0.728358209	0.962962963	0.962962963	4.62962963
2.112676056	2.112676056	2.12	2.12	3.296703297
1.169014085	1.169014085	1.034482759	1.034482759	12.5
1.389380531	1.389380531	1.272727273	1.272727273	3.921568627
1.151515152	1.151515152	1.067567568	1.067567568	5.263157895
2.936842105	2.936842105	0.97826087	0.97826087	8.064516129
#REF!	1.359375	0.983606557	1.016666667	12.12121212
#REF!	2.023529412	0.709923664	1.408602151	6.666666667
#REF!	1.4125	0.636363636	1.571428571	8.571428571
#REF!	1.240601504	1.034090909	0.967032967	8.695652174
#REF!	1.203389831	0.833333333	1.2	10.60606061
#REF!	1.164383562	1.070175439	0.93442623	11.76470588
0.735632184	0.905405405	1.285714286	0.777777778	9.722222222
0.494186047	1.280898876	0.769230769	1.3	7
0.707964602	0.98019802	0.91011236	1.098765432	10.49382716
0.806060606	1.078947368	0.84	1.19047619	21.95121951
0.830985915	1.097402597	0.881578947	1.134328358	12.16216216
0.858823529	1.053030303	1.125	0.888888889	2.542372881
1.104477612	1.322834646	1.123287671	0.890243902	4.225352113
0.780701754	1.270491803	0.978494624	1.021978022	9.836065574
1.02020202	1.121212121	0.939393939	1.064516129	11.36363636
0.926829268	2.038461538	0.761904762	1.3125	11.68831169
0.911242604	1.03030303	0.939393939	1.064516129	8.536585366
0.949640288	1.371428571	0.846153846	1.181818182	10.84337349
0.755952381	1.081081081	1.1	0.909090909	14.75409836
0.787096774	1.358208955	0.75308642	1.327868852	11.11111111
0.891891892	1.202898551	1.027777778	0.972972973	10.44776119
0.490566038	1.678571429	1.016393443	0.983870968	7.936507937
0.970588235	1.34939759	0.855555556	1.168831169	10.29411765

pkVE/MVV	VT/TE rst	VT/TE unl	VT/TE AT	VT/TE pk	VTpk/FVC
0.76	0.432323232	0.544099379	1.187234043	3.12875	0.595952381
0.73	0.410504202	0.339840637	0.625388601	2.029761905	0.624542125
0.61	0.321929825	0.536666667	1.037410072	2.725882353	0.499353448
0.64	0.26	0.392833876	0.981932773	2.939393939	0.684705882
0.53	0.386705202	0.560135135	0.85248227	2.00326087	0.590705128
0.73	0.45	0.604316547	0.820610687	2.345744681	0.619382022
0.54	0.309202454	0.447096774	0.796992481	2.092307692	0.477192982
0.65	0.303846154	0.628804348	1.051445087	1.947663551	0.681045752
0.62	0.329813665	0.586713287	0.942105263	2.169791667	0.567574932
0.75	0.3092	0.466239316	0.787234043	2.313186813	0.635951662
0.76	0.385658915	0.660759494	1.077165354	2.146666667	0.769721116
0.63	0.319496855	0.675206612	0.853900709	1.834343434	0.613513514
0.44	0.423287671	0.597278912	0.783443709	1.136363636	0.506756757
0.5	0.26039604	0.444041451	0.756476684	1.896039604	0.436218679
0.46	0.382608696	0.373333333	0.693975904	1.232110092	0.701305483
0.47	0.237619048	0.391099476	0.790140845	1.568316832	0.472835821
0.58	0.302702703	0.52642487	0.733834586	1.986868687	0.622468354
0.46	0.286238532	0.537333333	0.665277778	1.388764045	0.38625
0.47	0.143478261	0.431395349	0.522346369	1.744761905	0.513165266
0.78	0.297183099	0.609848485	0.8976	2.773684211	0.43553719
0.45	0.178481013	0.321559633	0.946710526	1.531428571	0.594235033
0.78	0.261146497	0.416666667	0.784761905	2.313636364	0.512416107
0.51	0.155555556	0.532067511	0.679227053	1.82	0.531386861
0.69	0.24862069	0.560365854	0.928571429	2.856451613	0.457622739
0.7	0.400687285	0.548947368	0.731884058	3.285483871	0.427044025
1.02	0.238164251	0.487581699	0.729655172	3.739215686	0.531197772
0.45	0.475438596	0.670416667	1.211206897	2.168235294	0.395493562
0.63	0.39372549	0.422395833	0.752597403	2.530263158	0.495618557
0.55	0.302027027	0.441104294	0.754166667	1.884210526	0.456050955
0.63	0.182767624	0.356790123	1.396212121	3.393814433	0.669105691
0.86	0.274725275	0.330882353	0.805785124	3.392592593	0.462626263
0.34	0.248401826	0.476744186	0.751633987	1.4	0.5140625
0.39	0.786178862	0.398478261	0.738630137	2.045238095	0.495576923
0.41	0.436526946	0.715231788	1.084496124	1.429577465	0.550135501
0.65	0.271945701	0.532941176	0.820833333	1.450943396	0.555234657
0.49	0.438121547	0.29151671	0.709090909	0.858267717	0.516587678
0.54	0.456213018	0.788549618	1.2136	2.135632184	0.46566416
0.41	0.271171171	0.620833333	0.64524207	1.408333333	0.357042254
0.66	0.482692308	0.802430556	0.887610619	2.814102564	0.554292929
0.39	0.309340659	0.458536585	1.396341463	1.652678571	0.509917355

0.64	0.248843931	0.479617834	0.757241379	1.848051948	0.532958801
0.41	0.317647059	0.418387097	0.384761905	1.478378378	0.54563591
0.58	0.256115108	0.58742515	0.835114504	2.059223301	0.545244216
0.34	0.105279035	0.201973684	0.781868132	1.48089172	0.506535948
0.29	0.356122449	0.337366548	0.752631579	1.311971831	0.371856287
0.42	0.30620155	0.561087866	0.679234973	1.413065327	0.831952663
0.51	0.43	0.751666667	0.878306878	1.720909091	0.547109827
0.76	0.313580247	0.495121951	1.004273504	2.907692308	0.489637306
0.48	0.702325581	0.478021978	0.65	1.521367521	0.532934132
0.87	0.379787234	0.609259259	0.846710526	2.483333333	0.668589744
0.48	0.470068027	0.638888889	0.724375	1.322222222	0.510561056
	#DIV/0!	#DIV/0!	#DIV/0!	#DIV/0!	
0.61	0.608490566	0.932758621	0.960747664	1.748888889	0.568231047
0.39	1.207142857	0.322040816	0.813571429	1.611206897	0.485454545
0.56	0.312831858	0.578443114	1.307786885	2.947115385	0.619191919
0.82	0.544660194	0.708839779	0.98	1.896226415	0.592920354
0.38	0.395744681	0.438429752	0.545180723	1.387777778	0.298090692
0.73	0.272444444	0.805797101	0.668789809	1.514285714	0.493023256
0.67	0.445217391	0.609302326	0.876315789	2.482278481	0.546239554
0.5	0.353333333	0.576616915	0.505734767	1.728888889	0.53105802
0.646227833	0.47804878	0.57745098	0.847701149	2.836065574	0.471389646
0.649905935	0.248920863	0.448314607	0.447093023	1.070229008	0.994326241
0.703703704	0.29261745	0.5088	0.675221239	1.036363636	0.58089172
0.396110911	0.331578947	0.439428571	0.633333333	1.113636364	0.547486034
0.983544543	0.452941176	0.752564103	0.791549296	1.585416667	0.543571429
0.458570525	0.22481203	0.544155844	0.547058824	0.994736842	0.44296875
0.699300699	0.387619048	0.764285714	0.895522388	1.634693878	0.48253012
0.959409594	0.504794521	0.664238411	0.606140351	1.929487179	0.76010101
0.464425042	0.461728395	0.643571429	1.082828283	1.582022472	0.391111111
0.372463396	0.300641026	0.481012658	0.742276423	0.906	0.344486692
0.656491055	0.273029046	0.271126761	0.572781065	1.248684211	0.502116402
0.922693267	0.222119816	0.429059829	0.589208633	1.3	1.16635514
0.715889949	0.241791045	0.474871795	0.65297619	1.812328767	0.520866142
0.857678906	0.391093117	0.505947955	0.897419355	2.132258065	0.665436242
0.707944713	0.444444444	0.982222222	1.012612613	2.033333333	0.627102804
0.870455709	0.33671875	0.6	0.566981132	1	0.591549296
0.669194736	0.362130178	0.662318841	0.919607843	0.957575758	0.764516129
0.577200577	0.366169154	0.568867925	0.821875	1.598717949	0.546929825
1.458523245	0.537037037	0.898	1.2	1.383333333	1.004032258
0.69509675	0.439153439	0.770422535	0.927472527	1.077777778	0.452331606
0.660218025	0.451648352	0.875824176	1.039759036	1.469444444	0.714864865
0.522875817	0.143262411	0.322222222	0.510638298	1.350819672	0.379723502
0.632768362	0.270186335	0.495275591	0.566964286	0.854444444	0.668695652

VTpk/IC	VTrst/FVC	VTrst/IC	VTunl/FVC	VTunl/IC	VTat/FVC
1.021632653	0.203809524	0.349387755	0.208571429	0.35755102	0.531428571
0.848258706	0.357875458	0.486069652	0.312454212	0.424378109	0.442124542
0.721806854	0.158189655	0.229375	0.173491379	0.2515625	0.310775862
0.771883289	0.214117647	0.24137931	0.283764706	0.319893899	0.549882353
0.758436214	0.214423077	0.275308642	0.265705128	0.341152263	0.38525641
0.752559727	0.240168539	0.290816327	0.235955056	0.285714286	0.301966292
0.663414634	0.147368421	0.204878049	0.202631579	0.281707317	0.30994152
0.801538462	0.335620915	0.39348659	0.378104575	0.443295019	0.594444444
0.623652695	0.144686649	0.159459459	0.228610354	0.251951952	0.292643052
0.782527881	0.233534743	0.287360595	0.329607251	0.405576208	0.447129909
0.7546875	0.396414343	0.388671875	0.415936255	0.4078125	0.54501992
0.687878788	0.171621622	0.192424242	0.276013514	0.309469697	0.406756757
0.688073394	0.626351351	0.850458716	0.296621622	0.402752294	0.399662162
0.550287356	0.179726651	0.226724138	0.195216401	0.246264368	0.332574032
0.836760125	0.229765013	0.274143302	0.292428198	0.348909657	0.30078329
0.784158416	0.148955224	0.247029703	0.222985075	0.36980198	0.334925373
0.768359375	0.248101266	0.30625	0.321518987	0.396875	0.308860759
0.438297872	0.195	0.221276596	0.251875	0.285815603	0.299375
0.565432099	0.083193277	0.091666667	0.207843137	0.229012346	0.261904762
0.528762542	0.174380165	0.211705686	0.221763085	0.269230769	0.309090909
0.960573477	0.15631929	0.253597122	0.155432373	0.252158273	0.319068736
0.625819672	0.275167785	0.336065574	0.268456376	0.327868852	0.553020134
0.697763578	0.122627737	0.161022364	0.306812652	0.402875399	0.342092457
0.558675079	0.18630491	0.227444795	0.2374677	0.289905363	0.638242894
0.608059701	0.244444444	0.348059701	0.218658281	0.311343284	0.529350105
0.895305164	0.137325905	0.231455399	0.207799443	0.350234742	0.294707521
0.5759375	0.174463519	0.254858934	0.34527897	0.504388715	0.301502146
0.643143813	0.258762887	0.335785953	0.209020619	0.271237458	0.29871134
0.734358974	0.142356688	0.229230769	0.228980892	0.368717949	0.288216561
0.924719101	0.142276423	0.196629213	0.117479675	0.162359551	0.374593496
0.534110787	0.126262626	0.145772595	0.113636364	0.131195335	0.246212121
0.85826087	0.141666667	0.236521739	0.213541667	0.356521739	0.299479167
0.67109375	0.557884615	0.757441253	0.3525	0.478590078	0.518461538
0.732851986	0.197560976	0.262230216	0.292682927	0.388489209	0.379132791
0.712037037	0.216967509	0.278240741	0.327075812	0.419444444	0.426714801
0.598901099	0.375829384	0.433333333	0.537440758	0.619672131	0.443601896
0.540116279	0.193233083	0.224127907	0.258897243	0.300290698	0.380200501
0.555109489	0.211971831	0.329562044	0.454694836	0.706934307	0.907276995
0.675384615	0.190151515	0.231692308	0.583585859	0.711076923	0.253282828
0.566055046	0.155096419	0.172171254	0.207162534	0.229969419	0.630853994

0.704455446	0.32247191	0.428358209	0.282022472	0.374626866	0.411235955
0.719736842	0.188528678	0.248684211	0.323441397	0.426644737	0.201496259
0.815769231	0.183033419	0.273846154	0.25218509	0.377307692	0.281233933
0.582706767	0.152069717	0.174937343	0.066884532	0.076942356	0.310021786
0.441469194	0.278642715	0.331591449	0.189221557	0.225178147	0.313972056
0.983216783	0.350591716	0.414335664	0.396745562	0.468881119	0.367751479
0.688363636	0.173988439	0.218909091	0.260693642	0.328	0.479768786
0.605769231	0.131606218	0.162820513	0.210362694	0.26025641	0.304404145
0.637992832	0.361676647	0.434532374	0.260479042	0.31294964	0.361976048
0.737102473	0.228846154	0.25229682	0.316346154	0.348763251	0.4125
0.626315789	0.228052805	0.279757085	0.265676568	0.325910931	0.382508251
0.790954774	0.232851986	0.324120603	0.390613718	0.543718593	0.371119134
0.692222222	0.175584416	0.25037037	0.204935065	0.292222222	0.295844156
0.754926108	0.142828283	0.174137931	0.585454545	0.713793103	0.644646465
0.646302251	0.330973451	0.361935484	0.378466077	0.413870968	0.433628319
0.37172619	0.177565632	0.221428571	0.253221957	0.31577381	0.215990453
0.617475728	0.237596899	0.297572816	0.431007752	0.539805825	0.406976744
0.697864769	0.142618384	0.182857143	0.218941504	0.280714286	0.278272981
0.72037037	0.434129693	0.588888889	0.39556314	0.536574074	0.481569966
0.686507937	0.267029973	0.388888889	0.320980926	0.467460317	0.401907357
0.916339869	0.490780142	0.452287582	0.565957447	0.521568627	0.545390071
0.651428571	0.277707006	0.311428571	0.405095541	0.454285714	0.485987261
0.91588785	0.351955307	0.588785047	0.429608939	0.718691589	0.583798883
0.70462963	0.33	0.427777778	0.419285714	0.543518519	0.401428571
0.616304348	0.23359375	0.325	0.32734375	0.455434783	0.36328125
0.696521739	0.245180723	0.353913043	0.322289157	0.465217391	0.361445783
1.106617647	0.372222222	0.541911765	0.506565657	0.7375	0.348989899
0.848192771	0.207777778	0.45060241	0.250277778	0.542771084	0.297777778
0.526744186	0.178326996	0.272674419	0.288973384	0.441860465	0.347148289
0.747244094	0.348148148	0.518110236	0.407407407	0.606299213	0.512169312
1.485714286	0.45046729	0.573809524	0.469158879	0.597619048	0.765420561
0.792215569	0.191338583	0.291017964	0.364566929	0.554491018	0.431889764
0.909633028	0.324161074	0.443119266	0.456711409	0.624311927	0.466778523
0.737362637	0.317757009	0.373626374	0.413084112	0.485714286	0.525233645
0.730434783	0.303521127	0.374782609	0.38028169	0.469565217	0.423239437
0.783471074	0.493548387	0.505785124	0.737096774	0.755371901	0.756451613
0.708522727	0.322807018	0.418181818	0.264473684	0.342613636	0.346052632
1.174528302	0.467741935	0.547169811	0.724193548	0.847169811	0.774193548
0.549056604	0.430051813	0.522012579	0.566839378	0.688050314	0.437305699
0.745070423	0.277702703	0.28943662	0.538513514	0.561267606	0.583108108
0.496385542	0.093087558	0.121686747	0.173732719	0.227108434	0.331797235
1.2015625	0.37826087	0.6796875	0.546956522	0.9828125	0.552173913

VTat/IC	HR_rest%	HR_unl%	HR_AT%	O2P_rest%	O2P_unl%	O2p_AT%	CO2rest/k
0.911020408	52.0%	49.7%	77.7%	0.27462	0.34327	1.071	3.51389
0.600497512	50.7%	53.3%	61.8%	0.20059	0.43607	0.60177	2.92105
0.450625	61.2%	57.9%	83.1%	0.1589	0.27808	0.45023	2.97468
0.619893899	52.8%	59.1%	67.3%	0.20035	0.37209	0.70124	2.29762
0.494650206	50.0%	50.0%	68.2%	0.31156	0.46735	0.6048	3.89286
0.365646259	65.3%	69.3%	73.3%	0.29535	0.30379	0.59915	4.69014
0.430894309	46.5%	49.0%	59.9%	0.34235	0.52253	0.7928	3.65574
0.696934866	51.9%	57.8%	70.8%	0.32073	0.56509	0.77891	3.74074
0.322522523	52.1%	60.4%	68.8%	0.33934	0.61448	0.77957	3.22667
0.550185874	53.8%	56.6%	72.4%	0.37325	0.57352	0.7647	3.5
0.534375	50.7%	90.1%	88.0%	0.41383	0.44271	0.81804	3.89333
0.456060606	67.3%	70.5%	80.8%	0.14527	0.50419	0.75201	2.94985
0.54266055	51.6%	58.6%	68.2%	0.35005	0.6223	0.79344	4.7125
0.41954023	54.1%	56.1%	67.5%	0.22593	0.45966	0.70117	2.98201
0.358878505	55.5%	62.6%	75.5%	0.33392	0.55051	0.60466	5.20968
0.555445545	51.3%	50.0%	70.0%	0.35357	0.57582	0.82837	2.94545
0.38125	42.9%	44.7%	61.5%	0.49105	0.69079	0.80731	4.95238
0.339716312	56.9%	61.4%	62.1%	0.39996	0.55994	0.7377	4.39063
0.288580247	42.0%	48.7%	70.0%	0.18481	0.73926	0.77622	1.30769
0.375250836	47.1%	52.9%	66.9%	0.37576	0.769	0.98747	3.4375
0.517625899	46.9%	48.1%	67.5%	0.19893	0.40496	0.71756	2.03797
0.675409836	40.3%	46.8%	68.2%	0.37467	0.5772	0.98225	3.67308
0.449201278	45.1%	54.9%	61.4%	0.083	0.6557	0.6889	0.95522
0.779179811	50.6%	57.3%	75.0%	0.4312	0.88704	1.34288	2.6
0.753731343	44.0%	44.0%	67.5%	0.37372	0.57615	0.87201	4.28986
0.496713615	32.5%	35.5%	47.6%	0.38208	0.83209	1.06983	2.9
0.440438871	50.6%	57.8%	74.7%	0.37413	0.54872	0.70669	5.64912
0.387625418	53.5%	59.4%	72.9%	0.39803	0.4511	0.61916	4.67213
0.464102564	39.5%	48.0%	58.6%	0.37641	0.49916	0.68736	2.85333
0.517696629	49.4%	53.0%	74.4%	0.17189	0.26157	1.09858	2.1831
0.28425656	41.0%	47.0%	62.0%	0.35271	0.39023	0.6829	3.34177
0.5	33.1%	41.4%	65.1%	0.45575	0.48155	0.71372	3.94643
0.703916449	49.7%	49.1%	60.7%	0.37122	0.39774	0.66953	7.30488
0.50323741	37.6%	43.9%	57.3%	0.43785	0.64168	0.91345	3.7625
0.547222222	41.9%	48.4%	61.9%	0.39135	0.69485	0.85459	3.5
0.51147541	52.7%	54.8%	63.7%	0.32227	0.51564	0.8594	3.10909
0.440988372	51.7%	56.3%	68.2%	0.3221	0.53216	0.76323	4.58025
1.410583942	49.7%	52.7%	64.1%	0.26243	0.4851	0.72367	3.91667
0.308615385	57.8%	65.7%	72.9%	0.3475	0.55902	0.62701	4.9
0.70030581	52.5%	51.2%	64.8%	0.36495	0.56329	1.00757	3.59091

0.546268657	33.3%	41.5%	51.6%	0.56212	0.75485	1.00379	3.83117
0.265789474	50.0%	52.7%	57.7%	0.20815	0.36427	0.48136	2.97701
0.420769231	56.4%	57.1%	62.8%	0.26315	0.55726	0.75849	3.08
0.356641604	51.8%	54.1%	67.1%	0.20259	0.18299	0.71235	1.84536
0.373634204	45.5%	47.9%	57.5%	0.26794	0.4345	0.6445	3.57831
0.434615385	44.4%	50.0%	56.8%	0.29184	0.65872	0.70041	3.64935
0.603636364	50.0%	54.9%	56.2%	0.35432	0.59589	0.66837	3.61429
0.376602564	36.0%	45.7%	61.0%	0.37779	0.51583	0.82824	2.77647
0.434892086	41.8%	42.4%	44.9%	0.60527	0.69606	0.89277	5.48718
0.454770318	59.2%	66.2%	71.3%	0.35028	0.52542	0.81732	4.41096
0.469230769	54.9%	57.5%	68.0%	0.3426	0.48602	0.74895	4.18919
				#DIV/0!	#DIV/0!	#DIV/0!	
0.516582915	62.7%	70.4%	78.2%	0.35386	0.63487	0.58283	4.30909
0.421851852	48.8%	54.2%	79.2%	0.29201	0.39213	0.56733	3.10631
0.785960591	37.1%	40.0%	63.4%	0.43699	0.62818	0.96958	3.8875
0.474193548	45.1%	50.9%	61.7%	0.37153	0.6775	0.96162	4.94805
0.269345238	52.1%	55.8%	61.2%	0.24276	0.37172	0.44759	3.30986
0.509708738	48.7%	59.7%	61.7%	0.27112	0.63544	0.5846	3.10294
0.356785714	46.5%	51.2%	54.7%	0.3749	0.61788	0.68037	3.03371
0.653240741	54.2%	57.4%	65.8%	0.30083	0.70195	0.67688	3.2029
0.58531746	57.9%	60.8%	71.2%	0.25222	0.40636	0.60253	3.67568
0.502614379	49.5%	55.9%	60.4%	0.48111	0.7155	0.72783	3.86466
0.545	55.8%	60.9%	77.3%	0.5479	0.89193	0.9429	4.19048
0.976635514	41.4%	52.8%	63.4%	0.61483	0.69503	0.84205	4.34615
0.52037037	56.4%	73.9%	79.0%	0.43963	0.70342	0.66573	4.57377
0.505434783	46.0%	53.6%	56.3%	0.39562	0.68652	0.67488	3.75
0.52173913	69.2%	76.3%	80.2%	0.34326	0.57618	0.63748	3.41538
0.508088235	48.1%	51.1%	55.4%	0.49664	0.76228	1.03947	5.84746
0.645783133	49.1%	57.4%	67.8%	0.48809	0.57422	0.76563	5.31034
0.530813953	50.4%	55.6%	62.8%	0.24207	0.40346	0.54691	2.81967
0.762204724	44.8%	44.8%	55.5%	0.439	0.67731	0.77765	5.27083
0.975	45.5%	59.1%	62.4%	0.45467	0.85737	1.06522	2.96667
0.656886228	51.2%	60.6%	67.2%	0.4118	0.61147	0.6489	2.47761
0.638073394	60.3%	66.1%	68.0%	0.30341	0.49304	0.58786	4.05263
0.617582418	50.1%	57.6%	70.7%	0.52918	0.81782	0.92606	3.95082
0.522608696	64.0%	68.0%	70.7%	0.41199	0.65919	0.81026	4.70909
0.775206612	60.4%	70.3%	86.2%	0.45935	0.7836	0.85115	3.95238
0.448295455	52.6%	54.0%	59.6%	0.33506	0.59129	1.27126	4.28333
0.905660377	68.2%	97.0%	87.6%	0.36222	0.35339	0.59192	4.07792
0.53081761	42.7%	50.5%	66.1%	0.70757	1.04661	1.17928	4.94737
0.607746479	59.5%	68.2%	74.8%	0.37762	0.63307	0.66639	3.50847
0.43373494	54.7%	59.1%	72.0%	0.15072	0.30145	0.55983	1.85246
0.9921875	56.8%	67.2%	69.3%	0.43946	0.6521	0.69463	3.38596

VCO2unl/k	VCO2AT/k	VCO2pk/k	T+EELV_	T+EELV_u	T+EELV_A	T+EELV_	EELV_rst)	EELV_unl
4.86111	27.8611	44.3056	3.776	4.276	5.002	5.363	0.64658	0.73219
4.05263	7.63158	21.3553	#######	#######	#######	#######	#######	#######
5.10127	12.8861	30.4937	#######	#######	#######	#######	#######	#######
4.66667	11.8095	22.7381	#######	#######	#######	#######	#######	#######
6.44643	12.7679	29.3929	#######	#######	#######	#######	#######	#######
5.70423	9.46479	26.9296	#######	#######	#######	#######	#######	#######
5.81967	12.082	29.6066	#######	2.513	3.23	3.962	#######	0.51496
7.2963	13.4444	23.2469	#######	4.077	4.199	4.564	#######	0.67056
6.84	10.8533	24	#######	4.379	4.714	5.893	#######	0.63741
5.80882	10.3529	25.5588	#######	3.881	4.49	5.215	#######	0.60546
6.30667	11.4533	23.84	#######	3.484	4.048	4.482	#######	0.71687
8.67257	15.1032	28.6726	2.358	1.867	2.644	4.046	0.50385	0.39893
6.975	10.7	17.7	3.994	3.098	3.023	4.04	0.82521	0.64008
5.80977	10.5656	24.2031	4.599	5.767	4.57	5.775	0.67533	0.84684
7.33871	11.4839	23.2742	5.47	4.68	5.362	6.346	0.78592	0.67241
5.81818	12.9636	25.0364	2.899	3.007	2.782	3.134	0.52709	0.54673
7.69841	13.5397	33.0635	4.144	4.636	4.466	5.427	0.69882	0.78179
8.25	11	21.8438	3.944	3.076	4.618	4.076	0.73173	0.57069
5.83077	9.30769	23.7231	2.777	3.702	4.405	5.112	0.47228	0.62959
8.23438	13.9219	37.5625	3.163	3.165	3.532	4.511	0.54913	0.54948
4.03797	11.8228	25.8861	2.805	2.451	2.919	4.78	0.41866	0.36582
5.40385	15.8846	31.5192	2.25	2.81	3.698	4.087	0.4386	0.54776
7.89552	10.6418	24.6716	5.284	5.081	5.286	6.214	0.73593	0.70766
7.81333	17.9067	47.6267	2.811	2.409	4.46	3.641	0.51202	0.4388
6.13043	13.971	36.5362	3.716	2.843	5.075	4.647	0.5588	0.42752
7.78333	14.15	47.45	#######	3.716	3.878	5.157	#######	0.63521
10.4211	18.5789	34.5789	#######	5.319	3.115	5.053	#######	0.73773
6.39344	12.2787	29.918	2.504	3.591	2.899	4.373	0.40715	0.5839
5.05333	9.08	20.88	3.837	4.279	4.135	#######	0.70275	0.7837
3.38028	23.7746	47.1127	4.24	2.878	4.813	6.092	0.58971	0.40028
3.55696	9.97468	31.6456	3.99	3.8	4.395	5.502	0.66611	0.63439
5.80357	15.2321	29.375	5.164	4.78	5.28	5.934	0.79202	0.73313
5.30488	11.9024	28.561	6.021	3.993	4.326	5.907	0.7996	0.53028
6.6625	11.4875	17.125	4.409	4.14	5.249	5.28	0.71925	0.67537
7.08974	12.6795	22.6026	3.731	4.456	4.382	3.918	0.72447	0.86524
6.32727	11.1818	14.5455	3.723	3.914	3.776	4.03	0.75364	0.79231
8.65432	14.8025	24.358	#######	3.773	4.707	5.048	#######	0.6145
9.06667	13.6833	18.7333	3.673	4.457	5.035	3.811	0.59051	0.71656
10.5125	10.35	27.0875	1.903	3.441	2.573	4.515	0.36317	0.65668
6.16667	19.303	28.4394	2.823	3.412	4.82	5.111	0.46584	0.56304

5.66234	10.6494	20.8831	2.841	3.753	3.998	4.503	0.59188	0.78188
4.68966	4.71264	15.5287	3.336	3.027	4.488	4.778	0.58118	0.52735
6.72	10.3333	22.5733	3.932	4.151	3.314	5.581	0.62215	0.6568
1.63918	10.0412	19.866	3.948	3.517	4.763	5.635	0.57974	0.51645
4.43373	9.20482	15.7229	5.266	#######	5.173	6.063	0.6839	#######
7.41558	9.14286	20.8701	2.725	3.101	3.143	5.032	0.51222	0.58289
7.68571	9.47143	19.2	3.582	3.862	4.41	4.873	0.63623	0.68597
5.02353	11.2471	25.8941	#######	3.652	4.235	5.1	#######	0.58526
6.11538	8.91026	17.1667	4.908	4.2	5.039	5.35	0.81393	0.69652
7.68493	11.3699	31.7671	3.944	4.217	4.367	4.896	0.72634	0.77661
6.28378	10.5	18.1757	3.531	3.265	3.599	4.147	0.69508	0.64272
			#######	#######	#######	#######	#######	#######
8.38182	10.1818	19.3455	4.705	3.792	4.538	4.764	0.8539	0.6882
5.66445	13.4053	26.1462	4.746	4.909	5.039	6.299	0.73015	0.75523
6.425	15.4125	36.1625	4.137	4.908	5.101	5.425	0.5987	0.71027
9.48052	15.2468	28.7922	3.952	3.613	2.13	2.84	0.72781	0.66538
5.67606	7.21127	16.9155	5.534	5.811	5.655	6.159	0.79626	0.83612
8.70588	8.01471	14.8971	3.613	4.802	4.33	4.772	0.63721	0.84691
5.67416	7.92135	22.2022	3.242	3.386	3.849	5.151	0.55136	0.57585
8.17391	9.7971	18.058	4.892	4.119	4.601	4.276	0.88623	0.7462
6.95946	11.6757	23.8919	4.15	3.538	3.665	3.88	0.71924	0.61317
6.49624	6.99248	15.2782	2.722	2.868	3.029	3.432	0.74986	0.79008
8.03175	11.6032	19.8413	2.276	1.446	1.753	1.452	0.83066	0.52774
6.21154	10.4038	16.2885	4.24	4.999	5.455	5.3	1.01435	1.19593
9.14754	9.78689	16	2.172	2.547	2.592	2.641	0.75156	0.88131
8.79032	9.71774	15.2419	#######	2.379	2.445	2.717	#######	0.75285
6.75385	8.06154	14.1538	2.467	2.375	2.7	2.931	0.76854	0.73988
8.79661	10.1864	30.0339	2.457	2.633	2.561	3.375	0.58921	0.63141
8.27586	14.4483	24.2586	3.988	3.841	3.912	4.848	0.68288	0.65771
4.95082	8.40984	10.5902	#######	#######	#######	#######	#######	#######
6.6875	11.1667	22.875	3.718	3.39	3.808	3.569	0.97076	0.88512
6.93333	10.0667	22.0833	3.092	3.112	4.169	#######	0.76915	0.77413
5.68657	7.08955	13.8955	#######	#######	3.407	3.623	#######	#######
7.07018	9.52632	19.2281	#######	#######	#######	4.453	#######	#######
8.14754	12.2951	20.459	3.53	3.274	3.484	3.522	0.93634	0.86844
8.63636	9.67273	15.7455	1.921	1.93	#######	2.4	0.68607	0.68929
8.38095	12.0952	13.1111	2.112	2.064	1.918	1.928	0.64985	0.63508
6.2	13.4333	24.1333	3.126	3.133	3.039	3.077	0.73901	0.74066
6.97403	9.87013	14	2.58	2.858	2.62	2.905	0.70879	0.78516
9.70175	12.0175	13.2807	3.31	3.214	3.324	3.193	0.85974	0.83481
8.01695	9.38983	13.2373	2.571	2.777	2.973	3.138	0.73879	0.79799
4.31148	9.98361	20.3279	2.882	3.297	3.38	3.714	0.67023	0.76674
6.7193	7.21053	11.2281	1.925	1.939	2.185	2.089	0.8443	0.85044

EELV_AT	EELV_pk)	test1	test2	test3	test4	test5	test6	test7
0.85651	0.91832							
#######	#######							
#######	#######							
#######	#######							
#######	#######							
#######	#######							
0.66189	0.81189							
0.69063	0.75066							
0.68617	0.85779							
0.70047	0.81357							
0.83292	0.92222							
0.56496	0.86453							
0.62459	0.83471							
0.67107	0.84802							
0.7704	0.91178							
0.50582	0.56982							
0.75312	0.91518							
0.85677	0.75622							
0.74915	0.86939							
0.61319	0.78316							
0.43567	0.71343							
0.72086	0.79669							
0.73621	0.86546							
0.81239	0.66321							
0.76316	0.6988							
0.66291	0.88154							
0.43204	0.70083							
0.47138	0.71106							
0.75733	#######							
0.6694	0.84729							
0.73372	0.91853							
0.80982	0.91012							
0.5745	0.78446							
0.85628	0.86134							
0.85087	0.76078							
0.76437	0.81579							
0.76661	0.82215							
0.80949	0.6127							
0.49103	0.86164							
0.79538	0.8434							

0.83292	0.93813							
0.78188	0.8324							
0.52437	0.88307							
0.69941	0.82746							
0.67182	0.7874							
0.59079	0.94586							
0.7833	0.86554							
0.67869	0.81731							
0.83566	0.88723							
0.80424	0.90166							
0.70846	0.81634							
#######	#######							
0.82359	0.86461							
0.77523	0.96908							
0.73821	0.78509							
0.39227	0.52302							
0.81367	0.88619							
0.76367	0.84162							
0.65459	0.87602							
0.83351	0.77464							
0.63518	0.67244	5	6	4	2	3	6	7
0.83444	0.94545	5	4	6	4	2	6	5
0.63978	0.52993	1	2	2	2	2	2	5
1.30502	1.26794	1	2	1	7	1	7	2
0.89689	0.91384	1	5	7	7	7	7	7
0.77373	0.85981	5	5	6	4	2	7	7
0.84112	0.91308	2	4	4	4	4	4	4
0.61415	0.80935	1	5	7	7	1	7	7
0.66986	0.83014	7	7	7	7	1	7	7
#######	#######	1	7	7	2	1	5	7
0.99426	0.93185	2	7	4	7	1	2	5
1.03706	#######	7	7	7	7	1	7	7
0.61609	0.65515	1	7	7	7	1	7	5
#######	0.80524	7	7	7	7	1	7	7
0.92414	0.93422	1	6	7	4	6	7	7
#######	0.85714	6	7	7	7	3	7	7
0.59015	0.59323	6	5	7	7	3	7	7
0.71844	0.72742	2	3	7	7	5	7	7
0.71978	0.79808	1	4	3	2	5	3	3
0.86338	0.82935	3	3	4	3	3	4	5
0.85431	0.90172	2	7	5	5	3	5	7
0.78605	0.86372	5	6	5	4	1	5	7
0.95833	0.91623							

test8	test9	test10	test11	test12	test13	test14	test15	test_sum

7	7	7	3	7	6	7	6	83
6	6	6	6	6	4	7	7	80
2	7	2	2	2	2	2	4	39
6	7	7	7	7	7	2	7	71
7	7	7	7	7	7	7	7	97
7	7	7	7	7	7	7	7	92
4	4	4	1	4	2	2	4	51
7	7	7	7	7	7	7	7	91
7	7	7	7	7	7	7	7	99
5	7	7	2	7	7	5	7	77
5	6	6	5	6	4	7	4	71
7	7	7	7	7	7	7	7	99
7	7	7	7	7	7	7	7	91
7	7	7	7	7	7	7	7	99
7	7	7	7	7	7	7	7	94
7	5	7	6	7	1	7	7	91
7	7	7	7	7	7	7	7	98
7	7	5	7	7	7	7	7	92
3	7	7	5	7	2	7	7	66
5	7	7	2	3	2	7	7	65
5	7	7	2	7	7	5	7	81
5	7	5	7	5	5	7	5	79

test_sum%	um_dysp4	um_chest3	um_psych	_sum_dys	_sum_che	_sum_psy	um_dysp-ch	Entry date (Y/M/D)

76	16	20	41	50	94	81	69	2/13/2020
72	19	15	39	63	67	76	64	5/22/2020
27	7	14	14	13	61	17	33	6/3/2020
62	22	11	31	75	44	57	62	10/14/2020
91	22	19	49	75	89	100	81	6/12/2020
86	23	19	43	79	89	86	83	8/7/2020
40	9	12	26	21	50	45	33	8/5/2020
84	22	19	43	75	89	86	81	9/30/2020
93	28	21	43	100	100	86	100	10/7/2020
69	12	21	37	33	100	71	62	10/14/2020
62	18	18	31	58	83	57	69	11/19/2020
93	28	21	43	100	100	86	100	12/25/2020
84	22	19	43	75	89	86	81	2021/01/08
93	28	21	43	100	100	86	100	1/14/2021
88	19	20	48	63	94	98	76	5/12/2021
84	20	19	45	67	89	90	76	8/27/2021
92	27	19	45	96	89	90	93	9/3/2021
86	23	17	45	79	78	90	79	9/6/2021
57	10	14	35	25	61	67	40	8/16/2021
56	10	15	33	25	67	62	43	8/19/2021
73	16	21	37	50	100	71	71	9/29/2021
71	21	20	33	71	94	62	81	9/20/2021
								5/2/2018
74.5	19.2	18.0	38.5	63.3	83.1	75.0	71.8	

18.1	6.4	3.1	8.2	26.6	17.1	19.5	20.0	

Obtained date (Y/M/D)	Hct	MCV	MCH	MCHC	ALP	E.S.R.	D-dimer test	ALB

2/12/2020	48.3	93.4	30.9	33.1		10	100.29	-
5/22/2020	35.8	69.6	22.2	31.8	44	48	-	-
6/8/2020	42.0	90.1	30.3	33.6	83	41	-	4.1
10/12/2020	35.4	91.0	29.8	32.8		6	281.69	3.9
6/12/2020	43.5	96.7	32.9	34.0	39	6	-	-
8/7/2020	41.1	88.8	29.2	32.8	40	19	160.18	3.4
7/13/2020	37.9	87.5	29.8	34.0	53	37	-	-
9/30/2020	36.0	97.3	32.4	33.3	55	6	312.24	5.1
10/7/2020	43.9	92.4	29.5	31.9	43	43	-	-
10/14/2020	39.9	100	34.1	34.1	72	21	-	-
12/1/2020	35.1	72.2	22.2	30.8	40	130	-	-
1/25/2021	41.4	92.8	30.7	33.1	48	15	-	-
1/23/2021	34.6	92.3	31.2	33.8	31	64	954.42	-
1/14/2021	43.2	85.4	29.6	34.7	54	18	474.14	
5/12/2021	40.7	92.7	31	33.4	67	-	-	-
8/27/2021	39.1	81	27.3	33.8	74	78	-	-
9/3/2021	37.6	89.5	29.5	33	83	21	300.78	4
9/10/2021	38	98.2	32	32.6	53	38	-	4.3
9/13/2021	47.9	93.4	29.6	31.7	38	32	438.93	4.3
9/16/2021	32.9	91.4	29.4	32.2	54	46	424.42	-
9/20/2021	44.3	100.7	33	32.7	54	34	-	-
9/17/2021	41.3	94.5	32	33.9	57	27	-	-
5/2/2018	35.4	98.3	34.2	34.7		88	-	-

C'3	C'4	ANA-pattern Negative=0	ANA-titer 1:_X	Anti-ENA(Jo-1 Ab)	Anti-ENA(Sm)	Anti-ENA(RNP)	Anti-ENA(ScL-70 Ab)	Anti-Cardiolipin-IgG

71.2	19.7	-	-	-	-	-	100.38	-
110	40.8	-	-	-	-	-	-	-
-	-	0	-	-	-	-	-	-
61.4	11.6	-	-	2.52	-	-	9.71	-
78.1	19.3	-	-	-	-	-	5.36	-
90.8	16.2	S	2560	0	26.19	125.06	0	-
-	-	0	-	-	-	-	-	-
85.4	16.6	S	160	0	-	-	0	0
74.2	21.4	S	2560	2.72	3.28	3.72	6.21	-
82.6	16.1	N	320	-	-	-	73.53	-
72	13.5	-	-	-	-	-	-	-
-	-	-	-	-	-	-	-	-
76.9	16.1	S	2560	2.34	4.72	6.19	2.09	11.7
73.1	14.5	S	160	6.44	2.79	4.88	2.91	-
-	-	-	-	-	-	-	-	-
88.4	17	S	2560	10.61	-	-	9.75	-
97.9	24.2	H	320	4.13	19.91	20.28	3.25	-
66.4	16.1	H	640	2.06	2.21	2.34	5.13	-
97.7	21.1	S	2560	2.94	3.54	4.14	137.25	2.65
74.2	15.8	H	640	2.09	2.28	2.37	2.7	3.37
89.5	20.5	0	-	-	-	-	-	-
92.9	26.9	-	-	83.59	-	-	-	-
97.3	13.4	S	2560	-	0	0	-	0

ANCA 1:_X Negative= 0

-
-
-
0
-
-
0
-
0
-
-
-
0
-
-
320
640
0
0
0
0
-
-
